# Synthesis of a New Series of Nitrogen/Sulfur Heterocycles by Linking Four Rings: Indole; 1,2,4-Triazole; Pyridazine; and Quinoxaline

**DOI:** 10.3390/molecules25030450

**Published:** 2020-01-21

**Authors:** Ahmed T. A. Boraei, Ahmed A. M. Sarhan, Sammer Yousuf, Assem Barakat

**Affiliations:** 1 Chemistry Department, Faculty of Science, Suez Canal University, Ismailia 41522, Egypt; 2 Chemistry Department, Faculty of Science, Arish University, Al-Arish 45511, Egypt; ahmed_sarhan252@yahoo.com; 3 H.E.J. Research Institute of Chemistry, International Center for Chemical and Biological Sciences, University of Karachi, Karachi 75270, Pakistan; dr.sammer.yousuf@gmail.com; 4 Chemistry Department, College of Science, King Saud University, P.O. Box 2455, Riyadh 11451, Saudi Arabia; 5 Chemistry Department, Faculty of Science, Alexandria University, P.O. Box 426, Ibrahimia, Alexandria 21321, Egypt

**Keywords:** 1,2,4-Triazolel, indole, pyridazine, quinoxalines, linker, annulated heterocycles

## Abstract

A new series of nitrogen and sulfur heterocyclic systems were efficiently synthesized by linking the following four rings: indole; 1,2,4-triazole; pyridazine; and quinoxaline hybrids. The strength of the acid that catalyzes the condensation of 4-amino-5-(1*H*-indol-2-yl)-2,4-dihydro-3*H*-1,2,4-triazole-3-thione **1** with aromatic aldehydes controlled the final product. Reflux in glacial acetic acid yielded Schiff bases **2**–**6**, whereas concentrated HCl in ethanol resulted in a cyclization product at C-3 of the indole ring to create indolo-triazolo-pyridazinethiones **7**–**16**. This fascinating cyclization approach was applicable with a wide range of aromatic aldehydes to create the target cyclized compounds in excellent yield. Additionally, the coupling of the new indolo-triazolo-pyridazinethiones **7**–**13** with 2,3-bis(bromomethyl)quinoxaline, as a linker in acetone and K_2_CO_3_, yielded 2,3-bis((5,6-dihydro-14*H*-indolo[2,3-d]-6-aryl-[1,2,4-triazolo][4,3-b]pyridazin-3 ylsulfanyl)methyl)quinoxalines **19**–**25** in a high yield. The formation of this new class of heterocyclic compounds in high yields warrants their use for further research. The new compounds were characterized using nuclear magnetic resonance (NMR) and mass spectral analysis. Compound **6** was further confirmed by the single crystal X-ray diffraction technique.

## 1. Introduction

Nitrogen-, oxygen-, and sulfur-containing heterocycles are one of the most important compounds found in organic chemistry, as well as in the pharmaceutical industry [1,2,3,4,5,6]. Among these, 4-amino-1,2,4-triazole-3-thione-based indole scaffolds have drawn considerable attention in the chemical community, including in material science and agrochemical applications. The substituted triazoles have emerged in different pharmaceutical applications including antiproliferative [7], antiviral [8], antimalarial [9], antimicrobial [10], and anticonvulsant agents [11]. Additionally, a few studies have reported their use as inhibitors for metalloenzyme-including ureases [12], dizinc metallo-β-lactamase [13], the tumor necrosis factor alpha (TNF-α) converting enzyme [14], ADAM metallopeptidase with thrombospondin type 1 motif 5 (ADAMTS-5) [15], dicopper dopamine-β-hydroxylase inhibitors [16], and also as human carbonic anhydrase enzyme and acetylcholinesterase (AChE) activities [17].

**Figure 1:**
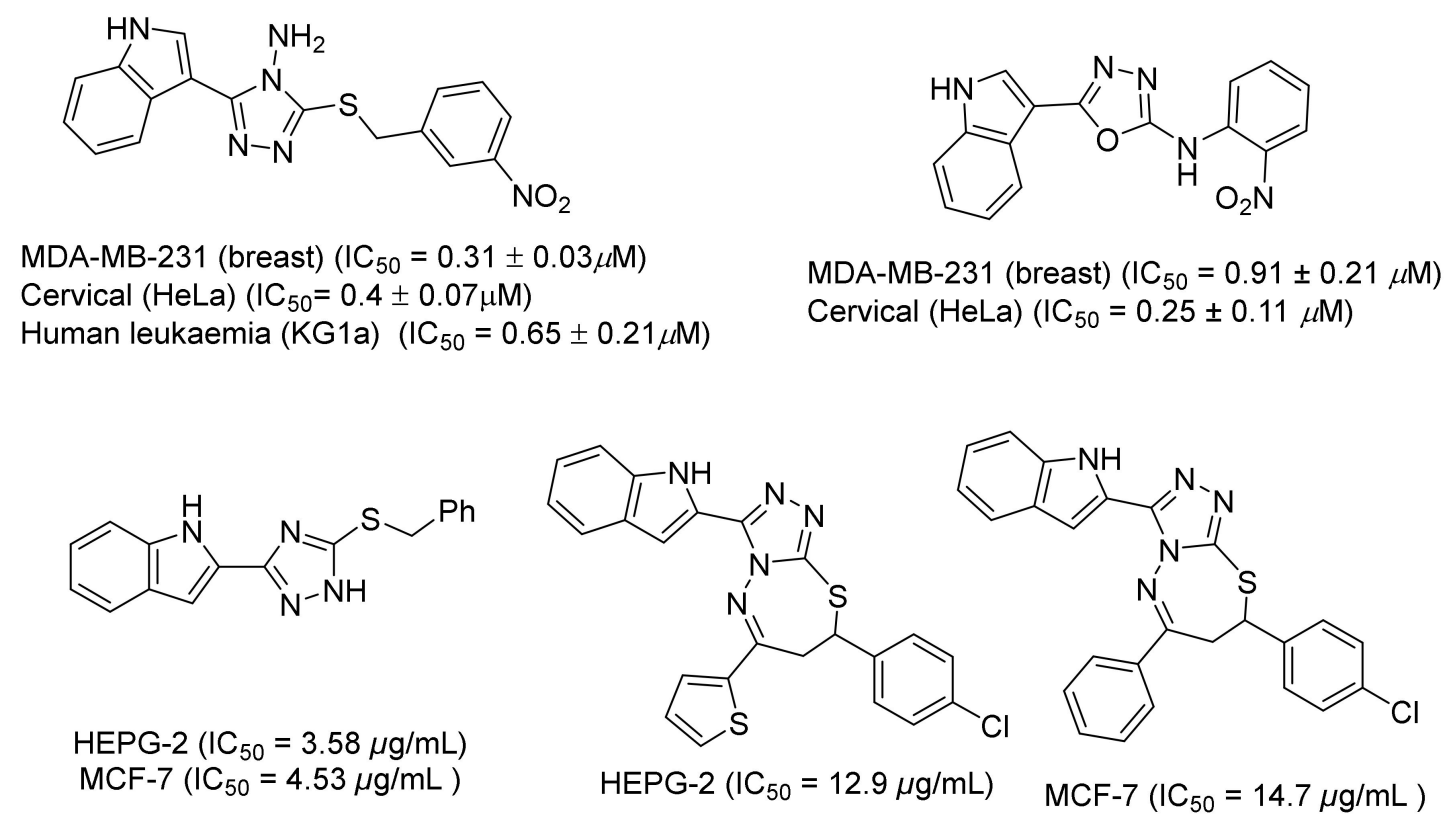
Selected 1,2,4-triazole- indole scaffolds.

Many triazole-based indoles as a core structure are reported with pharmaceutical targets. Westwell, A.D. et al., independently introduced the synthesis of a set of compounds with a 4-amino-1,2,4-triazole-3-thione-based indole moiety via S-arylation; among these series, they discovered that 3-nitrobenzyl derivative has higher activity in Bc1-2-expressing human cancer cell lines at the BH3 binding pocket with (IC_50_ μM) equal to 0.31 ± 0.03 μM for breast (MDA-MB-231), 0.40 ± 0.07 μM for cervical (HeLa), and 0.65 ± 0.21 μM for human leukemia (KG1a), respectively [18]. Another analogue derived from triazole-based indole was reported also by Westwell, A.D. et al., which exhibited anticancer activity against breast MDA-MB-231 and cervical HeLa cell lines; IC_50_ = 0.91 ± 0.21 μM, and 0.25 ± 0.11 µM, respectively [19]. In the same area of research, Boraei, A.T.A. et al. explored a set series of compounds including the S-benzylation of a triazole-based indole. Additionally, another set of triazolo-thiadiazepine and triazolo-thiadiazine scaffolds were discovered as potential inhibitors for epidermal growth factor receptor (EGFR) [20,21] (Figure 1).

A novel fluorophore was derived from a triazole–indole scaffold for a fluorimetric DNA biosensor technique, which was applied for tumor suppressor gene detection and was achieved by Darestani-Farahani, M. et al., in 2018 [22]. Indeed, the indole–triazole Schiff base was synthesized and used as a fluorescent probe for Al^3+^ ions [23]. This research area reported in the literature regarding indole–triazole hybrids has gained attention from a large number of organic chemists, as well as medicinal chemists.

4-Amino-1,2,4-triazole-3-thione-based indole scaffolds have been used in a large number of organic transformations [24,25], including in metal complexation and in applications for conventional Mizoroki–Heck, and Tsuji–Trost reaction catalysis [26]. Triazole–indole hybrids are commonly reported as synthetic intermediates with hydrazonoyl halides for the synthesis of annulation heterocycles [27]. Gomha, S.M. and Riyadh, S.M. independently applied the microwave technique for the synthesis of a set of compounds with triazole–indole–thiadiazole moieties starting from 4-amino-1,2,4-triazole-3-thione-based indole as a building block, and they explored their potential antimicrobial activity [28]. We have noticed, in the specific reaction of triazole–indole as a core structure with aldehydes, that carbon position number 2 in the indole scaffold plays a crucial rule in chemical transformation [29,30]. Furthermore, novel triazole–indole–oxadiazole compounds were synthesized utilizing triazole–indole hit via ultrasound irradiation, and the synthesized compounds exhibited antimicrobial activity against *E. coli* and *S. aureus* with values of (MIC) between 2 and 8 mg/mL [31]. One more example derived from an indolo–trizolo scaffold was reported by Diana, P. et al., which showed remarkable antibiofilm activity against *S. aureus* [32]. The quinoxaline scaffold showed interesting biological activity [33]. Diana, P. et al., synthesized a new series of aza-isoindolo and isoindolo-azaquinoxaline derivatives which have been discovered to be anticancer agents [34,35].

With these findings mentioned above, here, we reported the synthesis of new multi-nitrogen/sulfur heterocyclic systems by linking the following four rings: Indole; 1,2,4-triazole; pyridazine; and quinoxaline.

## 2. Results and Discussion

### 2.1. Synthesis of ***2***–***16*** and ***19***–***25***

**Scheme 1:**
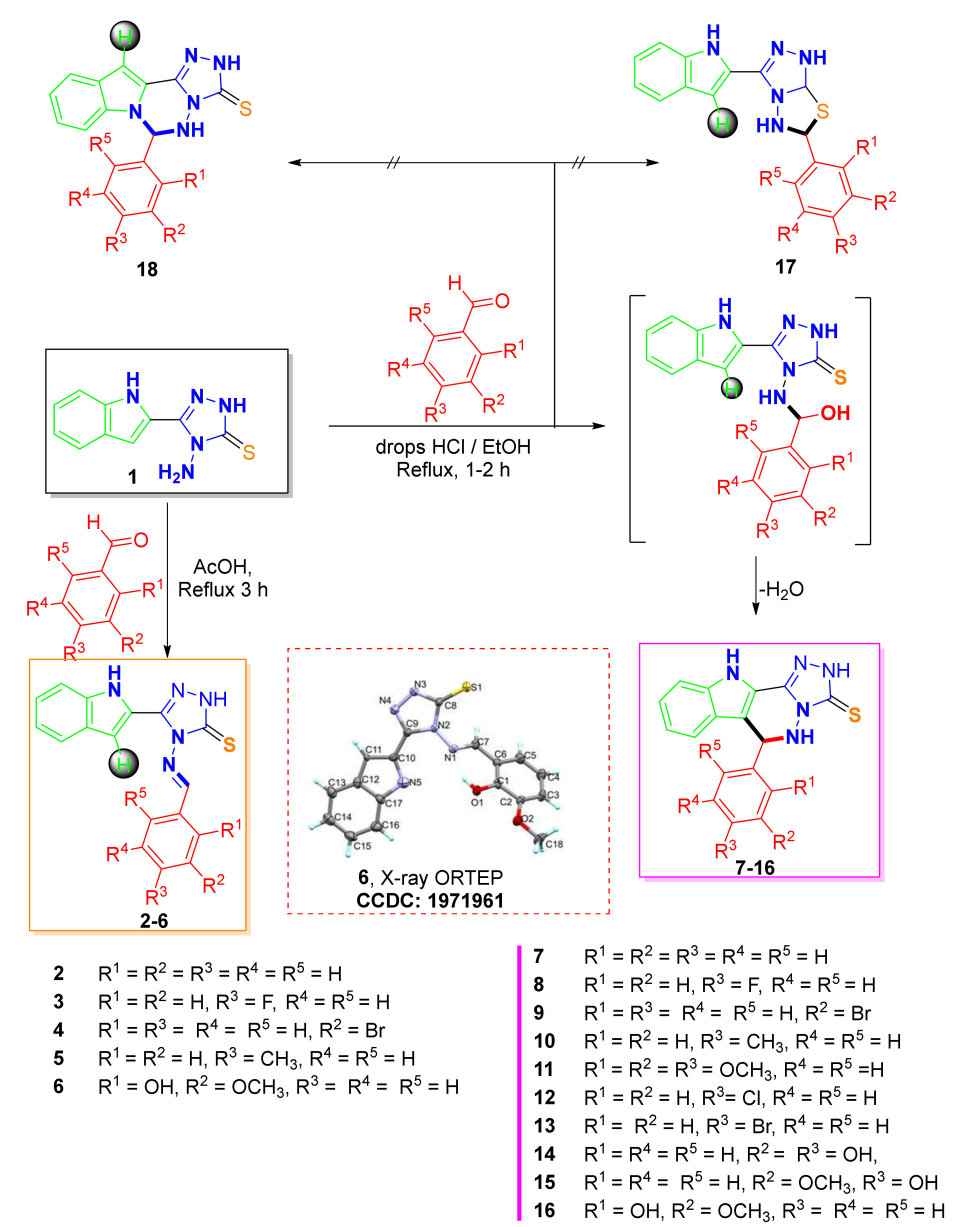
Synthesis of Schiff bases **2**–**6** and cyclized indolo-triazolo-pyridazinethiones **7**–**16**.

The condensation of 4-amino-5-(1*H*-indol-2-yl)-2,4-dihydro-3*H*-1,2,4-triazole-3-thione **1** with benzaldehyde, 4-fluorobenzaldehyde, 3-bromobenzaldehyde, *p-*tolualdehyde, and *o*-vanillin in glacial acetic acid yielded the Schiff bases **2**–**6** in low yield. Alternatively, we carried out the reaction using a stronger acidic medium of concentrated HCl in ethanol under a reflux condition. Surprisingly, this reaction condition exclusively yielded the cyclized compounds indolo-triazolo-pyridazinethiones **7**–**16** in excellent yields. The approach was further extended for the substrate scope, and the reaction was performed with ten aromatic aldehydes with different electronic effects: benzaldehyde, 4-fluorobenzaldehyde, 3-bromobenzaldehyde, *p-*tolualdehyde, 2,3,4-trimethoxybenzaldehyde, 4-chlorobenzaldehyde, 4-bromobenzaldehyde, 3,4-dihydroxybenzaldehyde, 4-hydroxy-3-methoxybenzaldehyde, and 2-hydroxy-3-methoxybenzaldehyde (Scheme 1). Compounds **17** and **18** did not occur under the reaction condition.

**Scheme 2:**
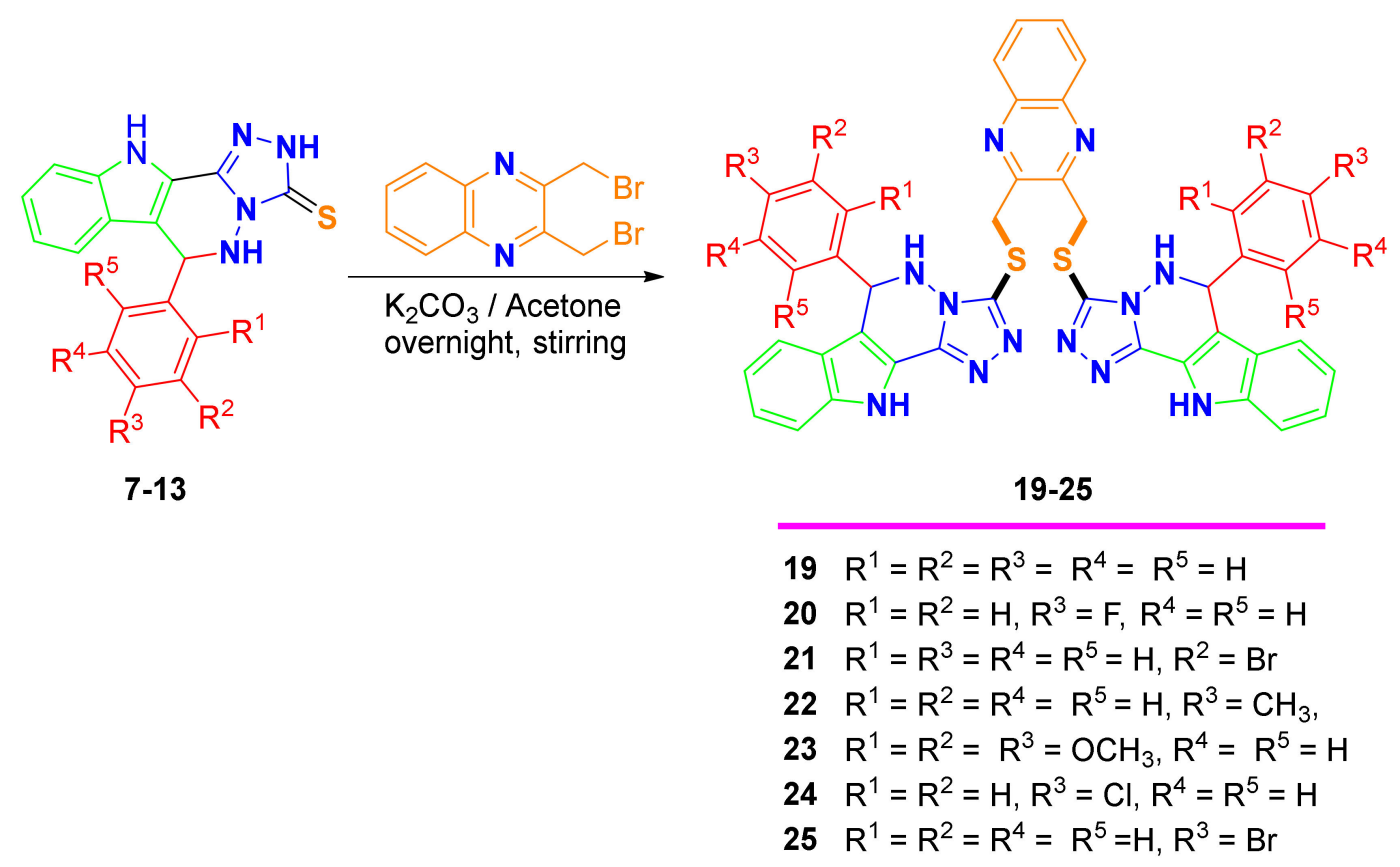
Coupling of indolo-triazolo-pyridazinethiones **7**–**13** with 2,3-*bis*(bromomethyl)quinoxaline.

The coupling of indolo-triazolo-pyridazinethiones **7**–**13** with 2,3-bis(bromomethyl)quinoxaline in acetone and K_2_CO_3_ yielded 2,3-bis((5,6-dihydro-14*H*-indolo[2,3-*d*]-6-aryl-[1,2,4-triazolo][4,3-*b*]pyridazin-3-ylsulfanyl)methyl)quinoxalines **19**–**25** in excellent yields (Scheme 2).

### 2.2. Structural Assignments

**Figure 2:**
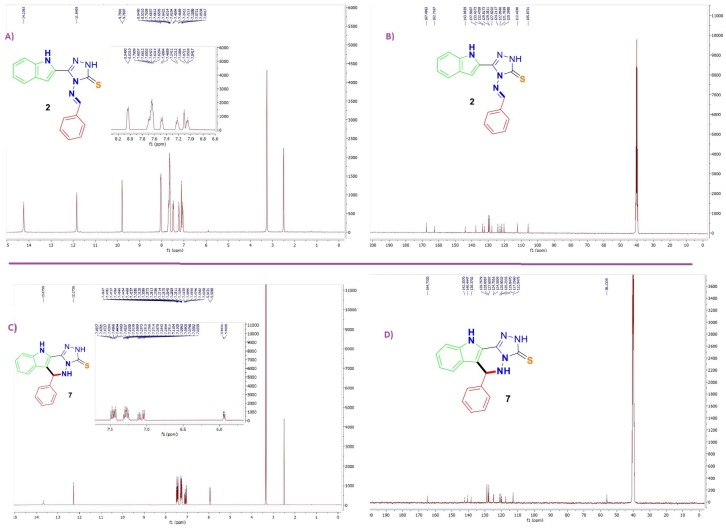
(**A**) ^1^H nuclear magnetic resonance (NMR) of **2**, (**B**) ^13^C-NMR of **2**, (**C**) ^1^H-NMR of **7**, and (**D**) ^13^C-NMR of **7**.

The structural assignments were established based on ^1^H- and ^13^C-NMR, and the differentiation between the two isomers **2** and **7** is discussed by comparing their spectra in Figure 2.

The Schiff base structures **2**–**6** were assigned from their spectra, which showed the following: The aromatic protons appeared in the range from 7.01 to 8.03 ppm, the benzylidene CH proton appeared as singlet around 9.73 ppm, and two D_2_O exchangeable signals were assigned for indole NH near 11.90 ppm, and the second for triazole NH that found around 14.30 ppm. The ^13^C-NMR showed only aromatic and thiocarbonyl signals (C=S) between 105.0 and 167.0 ppm (Figure 2A,B). There was exclusive formation of indolo-triazolo-pyridazinethiones **7**–**16** whereas, indolo-triazolo-thiadiazole **17** and indolo-triazolo-triazine **18** did not occur. The structures assignments were deduced from the ^1^H-NMR, which showed the appearance of pyridazine CH proton as a doublet around 5.92 ppm; then, the aromatic protons and three D_2_O exchangeable signals around 7.01, 12.24, and 13.64 ppm assigned for pyridazine NH, indole NH, and triazole NH protons, respectively, appeared. ^13^C-NMR displayed the pyridazine CH at 56.13 ppm and the thiocarbonyl signal (C=S) around 164.73 ppm (Figure 2C,D). In addition, the disappearance of one CH from the indole signals strongly implied the indolo-triazolo-pyridazinethiones **7**–**16** structures. Structure **17** was excluded because it should contain the indole CH and should not contain any thiocarbonyl signal. Structure **18** was also excluded because our structures contain the indole NH. The ^1^H-NMR of derivatives **19**–**25** displayed the methylene protons around 5.00 ppm, and the CH of the pyridazine ring was detected at 5.92 ppm. Moreover, two exchangeable protons were detected at 7.00 ppm for NH pyridazine and 12.36 ppm for the NH indole. ^13^C-NMR demonstrated the methylene carbons around 35.10 ppm, and no carbons were detected near to 160.00 ppm. The results support the idea that the alkylation of bis(bromomethyl)quinoxaline was performed with sulfur rather than nitrogen.

### 2.3. X-ray Diffraction Analysis of ***6***

**Figure 3:**
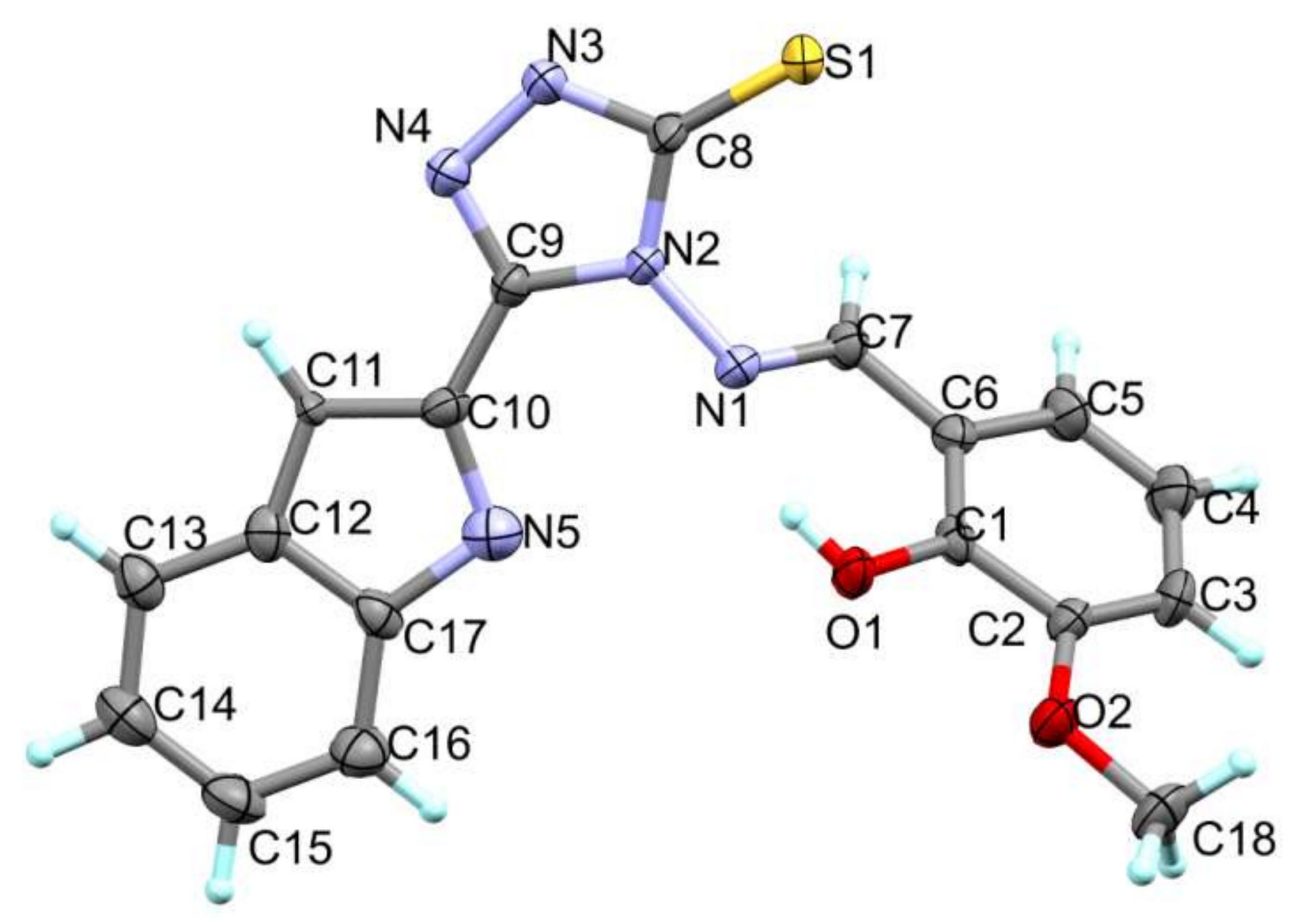
Structure and atom numbering of **6**.

**Figure 4:**
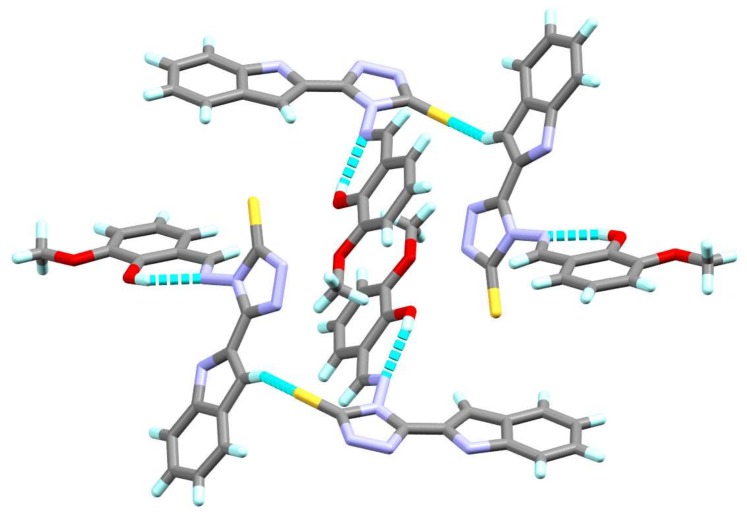
The molecular packing of the synthesized compound **6**. Intra and intermolecular hydrogen bonds are indicated as dashed lines.

The structure of **6** was determined by single crystal X-ray diffraction (Figure 3). Compound **6** crystallized in **a** triclinic system and P-1 space group with Z = 2 and two molecular formula per asymmetric unit (Table 1 and Table 2). For simplicity, one of the two-formula units with atom numbering is shown in Figure 3. The lists of the bond distances and angles are collected in Tables S1 and S2 (Supplementary data). The indole moiety is typically a planar system in which the plane passes through the triazole-3-thione, and the aryl moieties are twisted from the plane of the indole plane by 8.91° and 52.82°, respectively, for the molecular unit shown in Figure 4. The other molecular unit with higher atom numbering showed similar twists of 6.60° and 52.98°, respectively [36,37,38].

The structure showed two intramolecular hydrogen bonds of the type O-H…N between the hydroxyl group as a hydrogen bond donor, the adjacent Schiff base nitrogen atom as a hydrogen bond acceptor, and donor-acceptor distances in the range of 2.659(6)–2.664(6) Å (Table 3). In addition, each of the two units in the crystal are connected by weak C11-H11…S2 hydrogen bonds with a donor-acceptor distance of 3.391(5) Å, leading to the hydrogen-bonded dimeric units shown in Figure 4.

## 3. Materials and Methods

Melting points are determined using a melting-point apparatus (SMP10) in open capillaries and are uncorrected. The progress of the reactions was monitored by thin layer chromatography (Merck). Detections were achieved by UV light illumination. For flash chromatography, commercial silica was used. Nuclear magnetic resonance (^1^H-NMR, ^13^C-NMR, and 2D NMR) spectra were determined in DMSO-*d*_6_ and were recorded on Bruker AC 300/500 spectrometers using TMS as an internal standard. Chemical shifts are termed in δ (ppm) and coupling constants are described in Hz. The assignment of exchangeable OH and NH was confirmed by D_2_O. CHNS-microanalysis was done using a Flash EA-1112 instrument. The HREI mass spectra were detected using a Finnigan MAT 95XP. The FAB-MS was done using Jeol JMS HX110. The IR were detected using a Bruker Alpha ATR-FTIR.

### 3.1. Procedure for ***2***–***6***

The appropriate aldehyde (1.1 mmol) and 4-amino-5-(1*H*-indol-2-yl)-2,4-dihydro-3*H*-1,2,4-triazole-3-thione **1** (1.0 mmol) were refluxed in glacial acetic acid (5.0 mL) for 3 h, then cooled, crystals either appear during cooling then filtered and recrystallized from ethanol or the mixture was poured into cold water; the formed ppt was filtered, dried, and purified by silica-column chromatography using EA/H (1: 1) as an eluent.

*4-(Benzylideneamino)-5-(1H-indol-2-yl)-2H-1,2,4-triazole-3(4H)-thione***2**. Yield 65%; m.p. 221 to 222 °C, ^1^H-NMR (DMSO-*d*_6_, 500 MHz) δ 7.03 (dd, 1 H, *J* 8.0, *J* 7.5 Hz), 7.08 (d, 1 H, *J* 1.0 Hz), 7.20 (dd, 1 H, *J* 7.5, *J* 8.2 Hz), 7.45 (d, 1 H, *J* 8.2 Hz), 7.61 to 7.70 (m, 4 H), 8.02 (d, 2 H, *J* 7.3 Hz), 9.73 (s, 1 H, CH=N), 11.89 (br. s, 1 H, NH_Indole_), 14.28 (br. s, H, NH_Triazole_); ^13^C-NMR (DMSO-*d*_6_, 125 MHz) δ 105.30, 111.88, 119.87, 121.25, 122.34, 123.69, 127.26, 128.87, 129.30, 131.90, 132.95, 136.93, 143.41, 162.09, 167.12; IR (cm^−1^): 1601, 2923, 3223, 3423; elemental analysis calculation for C_17_H_13_N_5_S: C, 63.93; H, 4.10; N, 21.93; S, 10.04 found: C, 63.63; H, 3.95; N, 21.99; S, 10.15.

*(E)-4-(4-Flourobenzylideneamino)-5-(1H-indol-2-yl)-2H-1,2,4-triazole-3(4H)-thione***3.** Yield: 71%; m.p. 227 to 228 °C, ^1^H-NMR (DMSO-*d*_6_, 3400 MHz) δ 7.03 (ddd, 1 H, *J* 8.0, *J* 7.5, *J* 0.9 Hz), 7.09 (d, 1 H, *J* 1.03 Hz), 7.22 (ddd, 1H, *J* 7.5, *J* 8.2, *J* 1.1 Hz), 7.46 to 7.50 (3, 3 H), 7.63 (d, 1 H, *J* 8.0 Hz), 8.11 to 8.15 (m, 2 H), 9.74 (s, 1 H, CH=N), 11.90 (br. s, 1 H, NH_Indole_), 14.30 (br. s, H, NH_Triazole_); ^13^C-NMR (DMSO-*d*_6_, 100 MHz) δ 105.36, 111.93, 116.49, 116.71, 119.93, 121.29, 122.33, 123.76, 127.29, 128.58, 131.50, 131.59, 136.97, 143.43, 162.09, 163.66, 166.15; IR (cm^−1^): 1603, 2927, 3099, 3467; elemental analysis calculation for C_17_H_12_FN_5_S: C, 60.52; H, 3.59; F, 5.63; N, 20.76; S, 9.50 found: C, 60.26; H, 3.66; F, 5.71; N, 21.01; S, 9.55.

*(E)-4-((3-Bromobenzylidene)amino)-5-(1H-indol-2-yl)-2,4-dihydro-3H-1,2,4-triazole-3-thione***4**. Yield: 49%; m.p. 236 to 237 °C, ^1^H-NMR (DMSO-*d*_6_, 400 MHz) δ 7.04 to 7.08 (m, 2 H), 7.22 (dd, 1H, *J* 7.5, *J* 8.2 Hz), 7.47 (d, 1 H, *J* 8.2 Hz), 7.58-7.67 (m, 2 H), 7.89 (d, 1 H, *J* 8.0 Hz), 8.06 (d, 1 H, *J* 7.8 Hz), 8.19 (s, 1 H), 9.80 (s, 1 H, CH=N), 11.91 (br. s, 1 H, NH_Indol_), 14.34 (br. s, H, NH_Triazol_); ^13^C-NMR (DMSO-*d*_6_, 100 MHz) δ 105.93, 112.43, 120.45, 121.81, 122.71, 122.96, 124.29, 127.76, 128.18, 131.80, 132.03, 134.75, 136.01, 137.48, 143.98, 162.60, 166.02; IR (cm^−1^): 1588, 2928, 3087, 3442; elemental analysis calculation for C_17_H_12_BrN_5_S: C, 51.27; H, 3.04; Br, 20.06; N, 17.58; S, 8.05. Found: C, 51.08; H, 3.22; Br, 20.13; N, 17.68; S, 8.11.

*(E)-5-(1H-Indol-2-yl)-4-((4-methylbenzylidene)amino)-2,4-dihydro-3H-1,2,4-triazole-3-thione***5.** Yield: 45%; m.p. 225 to 226 °C, ^1^H-NMR (DMSO-*d*_6_, 300 MHz) δ 2.42 (s, 3 H, CH_3_), 6.99 to 7.05 (m, 2 H), 7.19 (dd, 1H, *J* 7.5, *J* 8.2 Hz), 7.42 to 7.45 (m, 3 H), 7.61 (d, 1 H, J 7.9 Hz), 7.91 (d, 2 H, *J* 7.9 Hz), 9.62 (s, 1 H, CH=N), 11.88 (br. s, 1 H, NH_Indole_), 14.25 (br. s, H, NH_Triazole_); ^13^C-NMR (DMSO-*d*_6_, 100 MHz) δ 21.8, 105.8, 112.4, 120.4, 121.7, 123.0, 124.2, 127.8, 129.4, 129.8, 130.4, 137.5, 143.9, 167.6; IR (cm^−1^): 1602, 2924, 3225, 3420 elemental analysis calculation for C_18_H_15_N_5_S: C, 64.84; H, 4.53; N, 21.01; S, 9.62. Found: C, 64.96; H, 4.67; N, 20.95; S, 9.39.

*(E)-4-((2-Hydroxy-3-methoxybenzylidene)amino)-5-(1H-indol-2-yl)-2,4-dihydro-3H-1,2,4-triazole-3-thione***6.** Yield: 61%; m.p. 230 ot 231 °C, ^1^H-NMR (DMSO-*d*_6_, 500 MHz) δ 3.87 (s, 3 H, CH_3_), 7.03 to 7.07 (m, 3 H), 7.17 to 7.23 (m, 2 H), 7.45 (d, 1 H, *J* 8.4 Hz), 7.59 to 7.63 (m, 2 H), 9.88 (s, 1 H), 9.99 (s, 1 H, CH=N), 11.87 (br. s, 1 H, NH_Indole_), 14.23 (br. s, H, NH_Triazole_); ^13^C-NMR (DMSO-*d*_6_, 75 MHz) δ 56.14 (CH_3_), 105.23, 111.934, 115.78, 1168.52, 118.60, 119.69, 119.91, 121.27, 122.54, 123.69, 127.30, 136.93, 143.49, 148.37, 148.47, 162.07, 163.24; IR (cm^−1^): 1608, 2915, 3227, 3418; elemental analysis calculation for C_18_H_15_N_5_O_2_S: C, 59.17; H, 4.14; N, 8.76; S, 8.77 found: C, 59.43; H, 4.22; N, 18.97; S, 8.75.

### 3.2. General Procedure for the Synthesis of Indolo-Triazolo-Pyridazinethiones ***7***–***16***

To a mixture of indolyltriazolethione **1** (1.0 mmol) in ethanol (5.0 mL), the appropriate aldehyde (1.1 mmol) was added followed by the addition of 5 drops of concentrated HCl, and the mixture was refluxed for 1 to 2 h until a precipitate was formed. The solid product was cooled, filtered, dried, and recrystallized from ethanol.

*5,6-Dihydro-14H-indolo[2,3-d]-6-phenyl-[1,2,4-triazolo][4,3-b]pyridazine-3(2H)thione***7.** Yield: 89%; m.p. >300 °C; ^1^H-NMR (DMSO-*d*_6_, 500 MHz) δ 5.92 (d, 1 H, *J* 5.7 Hz, H-6_Pyridazine_), 7.01 (d, 1 H, *J* 5.7 Hz, HN_Pyridazine_, D_2_O exchangeable), 7.07 (dd, 1 H, *J* ≈ 7.5 Hz), 7.22 to 7.29 (m, 4 H), 7.41-7.48 (m, 4 H), 12.24 (br. s, 1 H, NH_Indole_, D_2_O exchangeable), 13.64 (br. s, 1 H, NH_Triazole_, D_2_O exchangeable); ^13^C-NMR (DMSO-*d*_6_, 125 MHz) δ 55.51 (C-6_Pyridazine_), 112.43, 116.79, 119.27, 119.74, 120.40, 124.08, 124.17, 127.065, 127.49, 128.25, 137.80, 139.91, 141.83, 164.21; IR (cm^−1^): 1628, 2897, 2985, 3067, 3162, 3222; HRMS (EI) calculated for C_17_H_13_N_5_S (M^+^): 319.0892. Found: 319.0902.

*5,6-Dihydro-6-(4-flourophenyl)-14H-indolo[2,3-d]-[1,2,4-triazolo][4,3-b]pyridazine-3(2H)thione***8.** Yield: 80%; m.p. >300 °C; ^1^H-NMR (DMSO-*d*_6_, 300 MHz) δ 5.93 (d, 1 H, *J* 5.4 Hz, H-6_Pyridazine_), 7.04 (d, 1 H, *J* 5.4 Hz, HN_Pyridazine_), 7.07 to 7.15 (m, 3 H), 7.26 (dd, 1 H, *J* 7.5, *J* 8.1 Hz), 7.40 to 7.48 (m, 4 H), 12.27 (br. s, 1H, NH_Indole_), 13.66 (br. s, 1 H, NH_Triazole_); ^13^C-NMR (DMSO-*d*_6_, 75 MHz) δ 54.81 (C-6_Pyridazin_), 112.51, 114.93, 115.21, 116.61, 119.34, 119.72, 120.54, 124.14, 124.19, 129.08, 129.19, 136.12, 137.83, 141.83, 163.14 (C_Ph_), 164.32; IR (cm^−1^): 1604, 1632, 2907, 2960, 3068, 3265; HRMS (EI) calculated for C_17_H_12_N_5_SF (M^+^): 337.0797. Found: 337.0781.

*6-(3-Bromophenyl)-5,6-dihydro-14H-indolo[2,3-d]-[1,2,4-triazolo][4,3-b]pyridazine-3(2H)thione***9.** Yield: 73%; m.p. >300 °C; ^1^H-NMR (DMSO-*d*_6_, 300 MHz) δ 5.99 (d, 1 H, *J* 5.1 Hz, H-6_Pyridazine_), 7.11 to 7.35 (m, 2 H), 7.43 (d, 1 H, *J* 8.1 Hz), 7.48 (d, 1 H, *J* 8.1 Hz), 7.59 (d, 1 H, *J* 7.8 Hz), 7.69 (s, 1 H,), 12.30 (br. s, 1H, NH_Indole_), 13.68 (br. s, 1 H, NH_Triazole_) ^13^C-NMR (DMSO-*d*_6_, 75 MHz) δ 55.10 (C-6_Pyridazin_), 113.05, 116.67, 119.80, 120.18, 121.17, 122.18, 124.65, 124.77, 126.48, 129.08, 130.19, 130.83, 130.98, 138.27, 142.27, 143.34, 164.93; IR (cm^−1^): 1627, 2906, 3075, 3202, 3263; HRMS (EI) calculated for C_17_H_12_N_5_SBr (M^+^): 396.9997. Found: 397.0000.

*5,6-Dihydro-14H-indolo[2,3-d]-6-p-tolyl-5,6-[1,2,4-triazolo][4,3-b]pyridazine-3(2H)thione***10.** Yield: 91%; m.p. >300 °C; ^1^H-NMR (DMSO-*d*_6_, 300 MHz) δ 2.23 (s, 3 H, CH_3_), 5.84 (d, 1 H, *J* 5.8 Hz, H-6_Pyridazine_), 6.93 (d, 1 H, *J* 5.8 Hz, HN_Pyridazine_), 7.04–7.28 (m, 6 H), 7.39 (d, 1 H, *J* 8.0 Hz), 7.46 (d, 1 H, *J* 8.2 Hz), 12.22 (br. s, 1H, NH_Indole_), 13.62 (br. s, 1 H, NH_Triazole_); ^13^C-NMR (DMSO-*d*_6_, 75 MHz) δ 20.62 (CH_3_), 55.44 (C-6_Pyridazin_), 112.45, 116.93, 119.34, 119.77, 120.39, 124.09, 124.18, 127.15, 128.84, 136.76, 136.85, 137.83, 141.89, 164.20; IR (cm^−1^): 1622, 3192, 3322; HRMS (EI) calculated for C_18_H_15_N_5_S (M^+^): 333.1048. Found: 333.1072.

*5,6-Dihydro-14H-indolo[2,3-d]-6-(2,3,4-trimethoxyphenyl)-[1,2,4-triazolo][4,3-b]pyridazine-3(2H)thione***11.** Yield: 93%; m.p. 266 to 267 °C; ^1^H-NMR (DMSO-*d*_6_, 300 MHz) δ 3.72, 3.79, 3.96 (3s, 9 H, 3 OCH_3_), 5.99 (d, 1 H, *J* 7.1 Hz, H-6_Pyridazine_), 6.40 (d, 1 H, *J* 8.8 Hz), 6.63 (d, 1 H, *J* 8.8 Hz), 6.75 (d, 1 H, *J* 7.1 Hz, HN_Pyridazin_), 6.97 (dd, 1 H, *J* 8.0, *J* 7.4 Hz), 7.05 (d, 1 H, *J* 8.0 Hz), 7.20 (dd, 1 H, *J* 7.4, *J* 8.3 Hz), 7.46 (d, 1 H, *J* 8.3 Hz), 12.26 (br. s, 1H, NH_Indole_), 13.63 (br. s, 1 H, NH_Triazole_); ^13^C-NMR (DMSO-*d*_6_, 100 MHz) δ 51.15, 55.70, 60.36, 61.57 (C-6_Pyridazin_, 3 OCH_3_), 107.41, 112.44, 115.44, 119.51, 120.18, 120.27, 122.96, 123.54, 124.06, 124.67, 137.94, 141.77, 141.89, 151.63, 153.42, 163.64; IR (cm^−1^): 1623, 3001, 3175; HRMS (EI) calculated for C_20_H_19_N_5_O_3_S (M^+^): 409.1209. Found: 409.1213.

*6-(4-Chlorophenyl)-5,6-dihydro-14H-indolo[2,3-d]-[1,2,4-triazolo][4,3-b]pyridazine-3(2H)thione***12.** Yield: 68%; m.p. >300 °C; ^1^H-NMR (DMSO-*d*_6_, 300 MHz) δ 5.96 (d, 1 H, *J* 5.4 Hz, H-6_Pyridazine_), 7.08 to 7.14 (m, 2 H), 7.27 (dd, 1 H, *J* 7.5, *J* 8.1 Hz), 7.34 (d, 2 H, *J* 8.4 H), 7.41 (d, 2 H, *J* 8.4 H), 7.48 (d, 1 H, *J* 8.1 Hz), 7.53 (d, 1 H, *J* 7.8 Hz), 12.28 (br. s, 1H, NH_Indole_), 13.67 (br. s, 1 H, NH_Triazole_); ^13^C-NMR (DMSO-*d*_6_, 75 MHz) δ 54.69 (C-6_Pyridazine_), 112.53, 116.32, 119.36, 119.72, 120.60, 124.16, 124.23, 128.28, 128.94, 132.17, 137.82, 139.07, 141.80, 164.36; IR (cm^−1^): 1635, 2915, 3075, 3160, 3268; HRMS (EI) calculated for C_17_H_12_N_5_SCl (M^+^): 353.0502. Found: 353.0500.

*6-(4-Bromophenyl)-5,6-dihydro-14H-indolo[2,3-d]-[1,2,4-triazolo][4,3-b]pyridazine-3(2H)thione***13.** Yield 71%; m.p. >300 °C; ^1^H-NMR (DMSO-*d*_6_, 300 MHz) δ 5.95 (d, 1 H, J 5.1 Hz, H-6_Pyridazine_), 7.10 to 7.55 (m, 9 H), 12.29 (br. s, 1H, NH_Indole_), 13.68 (br. s, 1 H, NH_Triazole_); ^13^C-NMR (DMSO-*d*_6_, 100 MHz) δ 54.75 (C-6_Pyridazine_), 112.55, 116.27, 119.39, 119.74, 120.63, 124.19, 124.26, 129.33, 131.22, 137.85, 139.54, 141.82, 164.38; IR (cm^−1^): 1629, 2907, 3071, 3202, 3268; HRMS (EI) calculated for C_17_H_12_N_5_SBr (M^+^): 396.9997. Found: 396.9964.

*5,6-Dihydro-6-(3,4-dihydroxyphenyl)-14H-indolo[2,3-d]-[1,2,4-triazolo][4,3-b]pyridazine-3(2H)thione***14.** Yield 74%; m.p. >300 °C; ^1^H-NMR (DMSO-*d*_6_, 300 MHz) δ 5.66 (d, 1 H, *J* 6.3 Hz, H-6_Pyridazine_), 6.61 to 6.74 (m, 4 H), 7.03 (dd, 1 H, *J* 8.1 *J* 6.9 Hz), 7.20-7.25 (m, 2 H), 7.45 (d, 1 H, *J* 8.1 Hz), 8.78 (s, 1 H, OH), 8.87 (s, 1 H, OH), 12.18 (br. s, 1 H, NH_Indole_), 13.60 (br. s, 1 H, NH_Triazole_); ^13^C-NMR (DMSO-*d*_6_, 100 MHz) δ 55.89 (C-6_Pyridazine_), 112.38, 115.16, 115.23, 117.08, 118.60, 119.40, 119.90, 120.23, 124.01, 124.16, 130.42, 137.87, 141.86, 144.97, 145.01, 163.90; IR (cm^−1^): 1634, 2908, 3071, 3313; HRMS (EI) calculated for C_17_H_13_O_2_N_5_S (M^+^): 351.0790. Found: 351.0765.

*5,6-Dihydro-6-(4-hydroxy-3-methoxyphenyl)-14H-indolo[2,3-d]-[1,2,4-triazolo][4,3-b]pyridazine-3(2H)thione***15.** Yield 87%; m.p. 300 to 301 °C; ^1^H-NMR (DMSO-*d*_6_, 300 MHz) δ 3.70 (s, 3 H, OCH_3_), 5.75 (d, 1 H, *J* 6.0 Hz, H-6_Pyridazine_), 6.57 to 6.64 (m, 2 H), 6.86 (d, 1 H, *J* 6.0 Hz), 7.07 (dd, 1 H, *J* 7.8, *J* 7.2 Hz), 7.21 to 7.28 (m, 2 H), 7.38 (d, 1 H, *J* 7.8 Hz), 7.45 (d, 1 H, *J* 8.1 Hz), 8.91 (s, 1 H, OH), 12.19 (br. s, 1H, NH_Indole_), 13.62 (br. s, 1 H, NH_Triazole_); ^13^C-NMR (DMSO-*d*_6_, 75 MHz) δ 55.53, 55.64 (C-6_Pyridazine_, OCH_3_), 111.49, 112.43, 115.15, 117.49, 119.25, 119.77, 119.82, 120.36, 124.04, 124.32, 130.40, 137.77, 141.98, 145.89, 147.37, 164.25; IR (cm^−1^): 1633, 2917, 3074, 3167, 3279, 3401; HRMS (EI) calculated for C_18_H_15_N_5_O_2_S (M^+^): 365.0946. Found: 365.0980.

*5,6-Dihydro-6-(2-hydroxy-3-methoxyphenyl)-14H-indolo[2,3-d]-[1,2,4-triazolo][4,3-b]pyridazine-3(2H)thione***16.** Yield 89%; m.p. 200 to 201 °C; ^1^H-NMR (DMSO-*d*_6_, 500 MHz) δ 3.82 (s, 3 H, OCH_3_), 6.15 (d, 1 H, *J* 6.5 Hz, H-6_Pyridazine_), 6.27 (d, 1 H, *J* 7.6 Hz), 6.60 (dd, 1 H, *J* 7.9, *J* 8.0 Hz), 6.87 to 6.91 (m, 2 H), 6.96 (dd, 1 H, *J* 8.0, *J* 7.3 Hz), 7.10 (d, 1 H, *J* 8.0 Hz), 7.21 (dd, 1 H, *J* 7.3, *J* 8.3 Hz), 7.45 (d, 1 H, *J* 8.3 Hz), 9.27 (s, 1 H, OH), 12.24 (br. s, 1H, NH_Indole_), 13.68 (br. s, 1 H, NH_Triazole_); ^13^C-NMR (DMSO-*d*_6_, 125 MHz) δ 51.15 (C-6_Pyridazin_), 55.84 (OCH_3_), 111.51, 112.32, 115.64, 118.75, 119.54, 119.75, 119.87, 120.09, 123.71, 124.01, 125.72, 137.91, 141.15, 143.94, 147.54, 162.67; IR (cm^−1^): 1628, 2920, 3074, 3167, 3280, 3406; HRMS (FAB +ve) calculated for C_18_H_16_N_5_O_2_S (M^+^): 366.1025. Found: 366.091030.

### 3.3. General Procedure for the Alkylation with Di(bromomethyl)quinoxaline ***19***–***25***

The appropriate indolo-triazolo-pyridazinethiones (2.0 mmol) and K_2_CO_3_ (2.2 mmol) were stirred in acetone (10 mL) for 1 h; then, di(bromomethyl)quinoxaline (1.1 mmol) was added and stirring was continued overnight. The solvent was removed under vacuum, water was added, and the solid was obtained by filtration, dried, and recrystallized from dimethyl formamide (DMF).

*2,3-Bis((5,6-Dihydro-14H-indolo[2,3-d]-6-phenyl-[1,2,4-triazolo][4,3-b]pyridazin-3-ylsulfanyl)methyl)quinoxaline***19.** Yield: 83%; m.p. 291 to 292 °C; ^1^H-NMR (DMSO-*d*_6_, 300 MHz) δ 5.04 (s, 4 H, 2 SCH_2_), 5.91 (d, 2 H, *J* 6.3 Hz, 2 H-6_Pyridazine_), 7.40 (dd, 2 H, *J ≈* 7.5 Hz), 7.20 to 7.36 (m, 16 H), 7.47 (d, 2 H, *J* 8.1 Hz), 7.80 to 7.83 (m, 2 H), 8.00-8.03 (m, 2 H), 12.34 (br. s, 2 H, 2 NH_Indole_); ^13^C-NMR (DMSO-*d*_6_, 75 MHz) δ 35.86 (2 SCH_2_), 56.54 (2 C-6_Pyridazine_), 112.85, 114.53, 119.94, 120.71, 121.55, 123.92, 124.96, 127.73, 128.13, 128.71, 128.83, 130.74, 137.91, 140.29, 140.53, 146.53, 149.00, 151.10; IR (cm^−1^): 1611, 2926, 3143; elemental analysis calculated for C_44_H_32_N_12_S_2_: C, 66.65; H, 4.07; N, 21.20; S, 8.09. Found: C, 66.93; H, 4.15; N, 21.10; S, 8.15.

*2,3-Bis((5,6-Dihydro-6-(4-flourophenyl)-14H-indolo[2,3-d]-[1,2,4-triazolo][4,3-b]pyridazin-3-ylsulfanyl)methyl)quinoxaline***20.** Yield: 87%; m.p. 294_decomp_. °C; ^1^H-NMR (DMSO-*d*_6_, 300 MHz) δ 5.045 (s, 4 H, 2 SCH_2_), 5.92 (d, 2 H, *J* 6.0 Hz, 2 H-6_Pyridazine_), 7.03 to 7.11 (m, 6 H), 7.22 (dd, 2 H, *J* 7.2, *J* 8.1 Hz), 7.29 to 7.38 (m, 8 H), 7.47 (d, 2 H, *J* 8.1 Hz), 7.80 to 7.83 (m, 2 H), 7.98 to 8.02 (m, 2 H), 12.36 (br. s, 2 H, 2 NH_Indole_); ^13^C-NMR (DMSO-*d*_6_, 75 MHz) δ 35.84 (2 SCH_2_), 55.92 (2 C-6_Pyridazine_), 112.89, 114.28, 119.87, 120.80, 121.60, 123.99, 124.88, 128.71, 129.75, 129.84, 130.74, 136.51, 137.92, 140.51, 146.47, 149.03, 151.10; IR (cm^−1^): 1608, 3060, 3147; HRMS (FAB +ve) calculated for C_44_H_31_N_12_S_2_F_2_ (M^+^): 829.2204. Found: 829.2220.

*2,3-Bis((6-(3-Bromophenyl)-5,6-dihydro-14H-indolo[2,3-d]-[1,2,4-triazolo][4,3-b]pyridazin-3-ylsulfanyl)methyl)quinoxaline***21.** Yield: 79%; m.p. 298_decomp._ °C; ^1^H-NMR (DMSO-*d*_6_, 300 MHz) δ 5.05 (s, 4 H, 2 SCH_2_), 5.99 (d, 2 H, *J* 5.7 Hz, 2 H-6_Pyridazine_), 7.09 (dd, 2 H, *J* 7.8, *J* 7.5 Hz), 7.18 to 7.52 (m, 16 H), 7.80 to 7.83 (m, 2 H), 7.98 to 8.02 (m, 2 H), 12.39 (br. s, 2 H, 2 NH_Indole_); ^13^C-NMR (DMSO-*d*_6_, 75 MHz) δ 35.36 (2 SCH_2_), 55.22 (2 C-6_Pyridazine_), 112.44, 113.27, 119.34, 120.45, 121.06, 121.65, 123.60, 124.38, 128.23, 129.89, 130.24, 130.48, 130.60, 137.37, 140.00, 142.74, 145.87, 148.62, 150.53; IR (cm^−1^): 1609, 2924, 3058; HRMS (FAB +ve) calculated for C_44_H_31_N_12_S_2_Br_2_ (M^+^): 949.0603. Found: 949.0615.

*2,3-Bis((5,6-Dihydro-14H-indolo[2,3-d]-6-p-tolyl-[1,2,4-triazolo][4,3-b]pyridazin-3-ylsulfanyl)methyl)quinoxaline***22.** Yield: 78%; m.p. 284 to 285 °C; ^1^H-NMR (DMSO-*d*_6_, 300 MHz) δ 2.18 (s, 6 H, 2 CH_3_), 5.02 (s, 4 H, 2 SCH_2_), 5.83 (d, 2 H, *J* 6.6 Hz, 2 H-6_Pyridazine_), 7.00 to 7.32 (m, 16 H), 7.46 (d, 2 H, *J* 8.4 Hz), 7.80 to 7.83 (m, 2 H), 7.99-8.01 (m, 2 H), 12.31 (br. s, 2 H, 2 NH_Indole_); ^13^C-NMR (DMSO-*d*_6_, 75 MHz) 21.08 (2 CH_3_), 35.79 (2 SCH_2_), 56.52 (2 C-6_Pyridazine_), 112.82, 114.67, 119.93, 120.65, 121.58, 123.88, 124.93, 127.75, 128.72, 129.36, 130.72, 137.20, 137.35, 137.91, 140.53, 146.56, 148.97, 151.14; IR (cm^−1^): 1610, 2917; HRMS (FAB +ve) calculated for C_46_H_37_N_12_S_2_ (M^+^): 821.2767. Found: 821.2767.

2,3-Bis((5,6-Dihydro-14H-indolo[2,3-d]-6-(2,3,4-trimethoxyphenyl)-[1,2,4-triazolo][4,3-b]pyridazin-3-ylsulfanyl)methyl)quinoxaline **23.** Yield: 90%; m.p. 280 to 283 °C; ^1^H-NMR (DMSO-d_6_, 400 MHz) δ 3.71, 3.78, 3.92 (3s, 18 H, 6 OCH_3_), 4.98 (s, 4 H, 2 SCH_2_), 6.02 (d, 2 H, J 8.0 Hz, 2 H-6_Pyridazine_), 6.48 (d, 2 H, J 8.4 Hz), 6.64 (d, 2 H, J 8.4 Hz), 6.97 (br, 4 H), 7.11 (d, 2 H, J 8.4 Hz), 7.18 (br, 2 H), 7.45 (d, 2 H, J 8.0 Hz), 7.78 (br, 2 H), 7.93 (br, 2 H), 12.34 (br. s, 2 H, 2 NH_Indole_); ^13^C-NMR (DMSO-d_6_, 100 MHz) δ 35.15 (2 SCH_2_), 51.60, 55.71, 60.37, 61.51 (2 C-6_Pyridazin_, 6 OCH_3_), 107.61, 112.33, 113.40, 119.21, 119.98, 121.89, 123.07, 123.34, 123.81, 124.41, 128.17, 130.23, 137.57, 140.02, 141.87, 146.22, 148.23, 150.68, 151.58, 153.39; IR (cm^−1^): 1605, 2931, 3199; HRMS (FAB +ve) calculated for C_50_H_45_N_12_O_6_S_2_ (M^+^): 973.3026. Found: 973.3053.

2,3-Bis((6-(4-Chlorophenyl)-5,6-dihydro-14H-indolo[2,3-d]-[1,2,4-triazolo][4,3-b]pyridazin-3-ylsulfanyl)methyl)quinoxaline **24.** Yield: 77%; m.p. 294_decomp._ °C; ^1^H-NMR (DMSO-d_6_, 300 MHz) δ 5.05 (s, 4 H, 2 SCH_2_), 5.95 (d, 2 H, J 6.0 Hz, 2 H-6_Pyridazine_), 7.07 (dd, 2 H, J 7.5 Hz), 7.20 to 7.48 (m, 16 H), 7.80 to 7.84 (m, 2 H), 7.98 to 8.01 (m, 2 H), 12.37 (br. s, 2 H, 2 NH_Indol_); ^13^C-NMR (DMSO-d_6_, 75 MHz) 35.81 (2 SCH_2_), 55.76 (2 C-6_Pyridazine_), 112.90, 113.98, 119.86, 120.85, 121.61, 124.90, 128.71, 128.84, 129.57, 130.75, 132.76, 137.91, 139.44, 140.50, 146.41, 149.06, 151.10; IR (cm^−1^): 1600, 2927, 3205; HRMS (FAB +ve) calculated for C_44_H_31_N_12_S_2_Cl_2_ (M^+^): 861.1613. Found: 861.1607.

*2,3-Bis((6-(4-Bromophenyl)-5,6-dihydro-14H-indolo[2,3-d]-[1,2,4-triazolo][4,3-b]pyridazin-3-ylsulfanyl)methyl)quinoxaline***25.** Yield: 81%; m.p. 284 to 285 °C; ^1^H-NMR (DMSO-*d*_6_, 300 MHz) δ 5.05 (s, 4 H, 2 SCH_2_), 5.94 (d, 2 H, *J* 5.7 Hz, 2 H-6_Pyridazine_), 7.08 (dd, 2 H, *J*7.5 Hz), 7.21 to 7.49 (m, 16 H), 7.80 to 7.84 (m, 2 H), 7.98 to 8.01 (m, 2 H), 12.37 (br. s, 2 H, 2 NH_Indole_); ^13^C-NMR (DMSO-*d*_6_, 75 MHz) 35.82 (2 SCH_2_), 55.80 (2 C-6_Pyridazin_), 112.91, 113.92, 119.86, 120.86, 121.35, 124.04, 124.91, 128.71, 129.93, 130.74, 131.77, 137.92, 139.88, 140.51, 146.41, 149.08, 151.09; IR (cm^−1^): 1602, 2925, 3198; HRMS (FAB +ve) calculated for C_44_H_31_N_12_S_2_Br_2_ (M^+^): 949.0603. Found: 949.0669.

## 4. Conclusions

A fascinating cyclization reaction approach was achieved when 4-amino-5-(1*H*-indol-2-yl)-2,4-dihydro-3*H*-1,2,4-triazole-3-thione **1** was reacted with ten aromatic aldehydes in ethanol and concentrated HCl to obtain indolo-triazolo-pyridazinethiones **7**–**16** in an excellent yield. Moreover, the alkylation of indolo-triazolo-pyridazinethiones **7**–**13** with 2,3-bis(bromomethyl)quinoxaline as a linker in acetone and K_2_CO_3_ afforded 2,3-bis((5,6-dihydro-14*H*-indolo[2,3-*d*]-6-aryl-[1,2,4-triazolo][4,3-*b*]pyridazin-3 ylsulfanyl)methyl)quinoxalines **19–25** in a high yield. Studies to establish their applications are in progress.

## Figures and Tables

**Table 1:** Crystal data for **6**.

Chemical formula	C_38_H_26_N_10_O_4_S_2_
Formula weight	726.79 g/mol
Temperature	104(2) K
Wavelength	1.54178 Å
Crystal size	0.030 × 0.070 × 0.130 mm
Crystal habit	Yellow plate
Crystal system	Triclinic
Space group	P -1
Unit cell dimensions	a = 10.1105(6) Å
	b = 13.1764(6) Å
	c = 14.2858(6) Å
Volume	1705.44(15) Å^3^
Z	2
Density (calculated)	1.415 g/cm^3^
Absorption coefficient	1.893 mm^−1^
F(000)	752

**Table 2:** Data collection and structure refinement for **6**. (RMS, root mean square)

Theta range for data collection	3.38 to 68.23°
Index ranges	−12 ≤ h ≤ 12, −15 ≤ k ≤ 15, −17 ≤ l ≤ 17
Reflections collected	54525
Independent reflections	6232 [R(int) = 0.1173]
Coverage of independent reflections	99.9%
Absorption correction	Multi-scan
Max. and min. transmission	0.9470 and 0.7960
Structure solution technique	direct methods
Structure solution program	SHELXT 2014/5 (Sheldrick, 2014)
Refinement method	Full-matrix least-squares on F^2^
Refinement program	SHELXL-2017/1 (Sheldrick, 2017)
Function minimized	Σ w(F_o_^2^ − F_c_^2^)^2^
Data/restraints/parameters	6232/0/471
Goodness-of-fit on F^2^	1.085
Final R indices	4104 data; I > 2σ(I)
	all data
Weighting scheme	w = 1/[σ^2^(F_o_^2^) + (0.1000P)^2^] where P = (F_o_^2^ + 2F_c_^2^)/3
Largest diff. peak and hole	1.57 and −0.52 eÅ^−3^
RMS deviation from mean	0.110 eÅ^−3^

**Table 3:** Hydrogen bond parameters for **6**.

D-H…A	D-H	H…A	D…A	D-H…A
O1-H1…N1	0.84	1.94	2.659(6)	143
O3-H3A…N6	0.84	1.95	2.664(6)	143
C11-H11…S2	0.95	2.51	3.391(5)	154

## Supplementary Materials

NMR spectra (Figures S1–S44); and IR analyses (Figures S45–S60) associated with this article can be found in the online version.
