# Supplementary material for: Synthesis of a New Series of Nitrogen/Sulfur Heterocycles by Linking Four Rings: Indole; 1,2,4-Triazole; Pyridazine; and Quinoxaline
Source: Molecules. 2020 Jan 21;25(3):450. doi: 10.3390/molecules25030450 (PMC7037119; doi:10.3390/molecules25030450)
Supplement: Supplementary file 1 [file molecules-25-00450-s001.pdf]

Synthesis of a New Series of Nitrogen/Sulfur Heterocycles by Linking Four Rings: Indole; 1,2,4-Triazole; Pyridazine; and Quinoxaline

Ahmed T. A. Boraei [1,*], Ahmed A. Sarhan [2], Sammer Yousuf [3], and Assem Barakat [4,5,*]

[1] Chemistry Department, Faculty of Science, Suez Canal University, Ismailia 41522, Egypt.
[2] Chemistry Department, Faculty of Science, Al-Arish University, Al-Arish 45511, Egypt. ahmed_sarhan252@yahoo.com
[3] H.E.J. Research Institute of Chemistry, International Center for Chemical and Biological Sciences, University of Karachi, Karachi 75270, Pakistan. dr.sammer.yousuf@gmail.com
[4] Chemistry Department, College of Science, King Saud University, P.O. Box 2455, Riyadh, 11451, Saudi Arabia
[5] Chemistry Department, Faculty of Science, Alexandria University, P.O. Box 426, Ibrahimia, Alexandria 21321, Egypt
* Correspondence: ahmed_tawfeek83@yahoo.com (A.T.A.B); ambarakat@ksu.edu.sa (A.B.); Tel.: +966-11467-5901 (A.B.); Fax: +966-11467-5992 (A.B.).

NMR Spectra

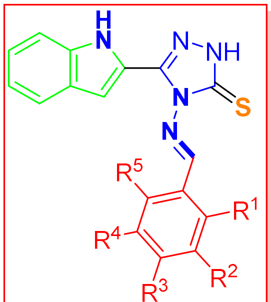

2-6

2 $R^1 = R^2 = R^3 = R^4 = R^5 = H$

3 $R^1 = R^2 = H$, $R^3 = F$, $R^4 = R^5 = H$

4 $R^1 = R^3 = R^4 = R^5 = H$, $R^2 = Br$

5 $R^1 = R^2 = H$, $R^3 = CH_3$, $R^4 = R^5 = H$

6 $R^1 = OH$, $R^2 = OCH_3$, $R^3 = R^4 = R^5 = H$

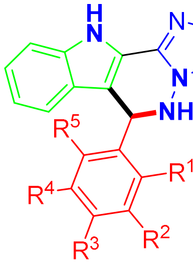

7-16

7 $R^1 = R^2 = R^3 = R^4 = R^5 = H$

8 $R^1 = R^2 = H$, $R^3 = F$, $R^4 = R^5 = H$

9 $R^1 = R^3 = R^4 = R^5 = H$, $R^2 = Br$

10 $R^1 = R^2 = H$, $R^3 = CH_3$, $R^4 = R^5 = H$

11 $R^1 = R^2 = R^3 = OCH_3$, $R^4 = R^5 = H$

12 $R^1 = R^2 = H$, $R^3 = Cl$, $R^4 = R^5 = H$

13 $R^1 = R^2 = H$, $R^3 = Br$, $R^4 = R^5 = H$

14 $R^1 = R^4 = R^5 = H$, $R^2 = R^3 = OH$,

15 $R^1 = R^4 = R^5 = H$, $R^2 = OCH_3$, $R^3 = OH$

16 $R^1 = OH$, $R^2 = OCH_3$, $R^3 = R^4 = R^5 = H$

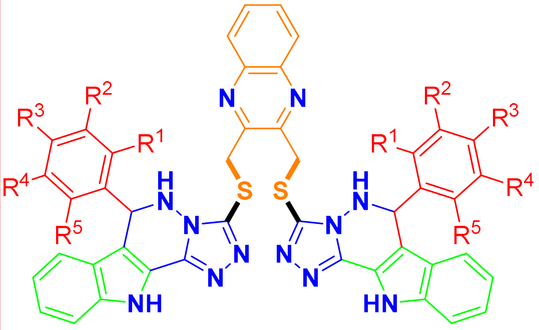

19-25

19 $R^1 = R^2 = R^3 = R^4 = R^5 = H$

20 $R^1 = R^2 = H$, $R^3 = F$, $R^4 = R^5 = H$

21 $R^1 = R^3 = R^4 = R^5 = H$, $R^2 = Br$

22 $R^1 = R^2 = R^4 = R^5 = H$, $R^3 = CH_3$,

23 $R^1 = R^2 = R^3 = OCH_3$, $R^4 = R^5 = H$

24 $R^1 = R^2 = H$, $R^3 = Cl$, $R^4 = R^5 = H$

25 $R^1 = R^2 = R^4 = R^5 = H$, $R^3 = Br$

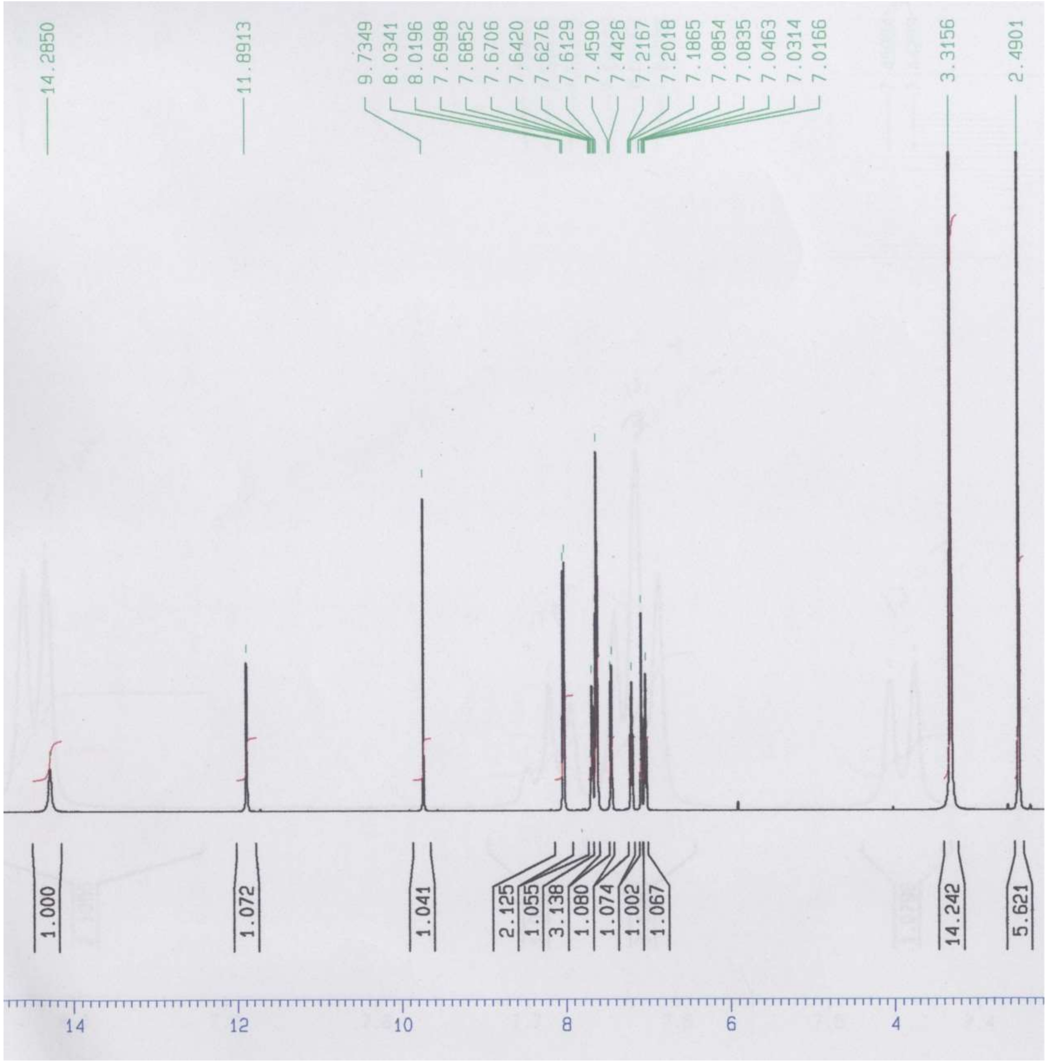

Figure S1. ^{1}H NMR of **2**

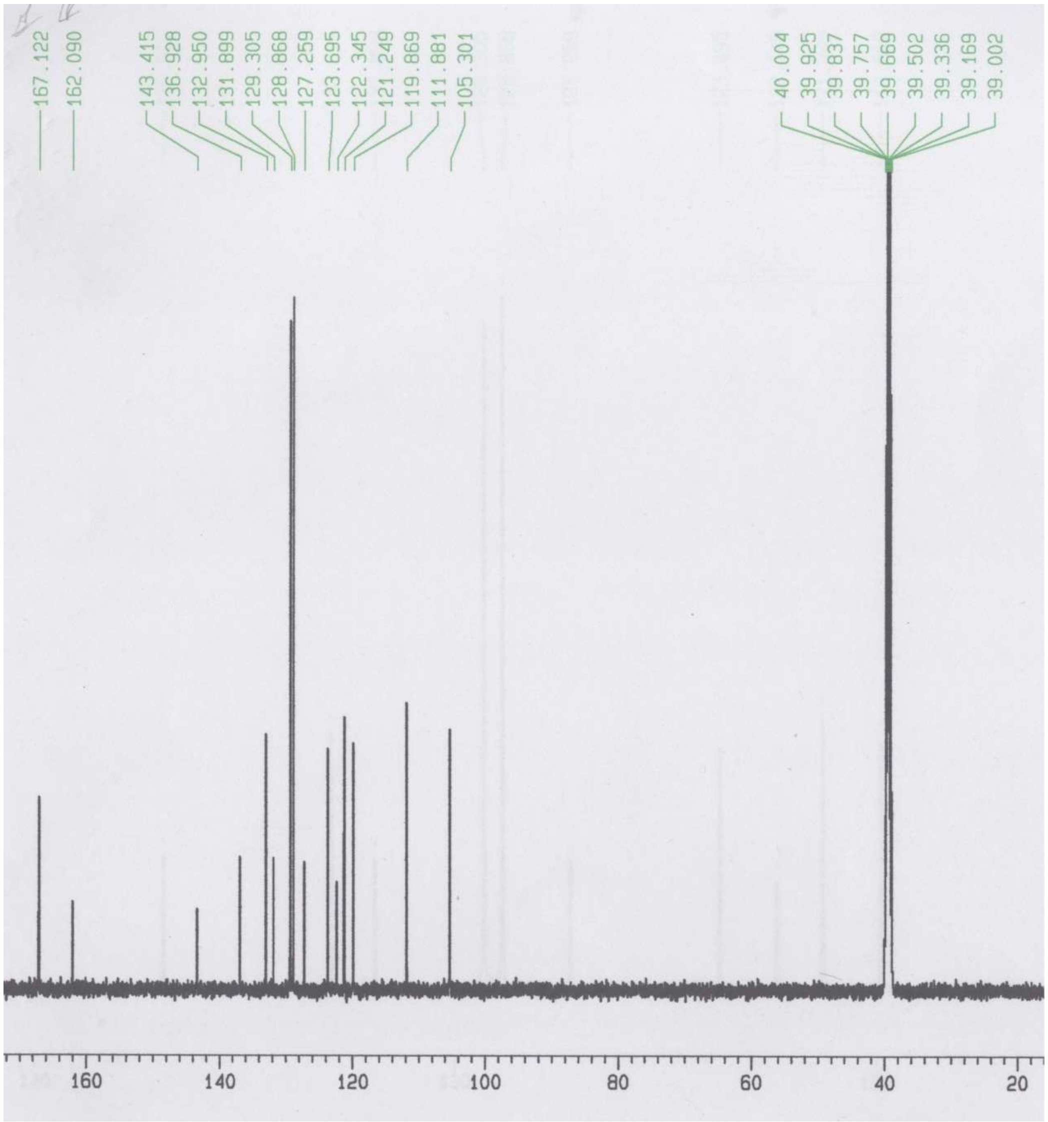

Figure S2. ^{13}C NMR of **2**

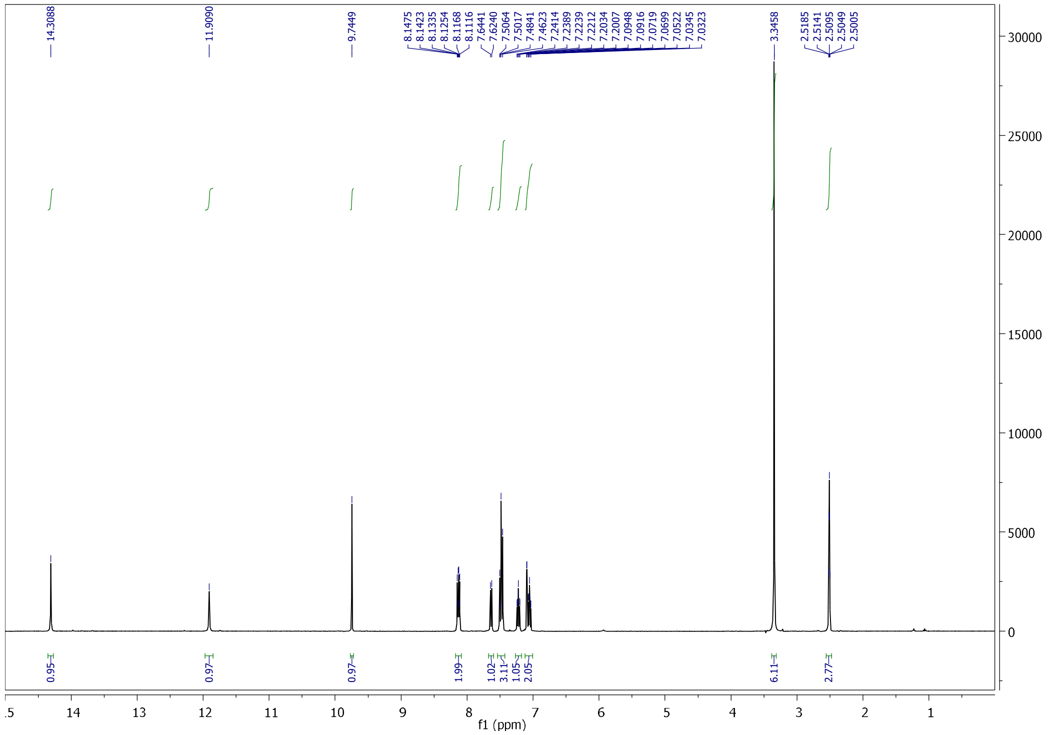

Figure S3. **^{1}H NMR of 3**

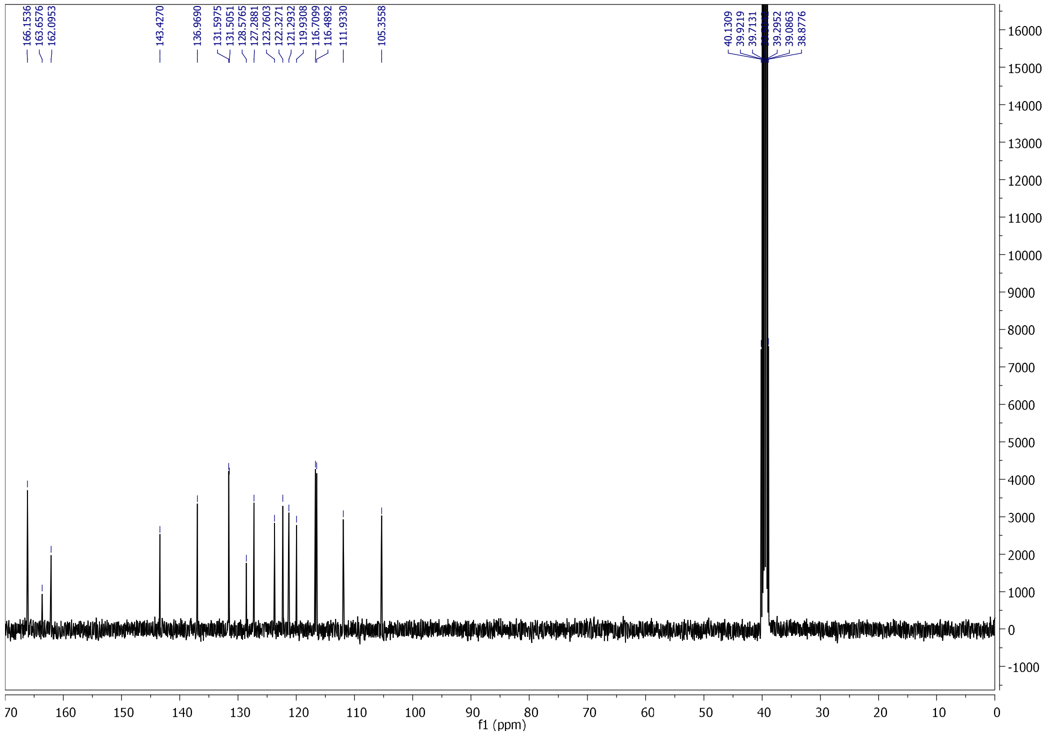

Figure S4. ^{13}C NMR of **3**

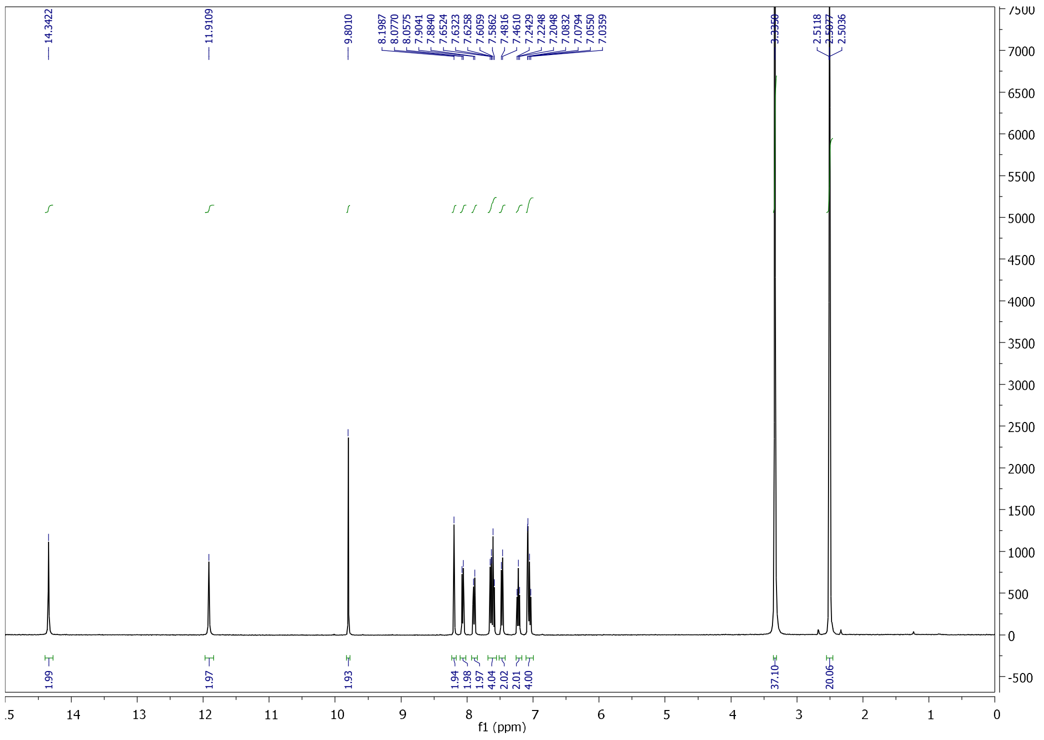

Figure S5. ^{1}**H NMR of 4**

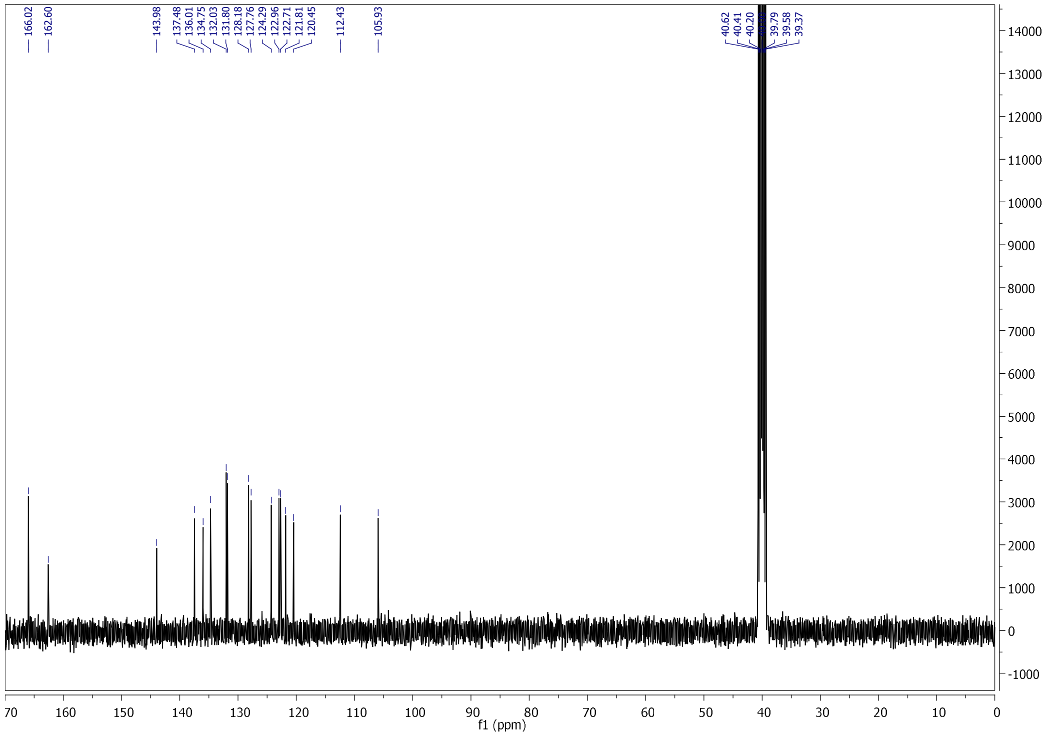

Figure S6. ^{13}C NMR of **4**

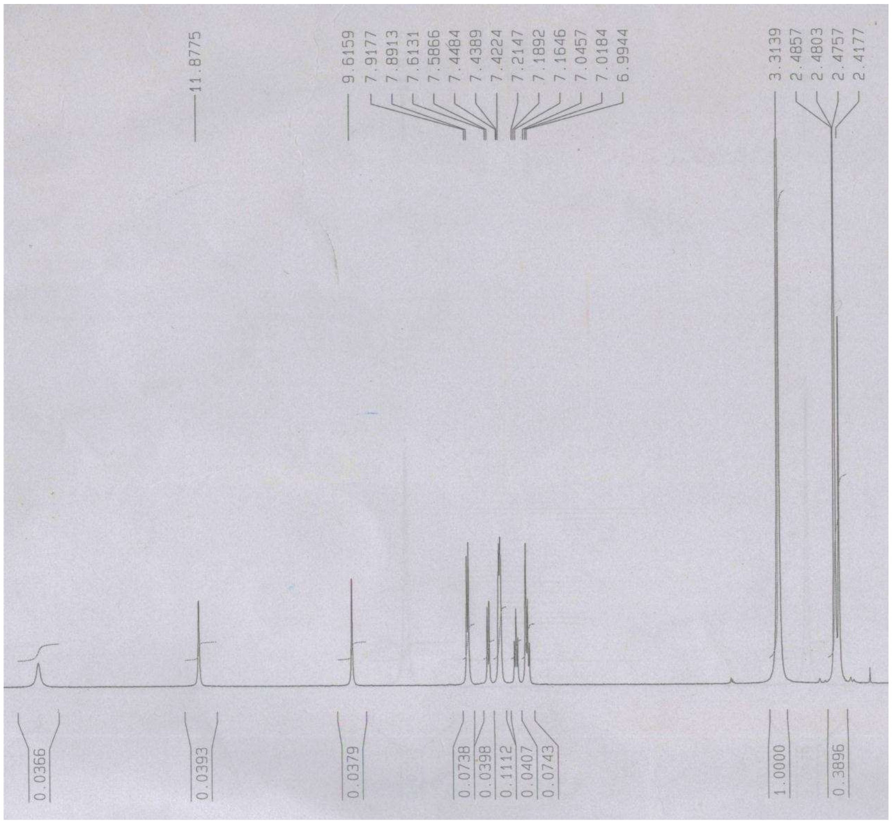

Figure S7. ^{1}H NMR of **5**

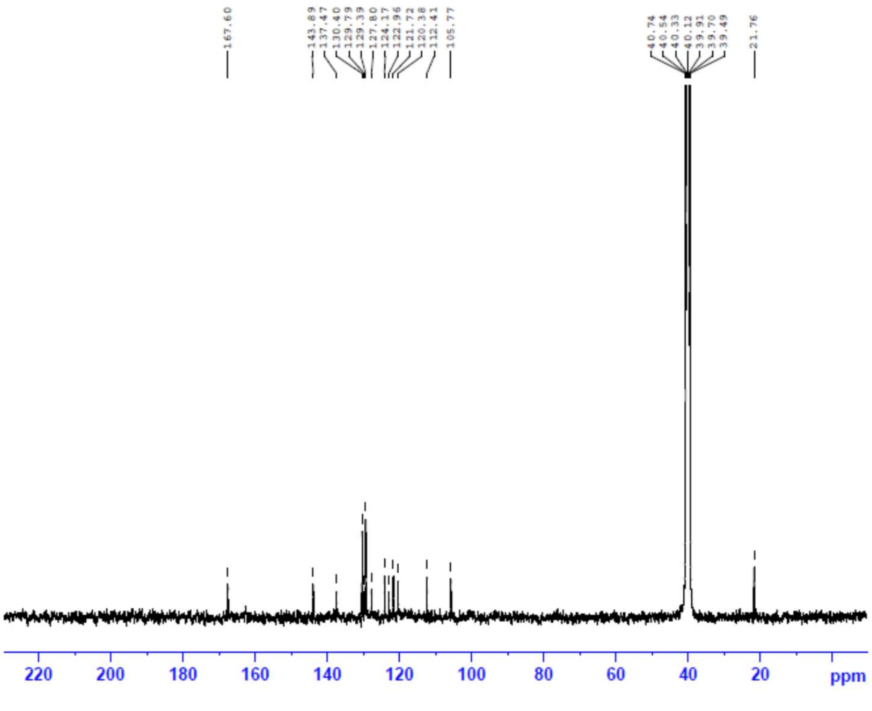

Figure S8. ^{13}C NMR of **5**

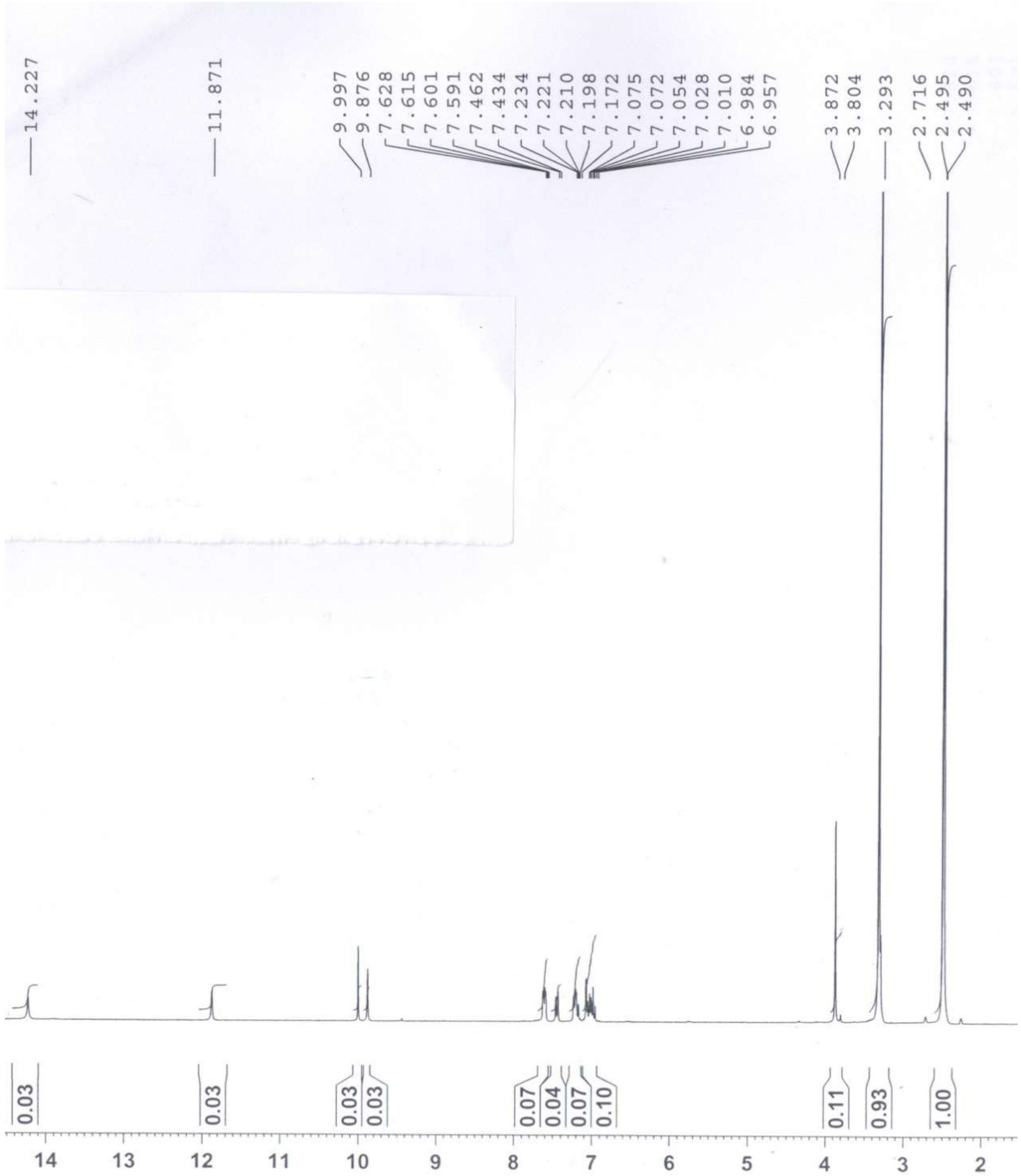

Figure S9. ^{1}H NMR of **6**

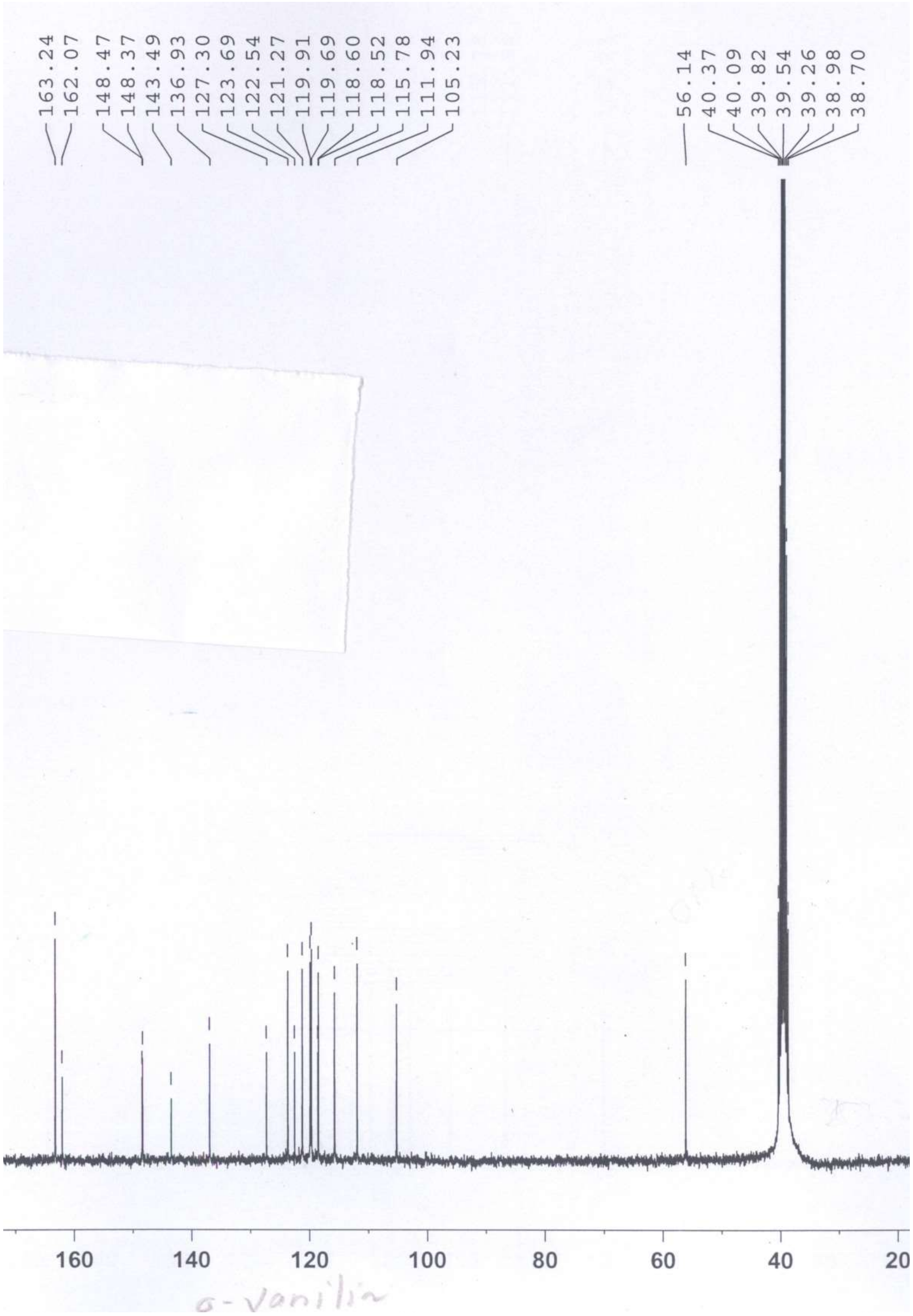

Figure S10. ^{13}C NMR of **6**

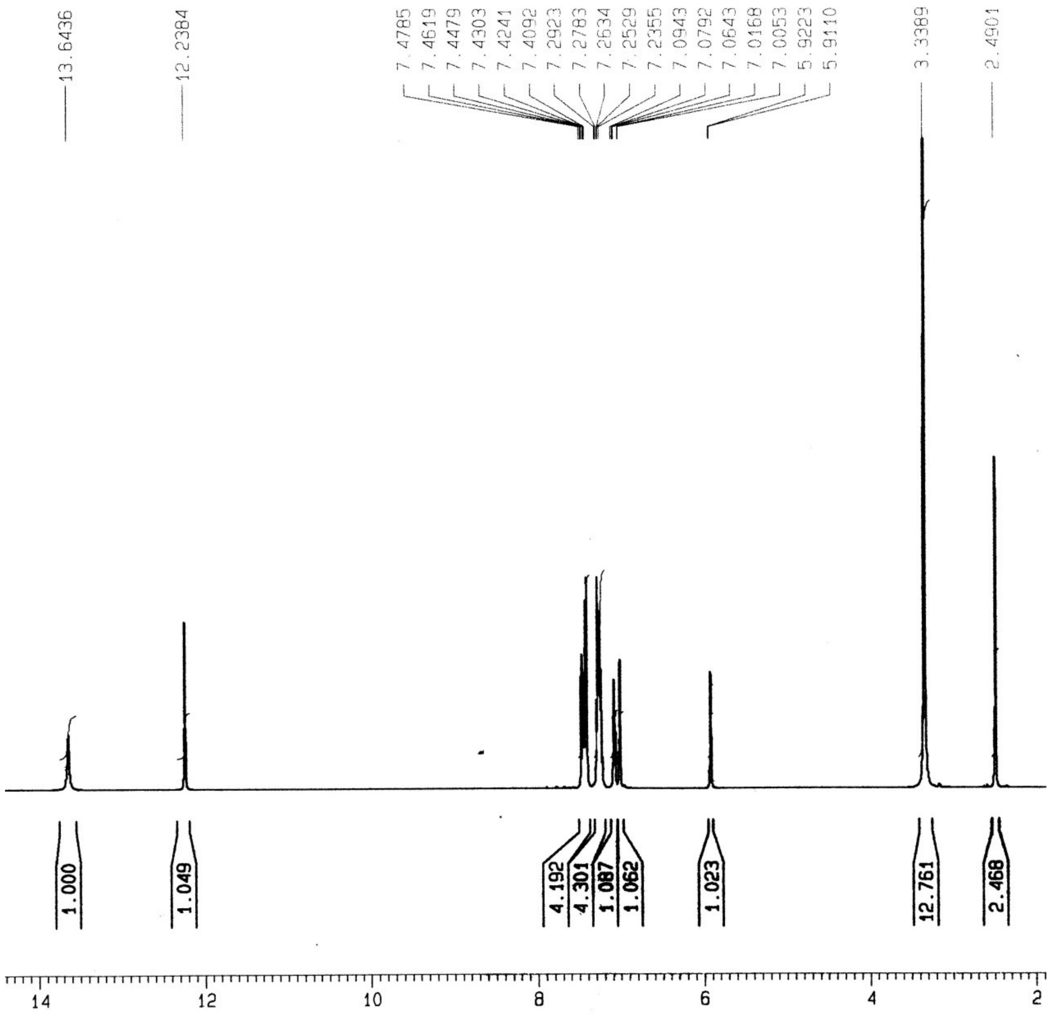

Figure S11. ^{1}H NMR of **7**

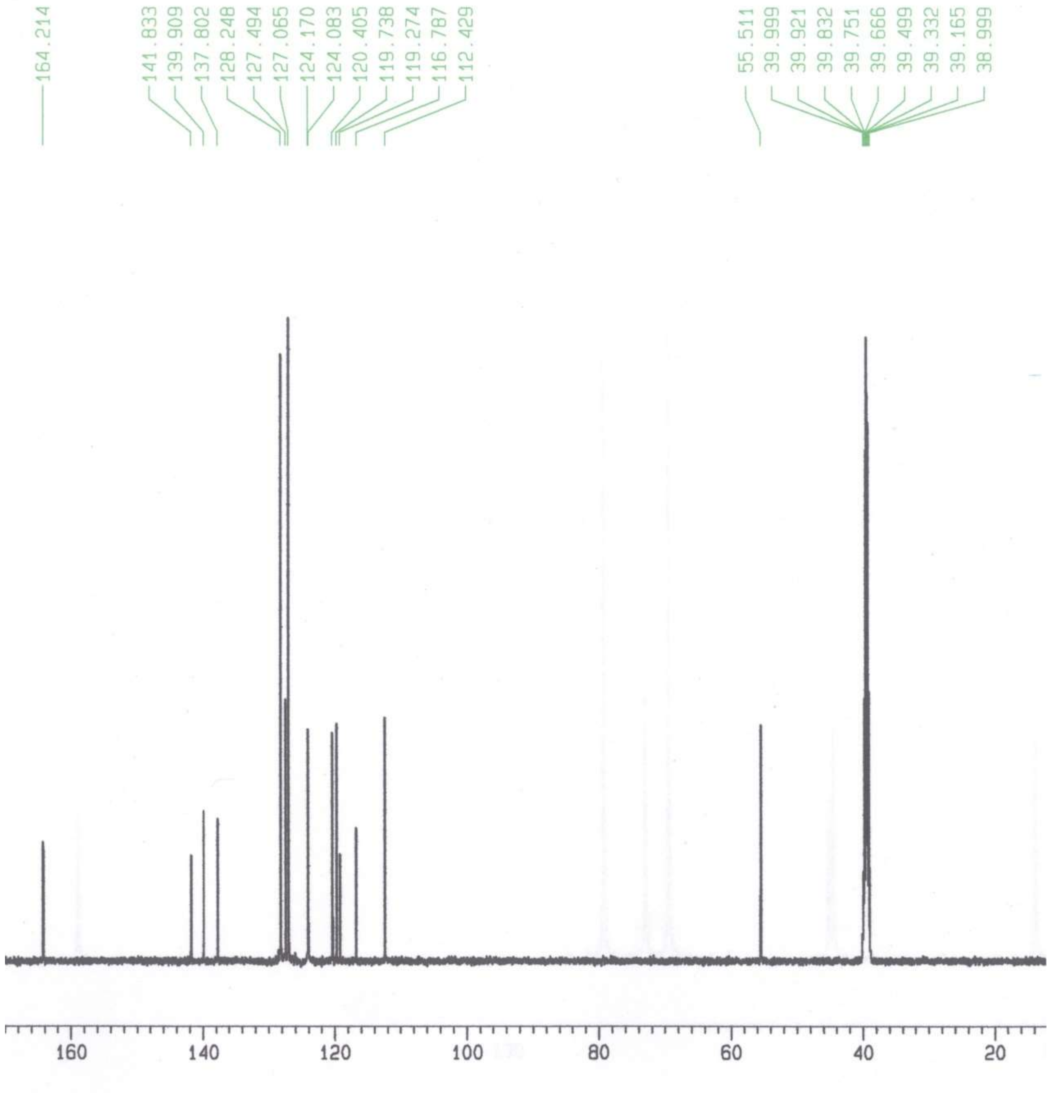

Figure S12. ^{13}C NMR of **7**

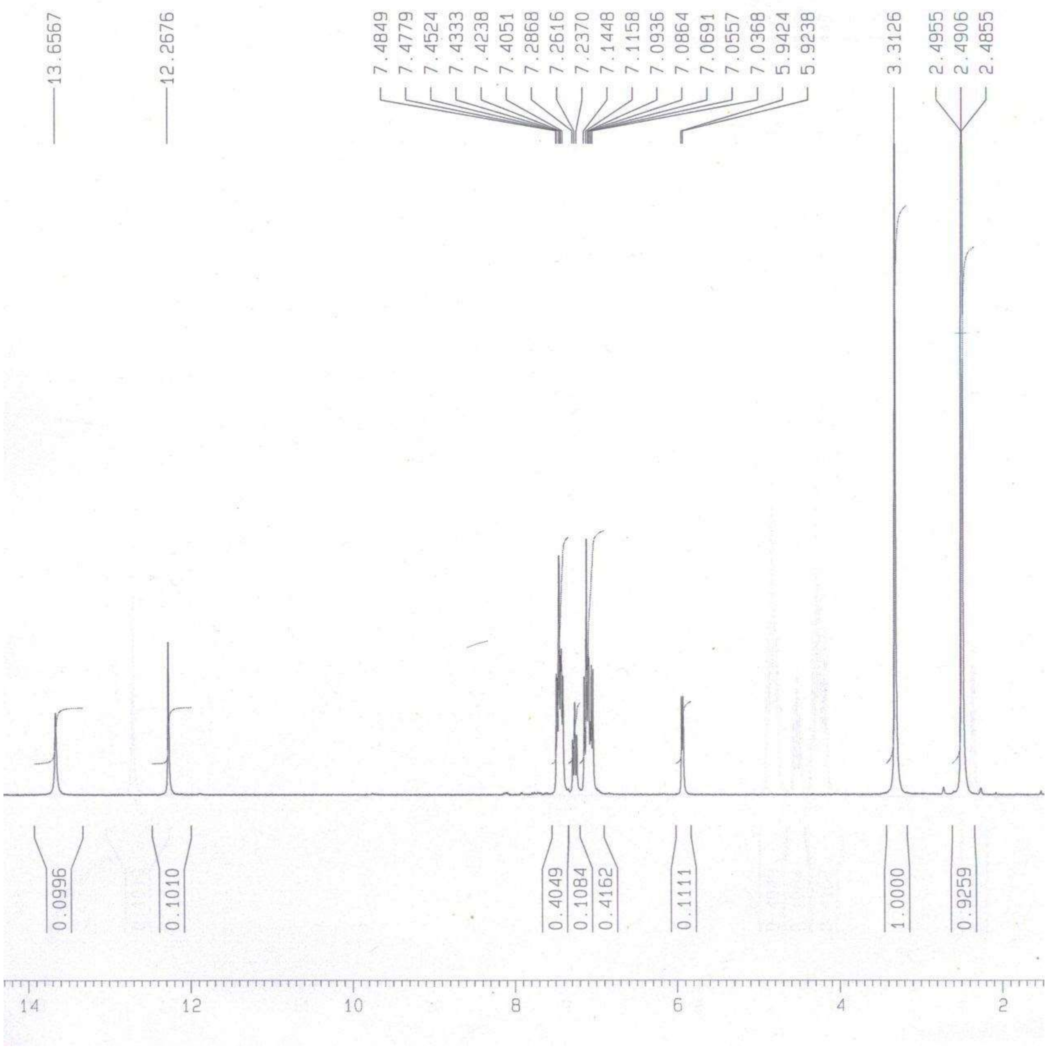

Figure S13. ^{1}H NMR of **8**

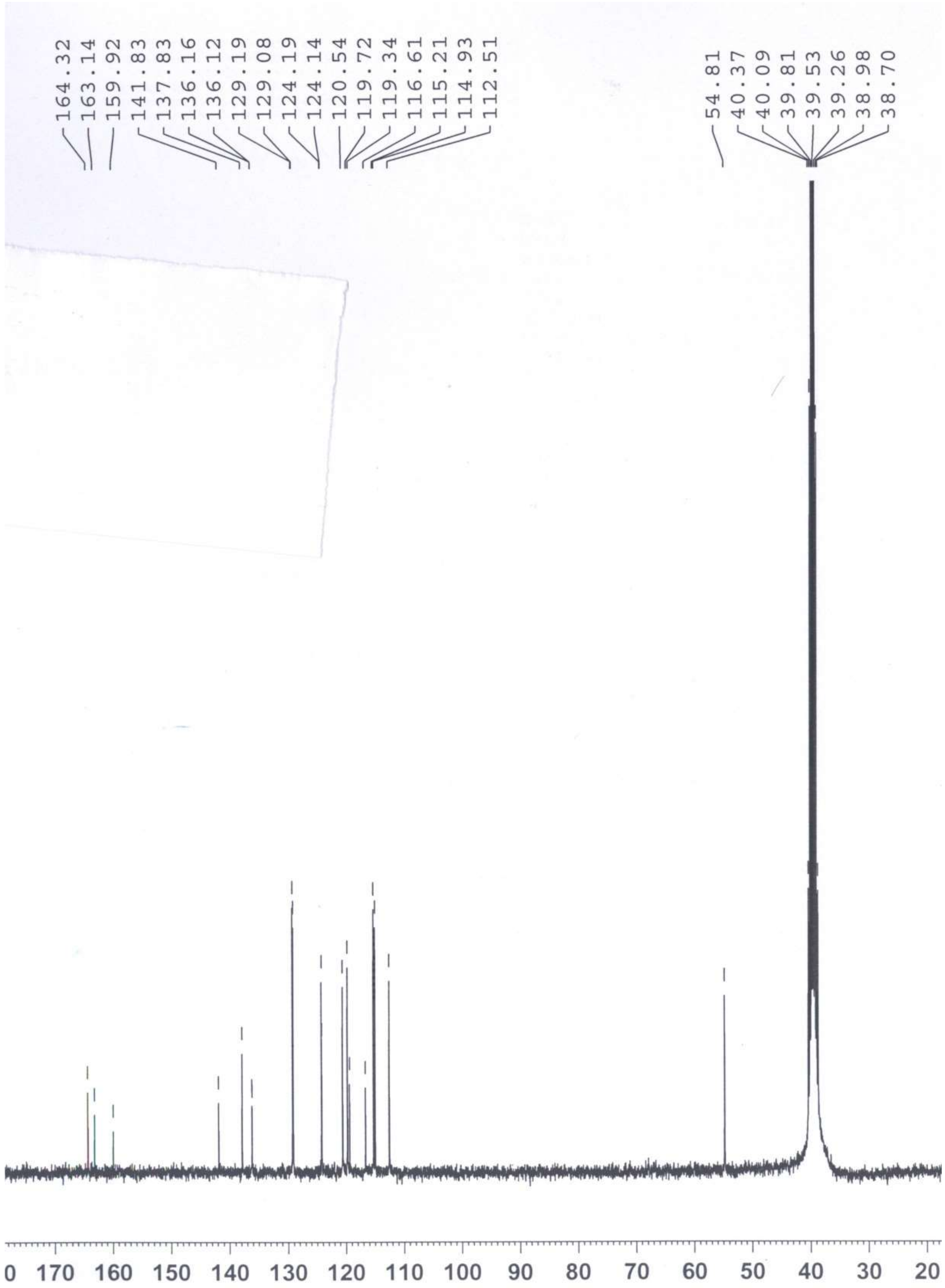

Figure S14. ^{13}C NMR of **8**

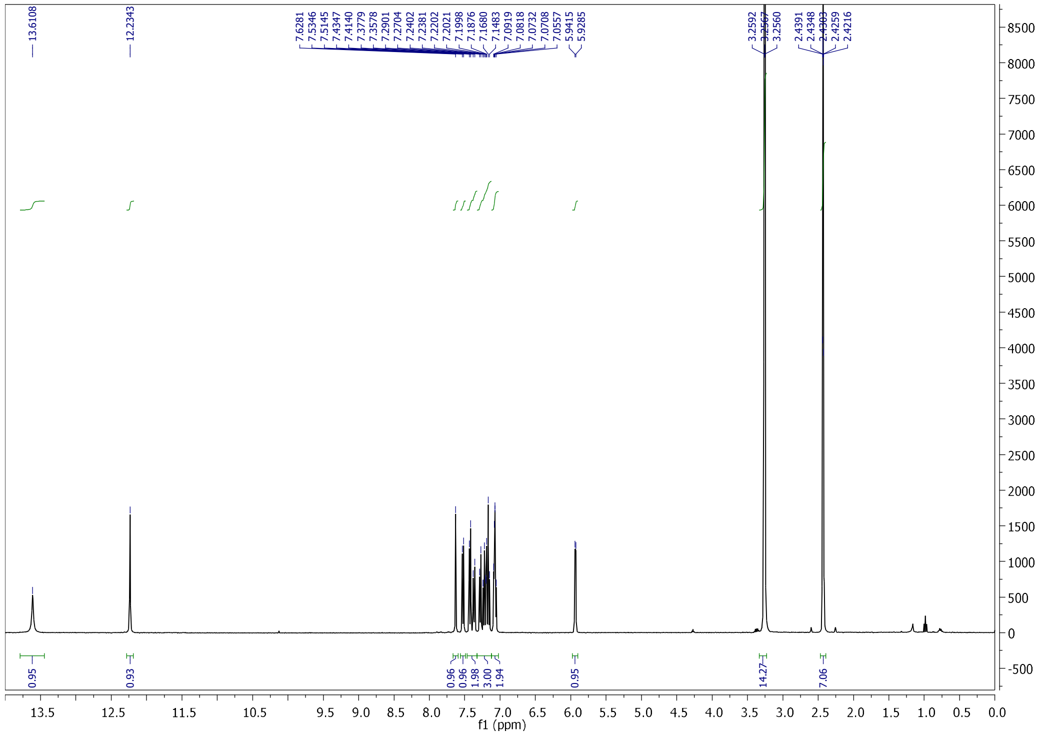

Figure S15. ^{1}H NMR of **9**

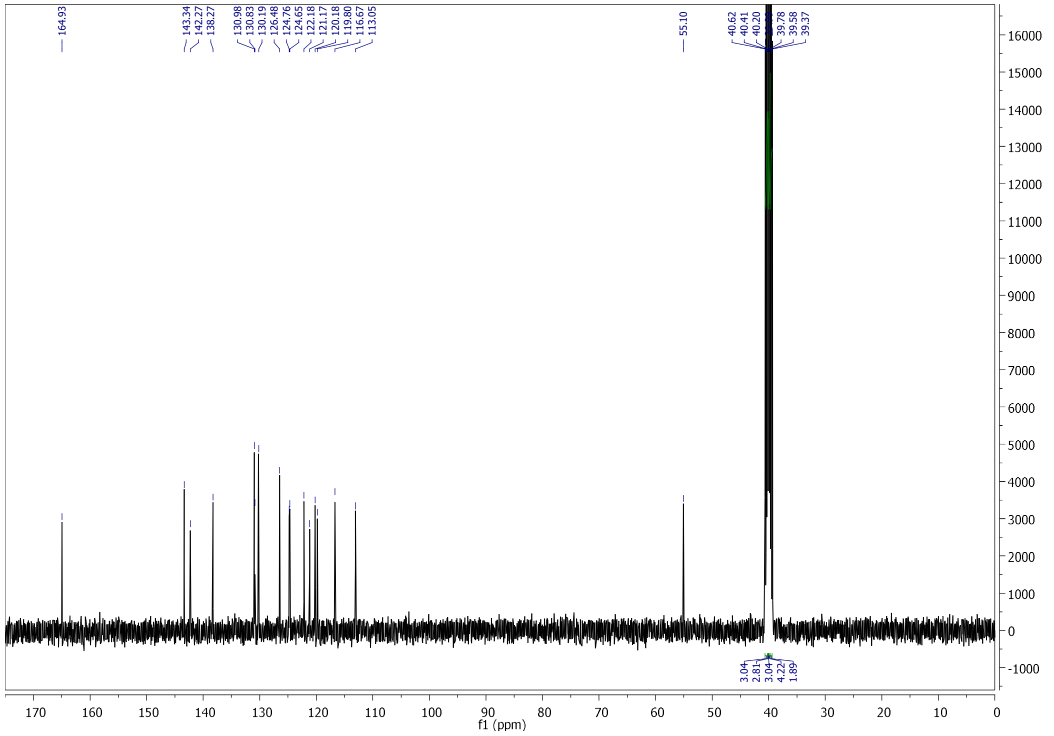

Figure S16. ^{13}C NMR of **9**

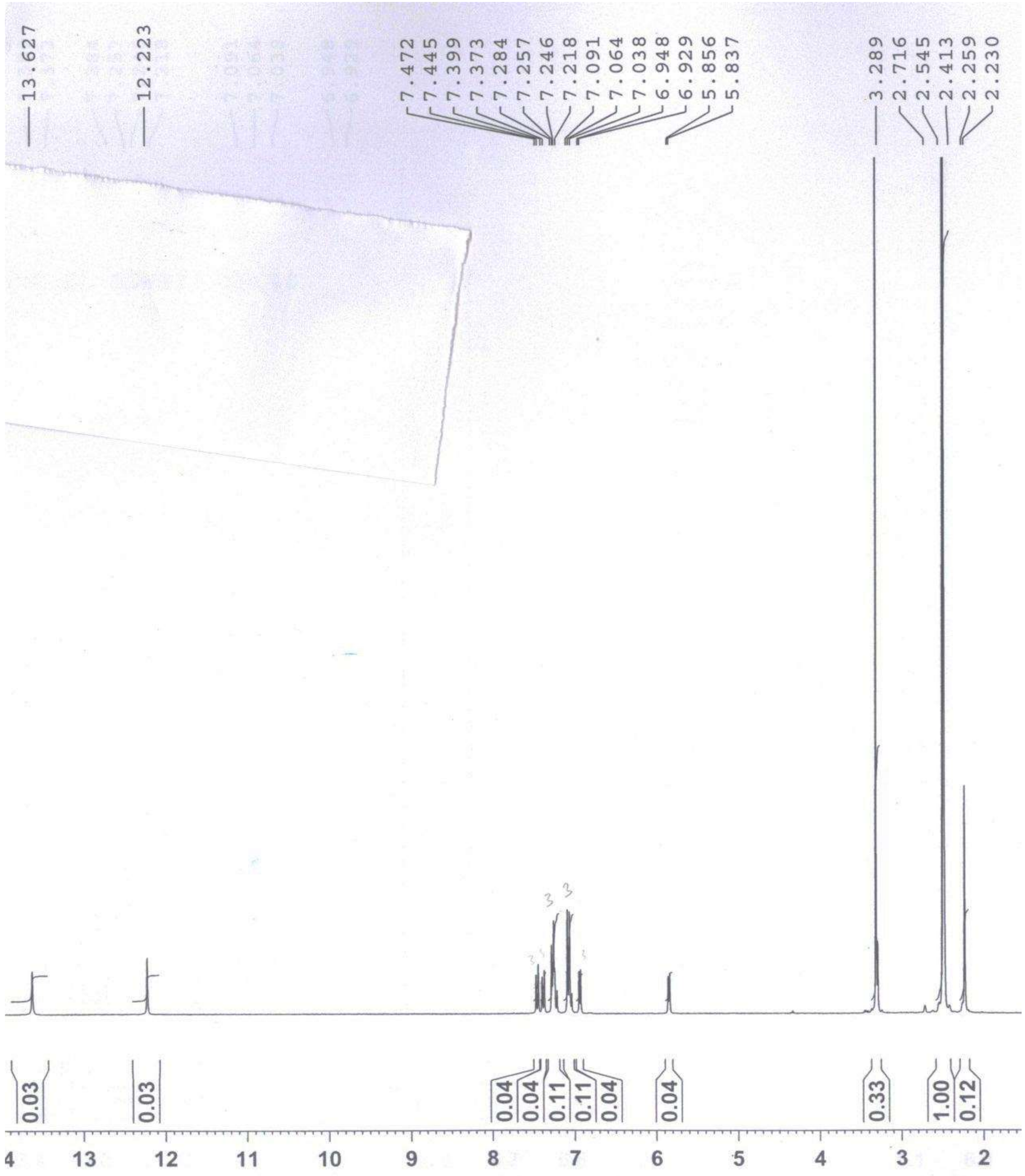

Figure S17. ^{1}H NMR of **10**

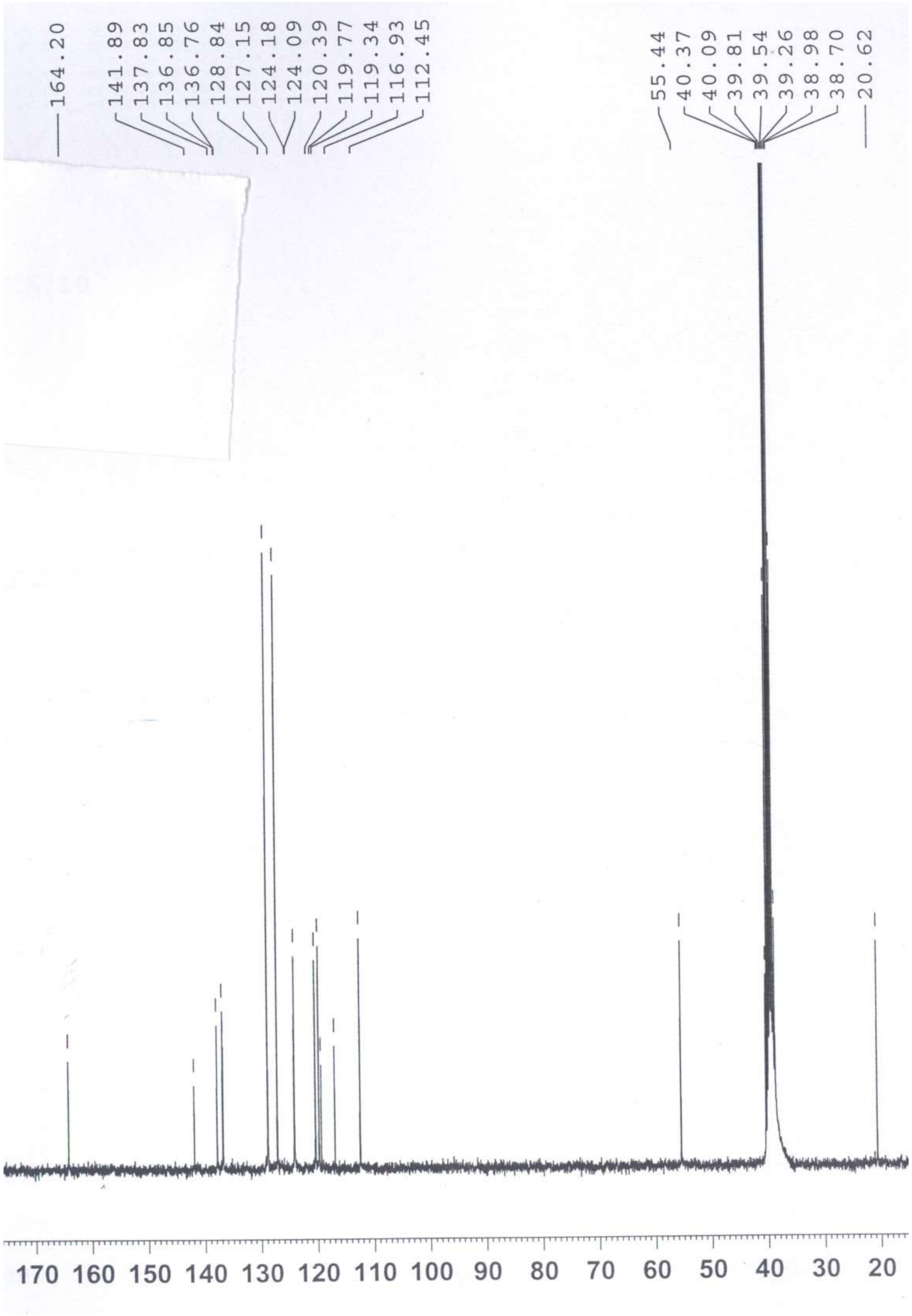

Figure S18. ^{13}C NMR of **10**

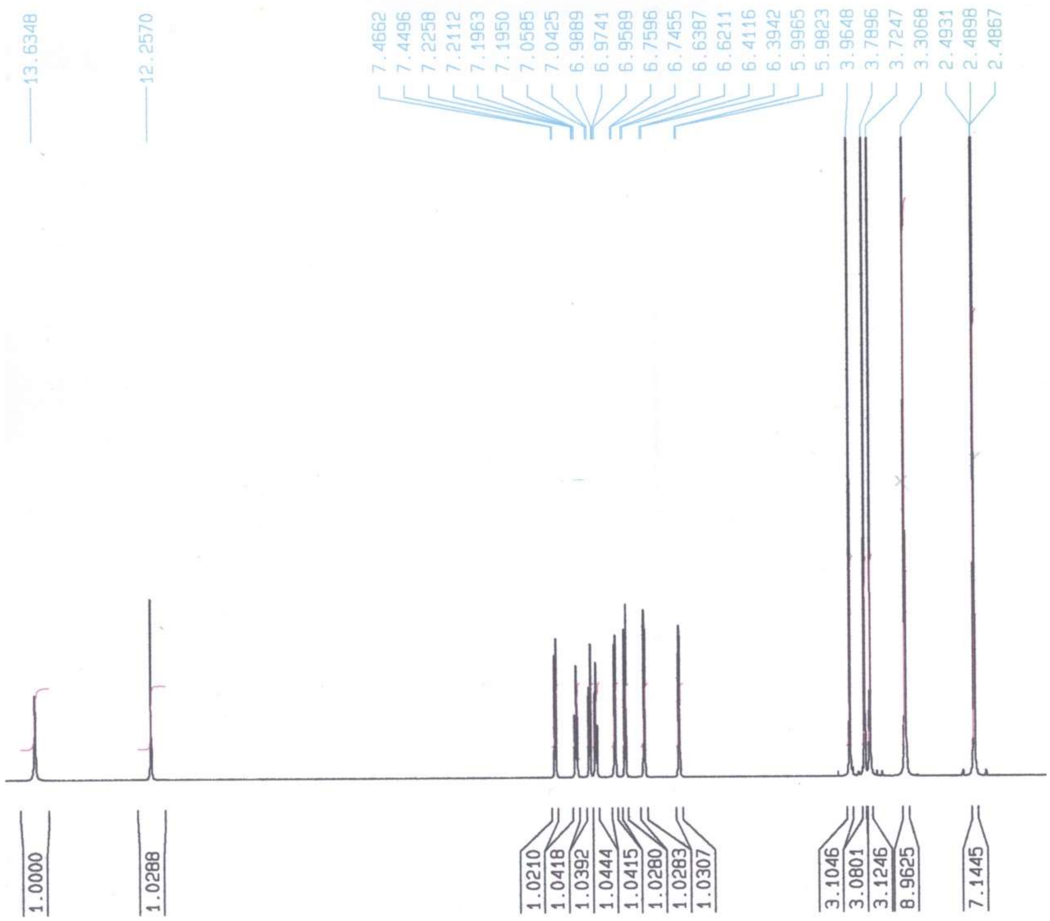

Figure S19. ^{1}H NMR of **11**

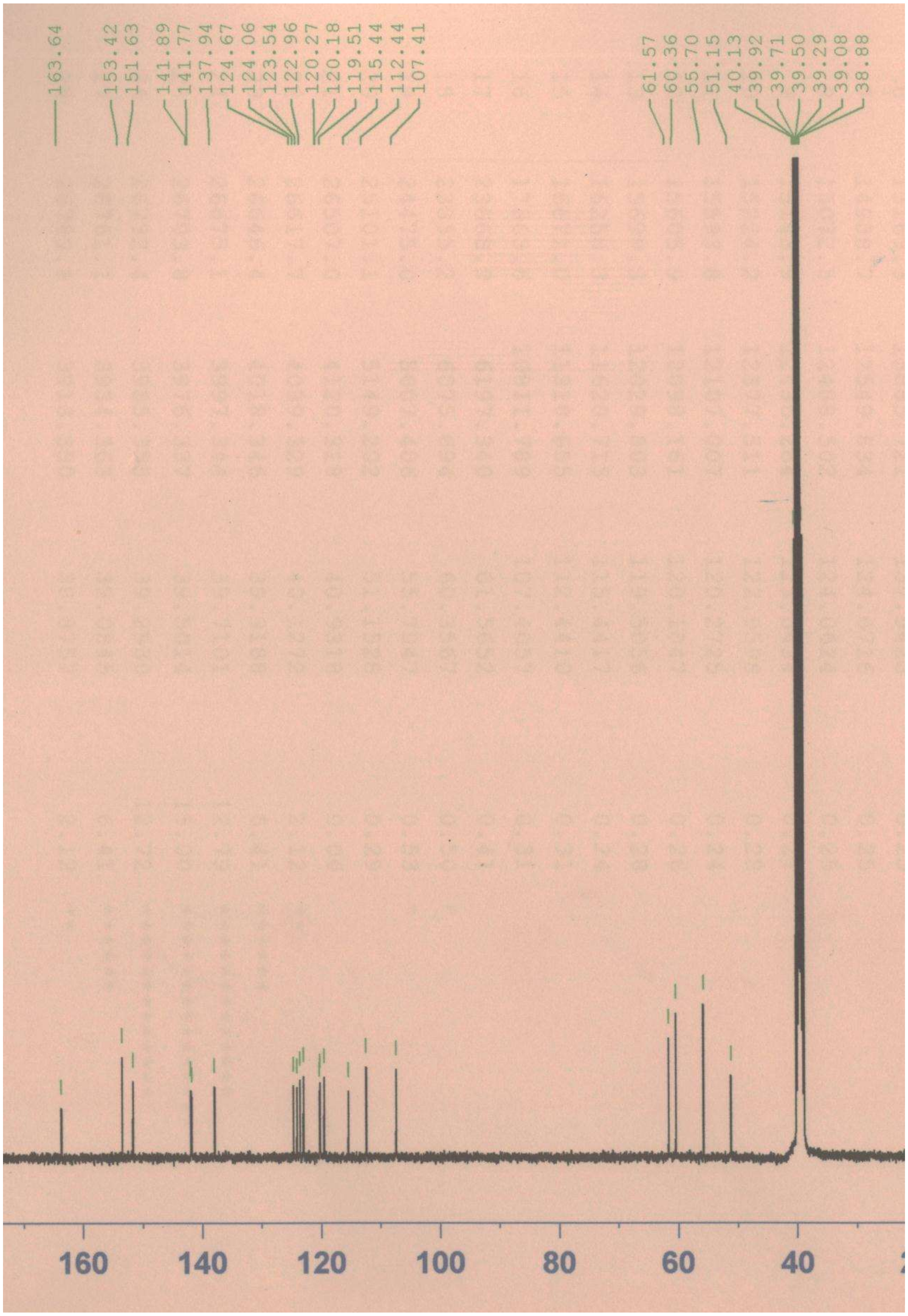

Figure S20. ^{13}C NMR of **11**

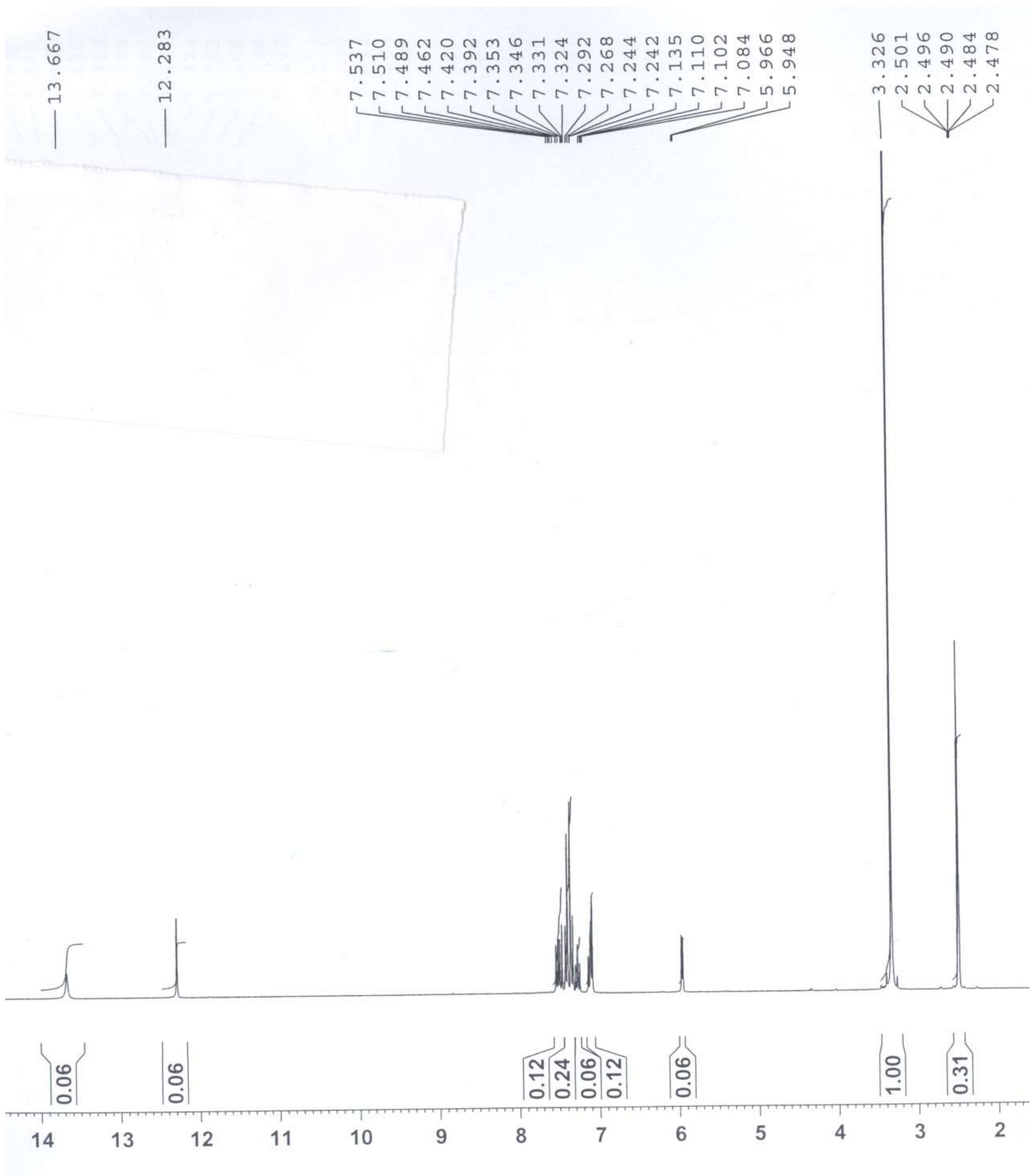

Figure S21. ^{1}H NMR of **12**

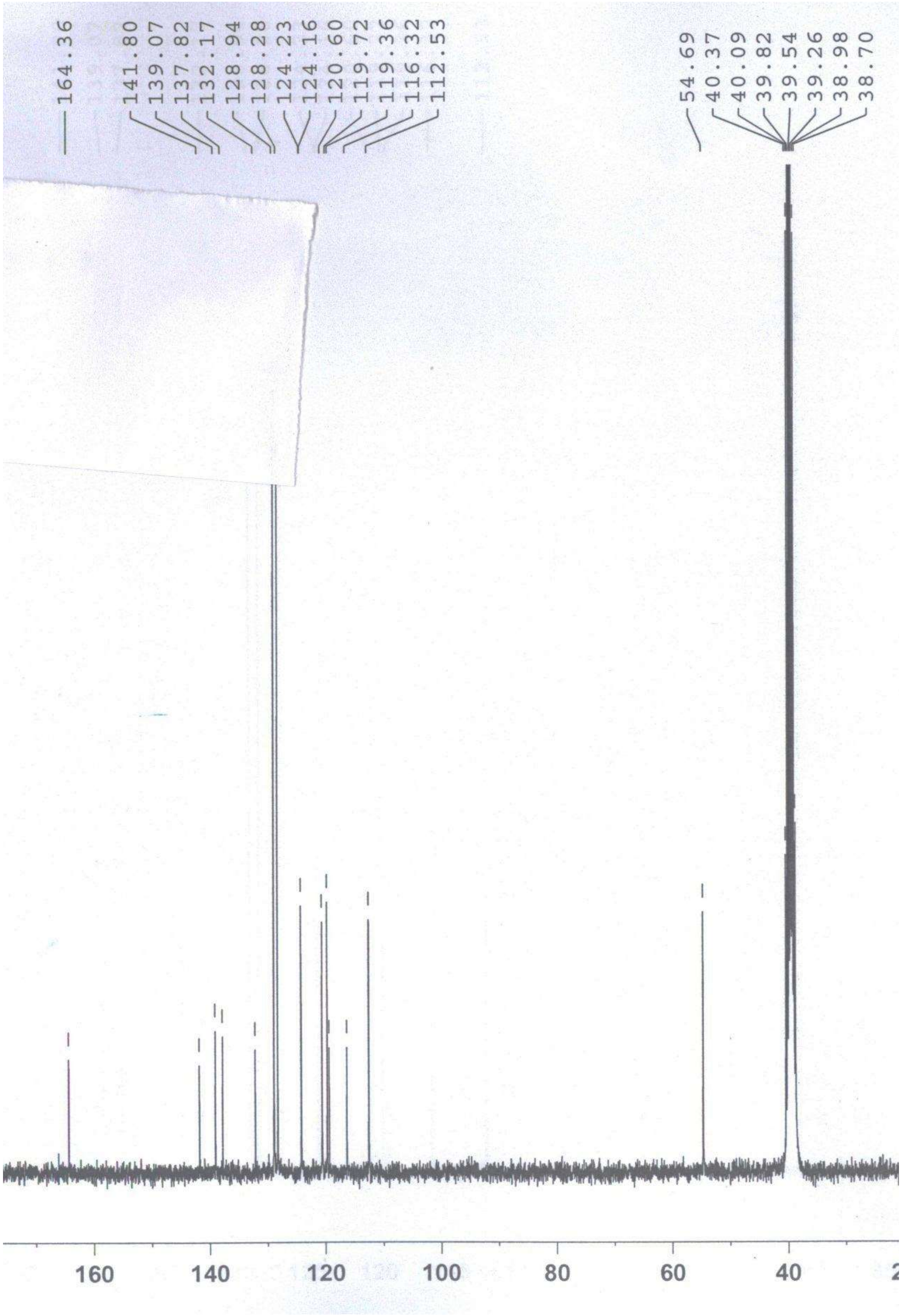

Figure S22. ^{13}C NMR of **12**

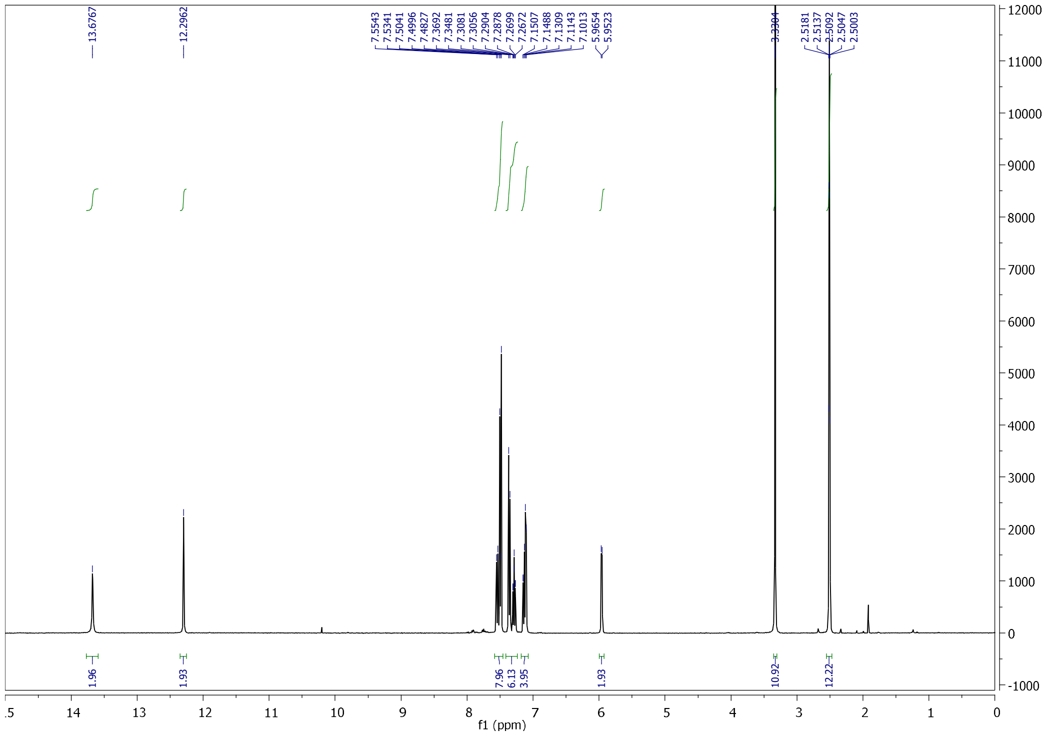

Figure S23. ^{1}H NMR of **13**

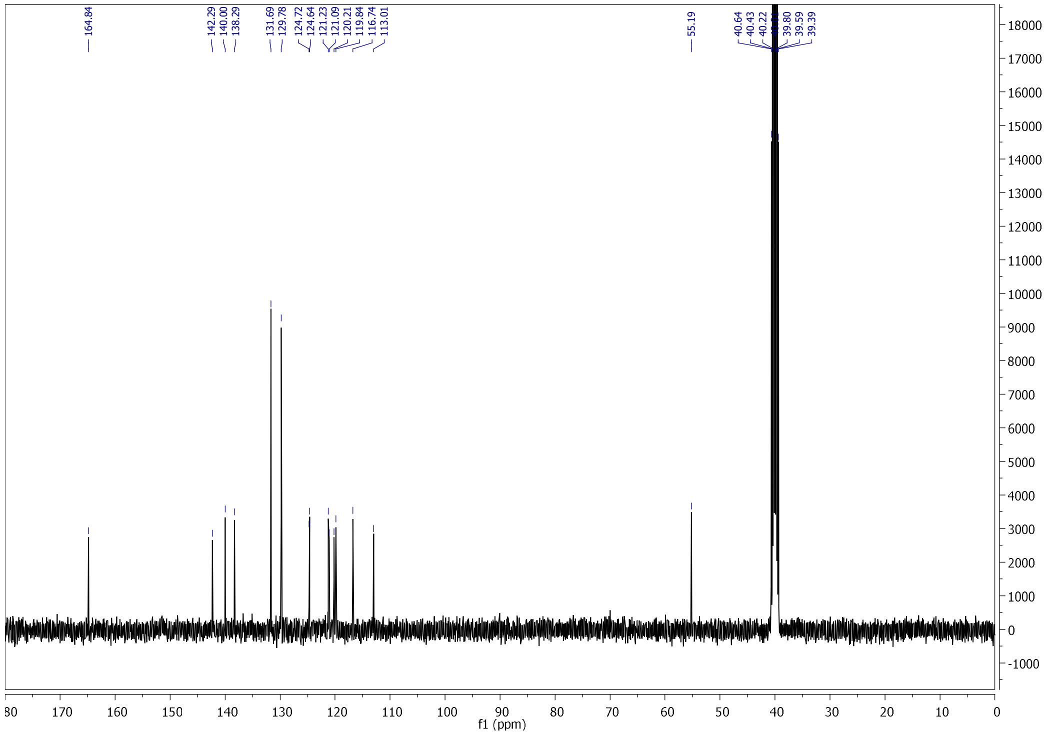

Figure S24. ^{13}C NMR of **13**

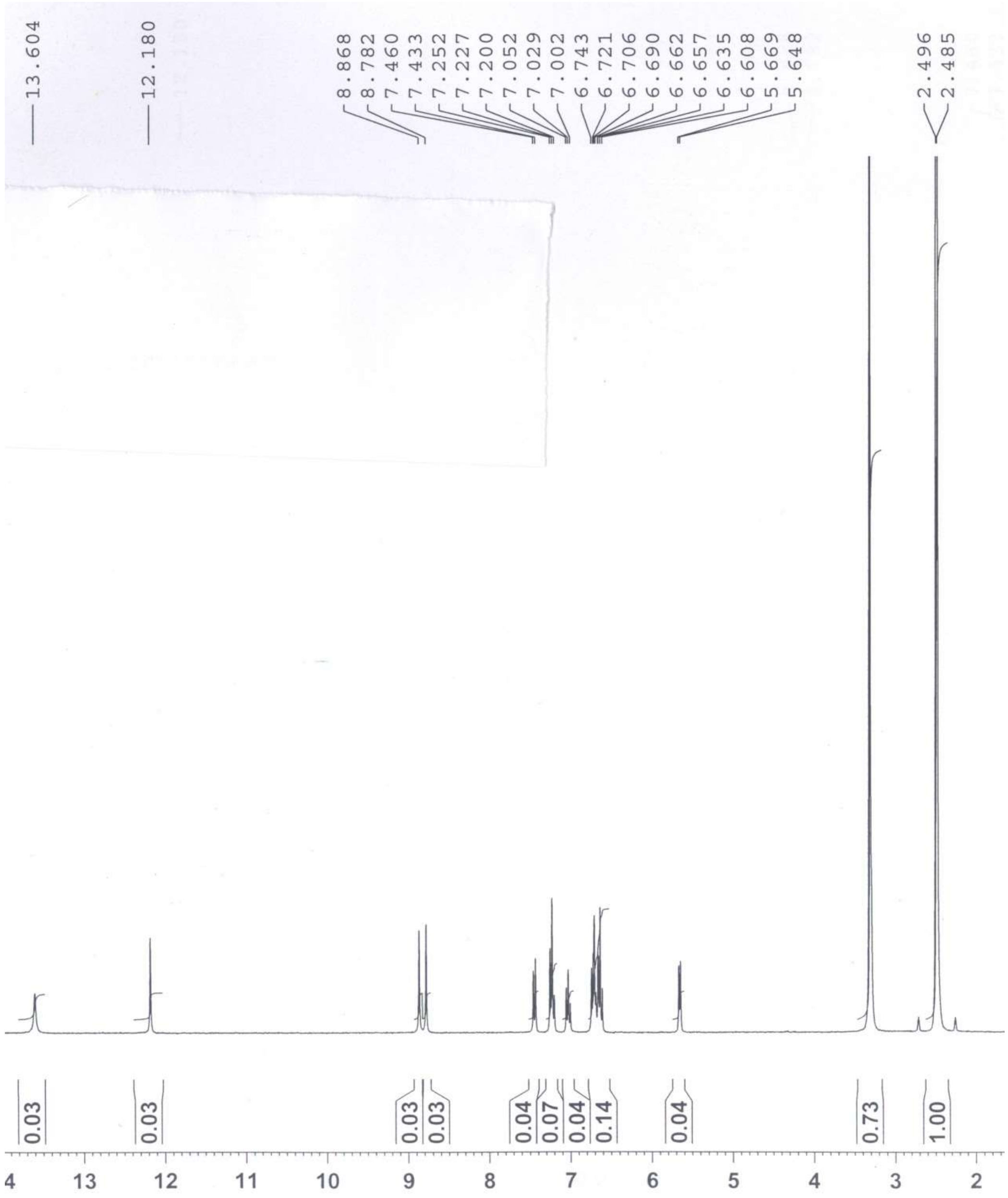

Figure S25. ^{1}H NMR of **14**

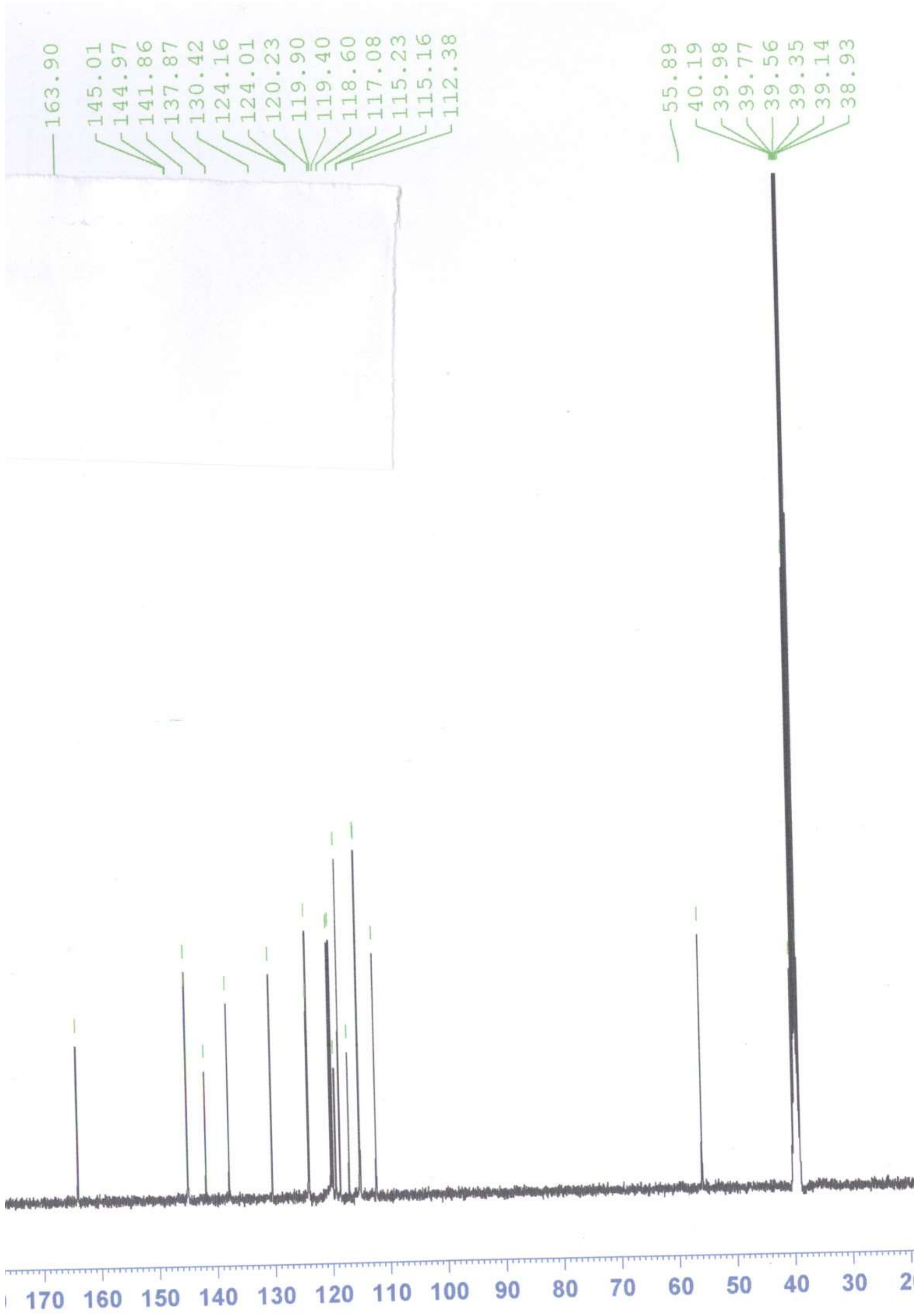

Figure S26. ^{13}C NMR of **14**

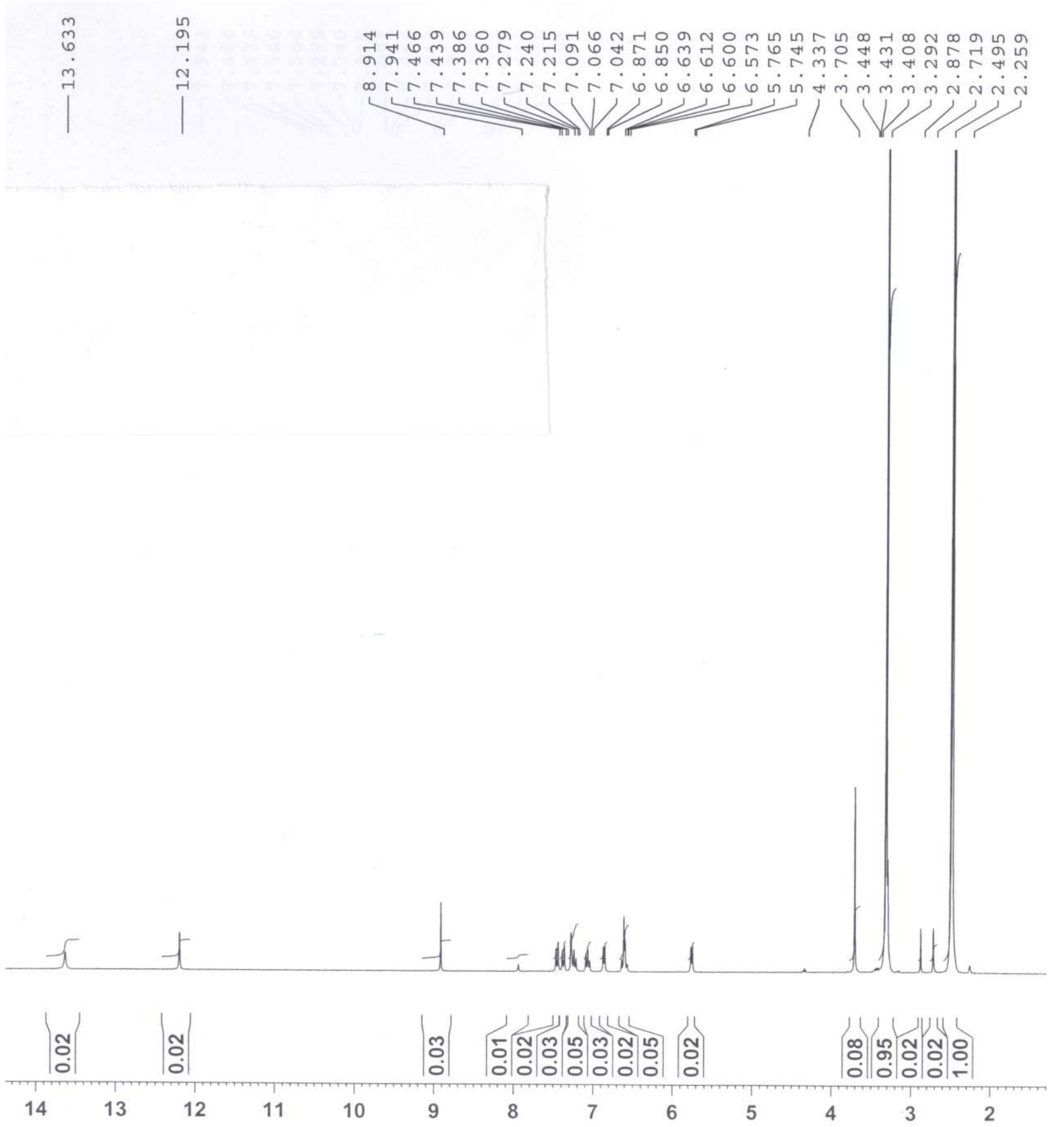

Figure S27. ^{1}H NMR of **15**

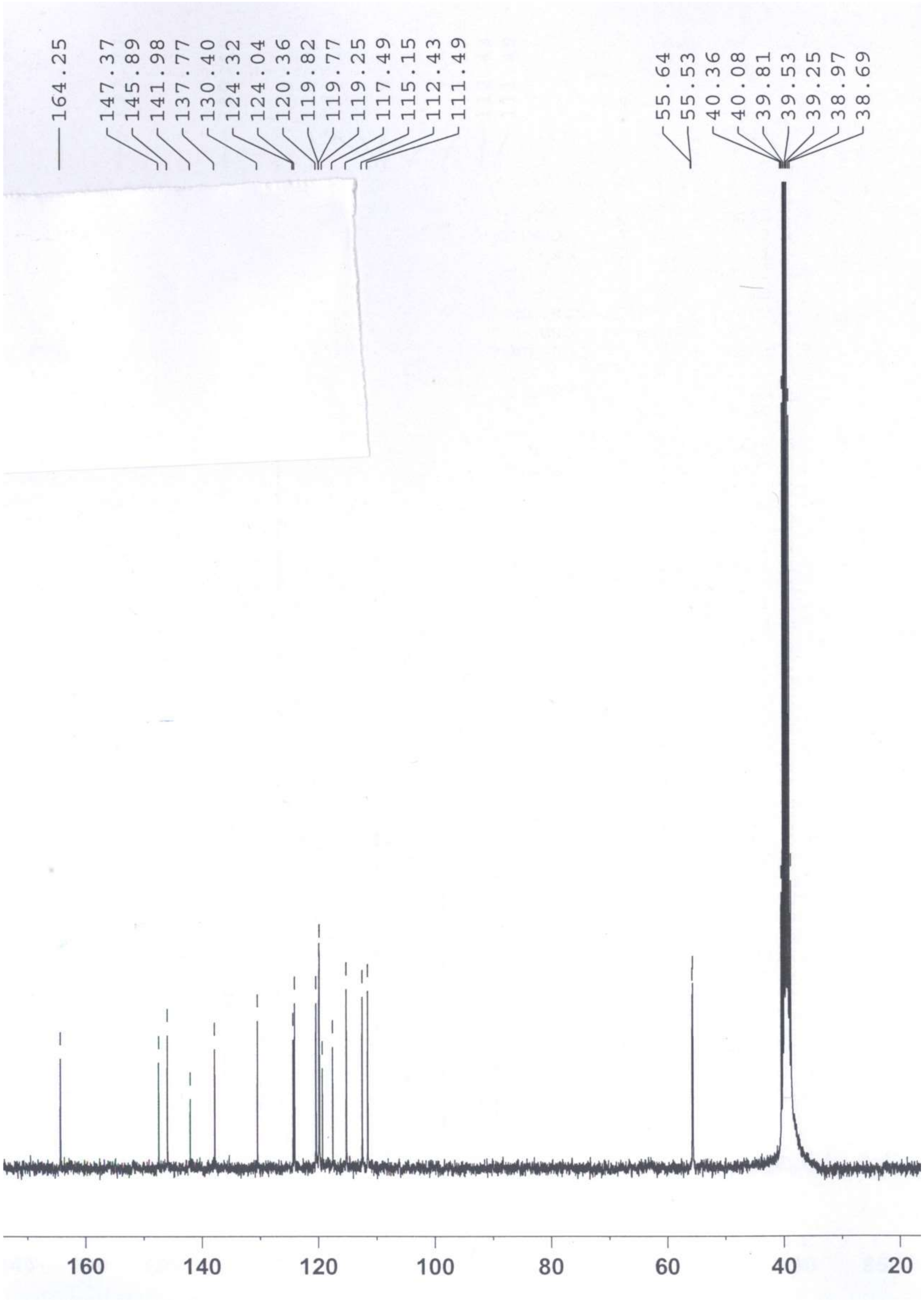

Figure S28. ^{13}C NMR of **15**

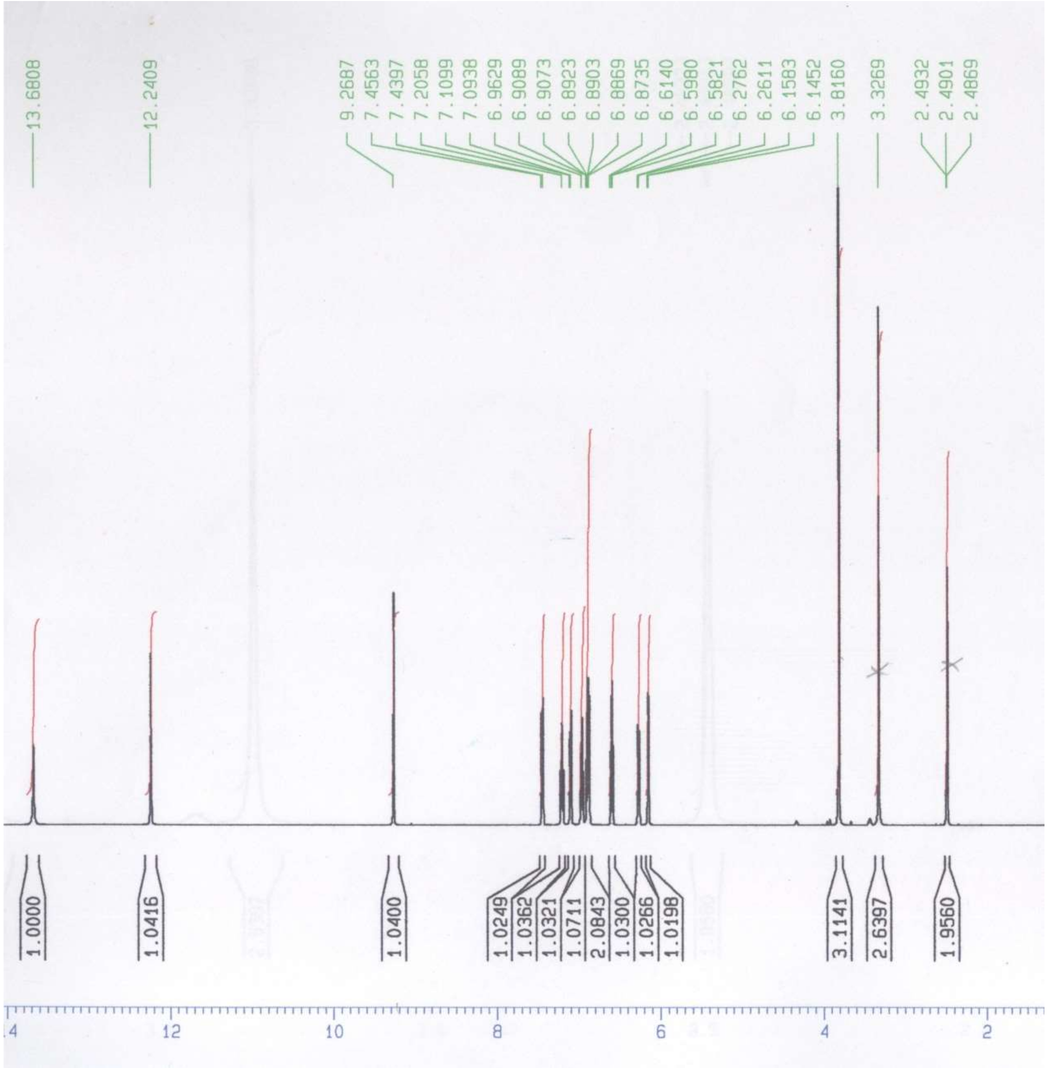

Figure S29. ^{1}H NMR of **16**

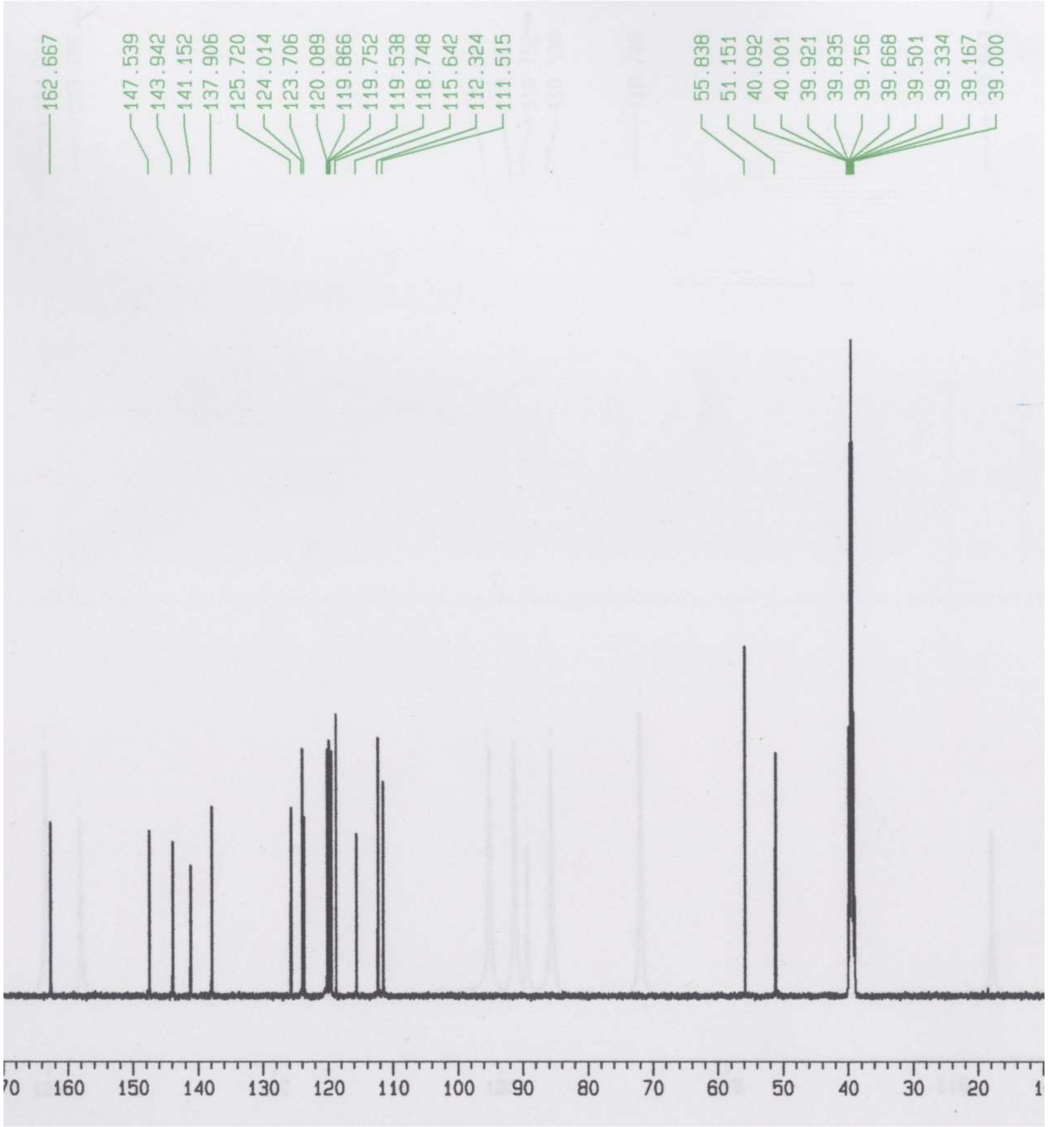

Figure S30. ^{13}C NMR of **16**

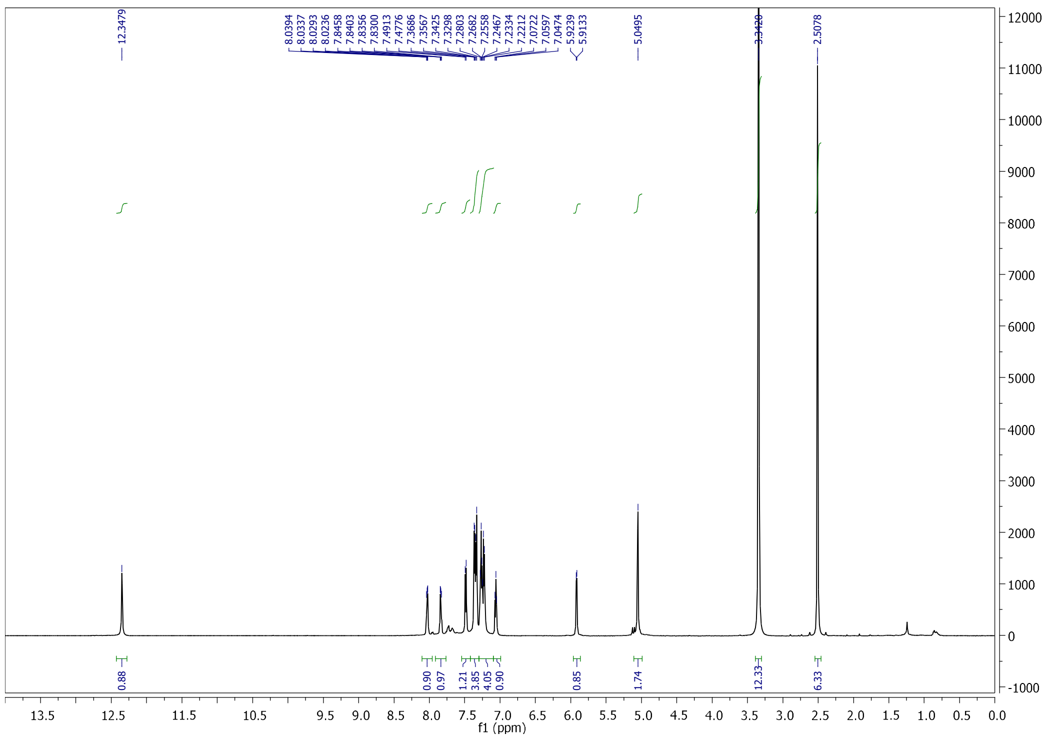

Figure S31. ^{1}H NMR of **19**

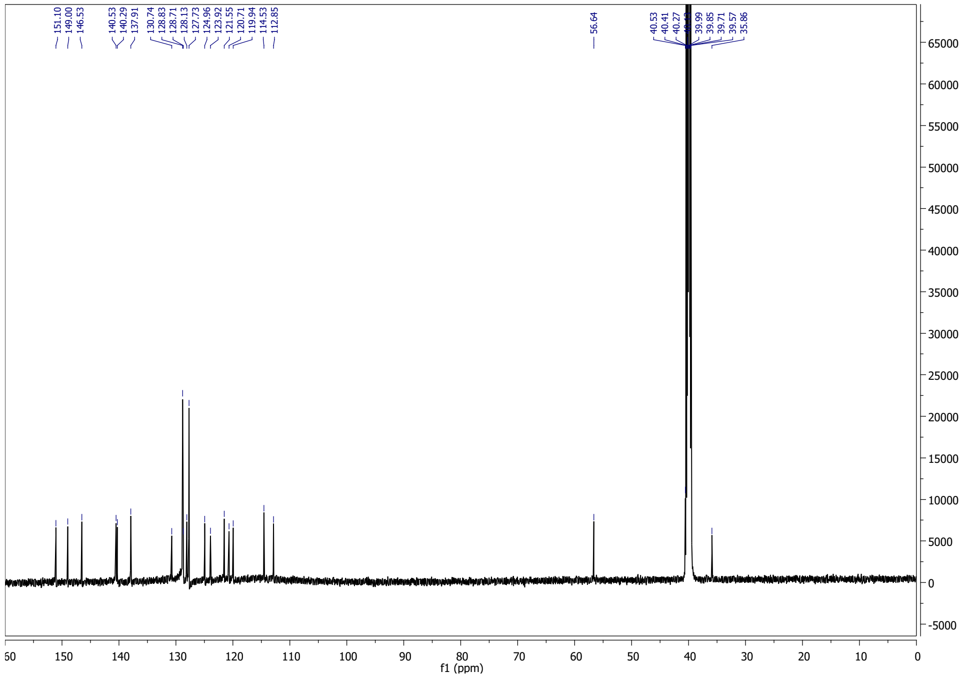

Figure S32. ^{13}C NMR of **19**

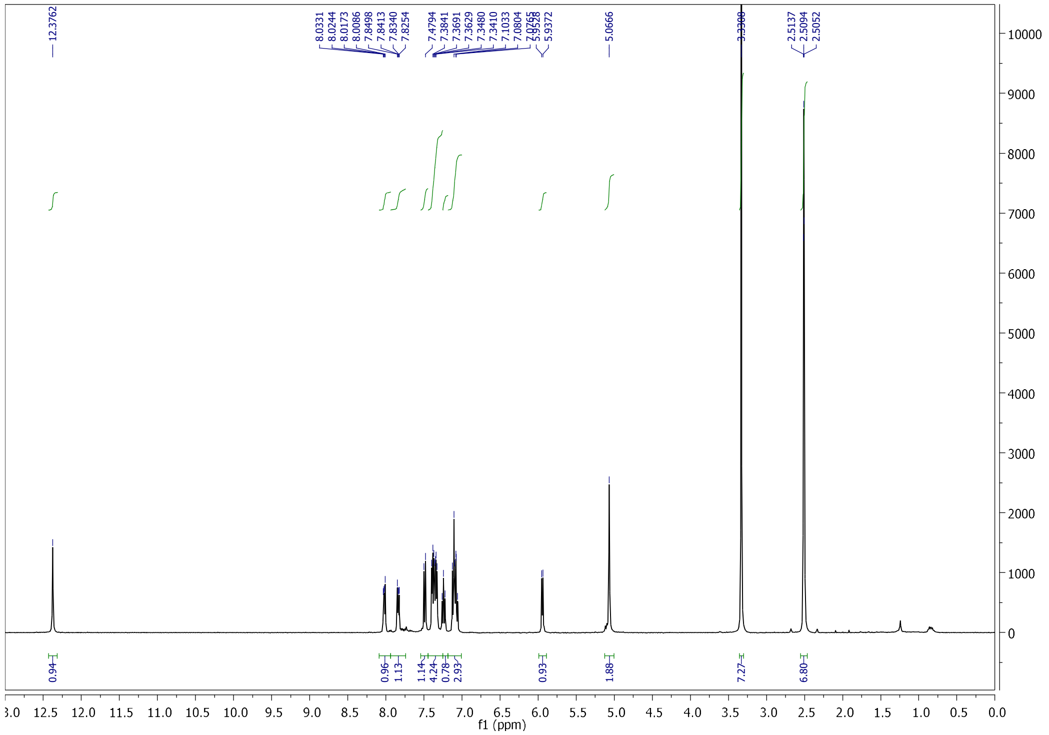

Figure S33. ^{1}H NMR of **20**

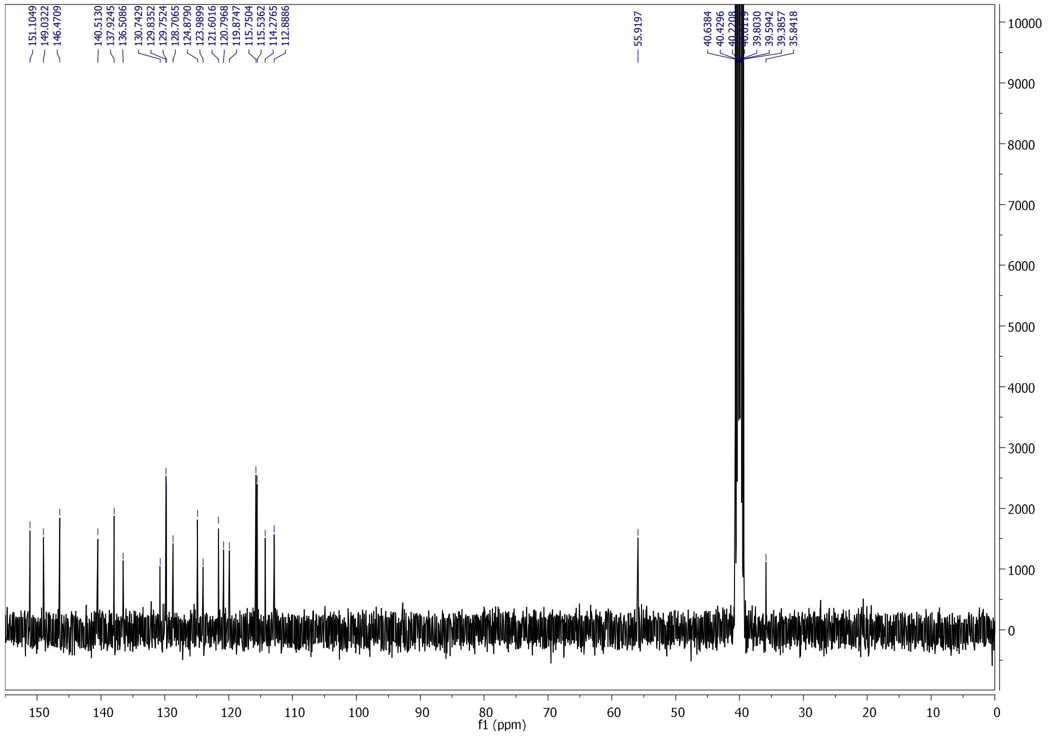

Figure S34. ^{13}C NMR of **20**

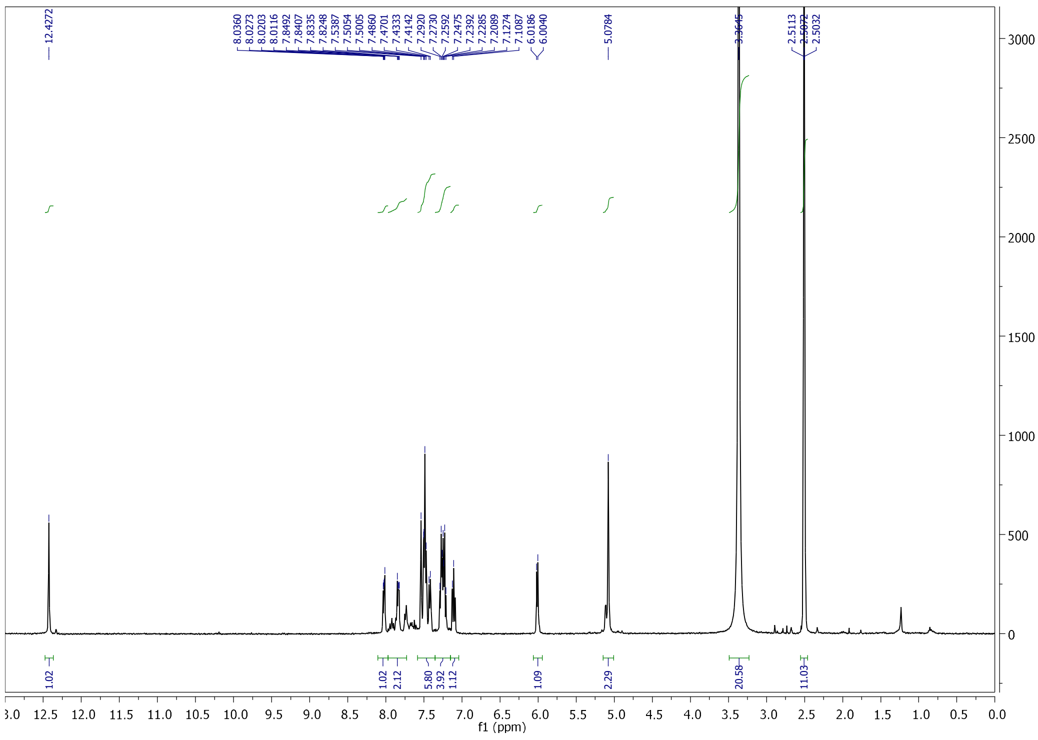

Figure S35. ^{1}H NMR of **21**

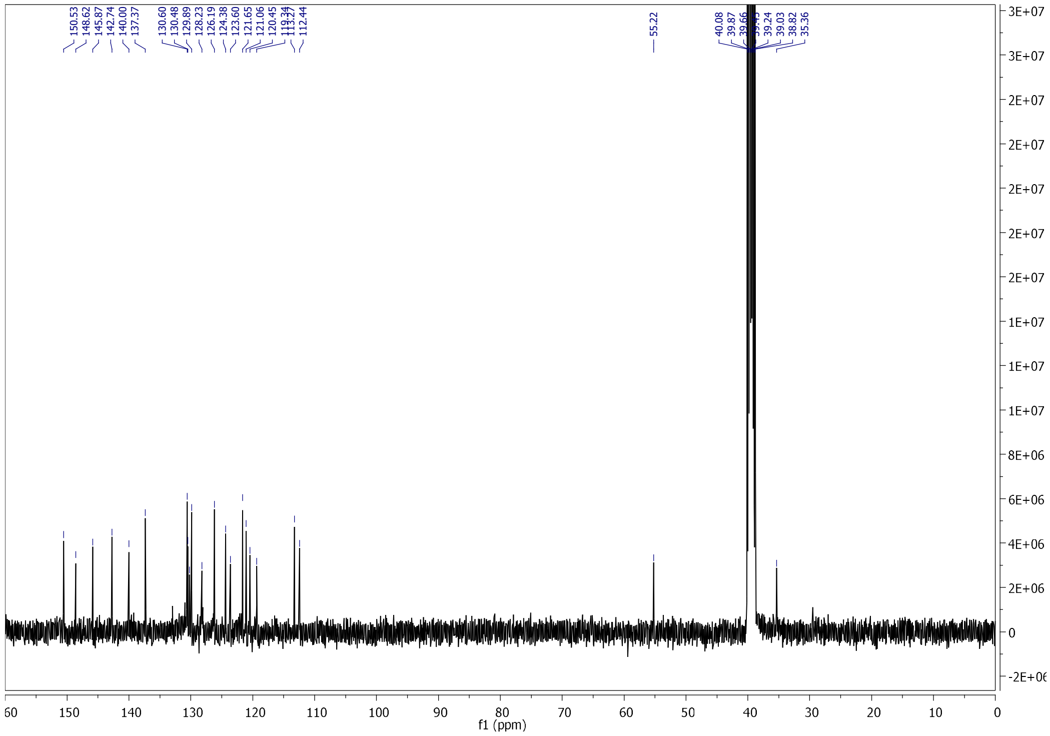

Figure S36. ^{13}C NMR of **21**

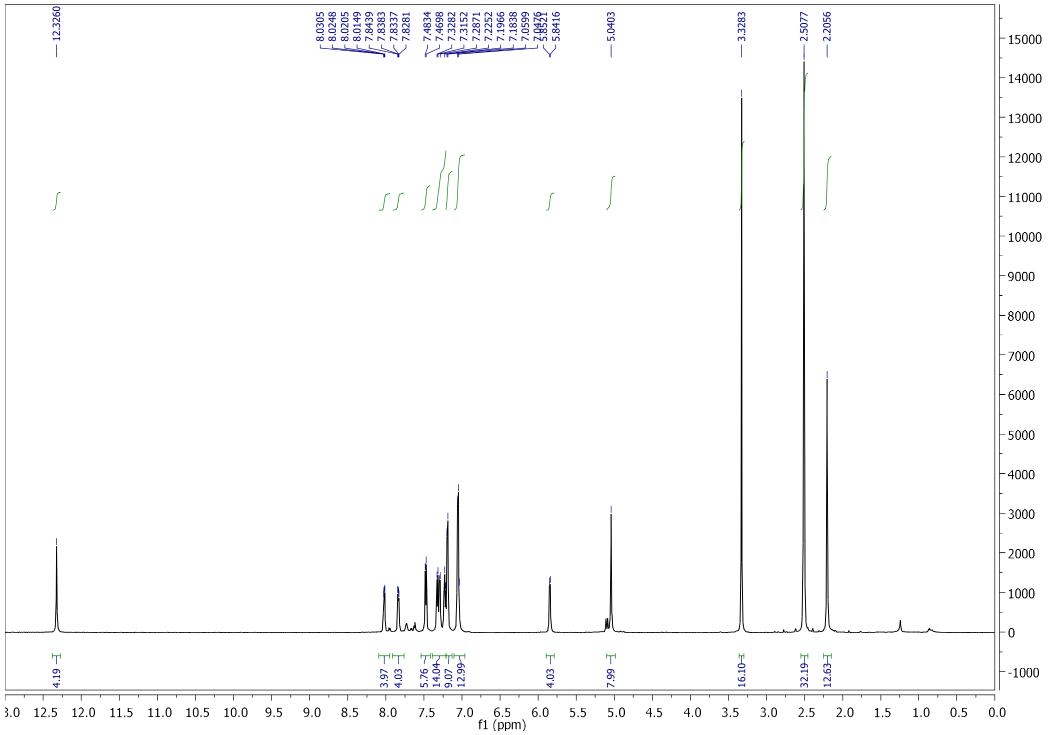

Figure S37. ^{1}H NMR of **22**

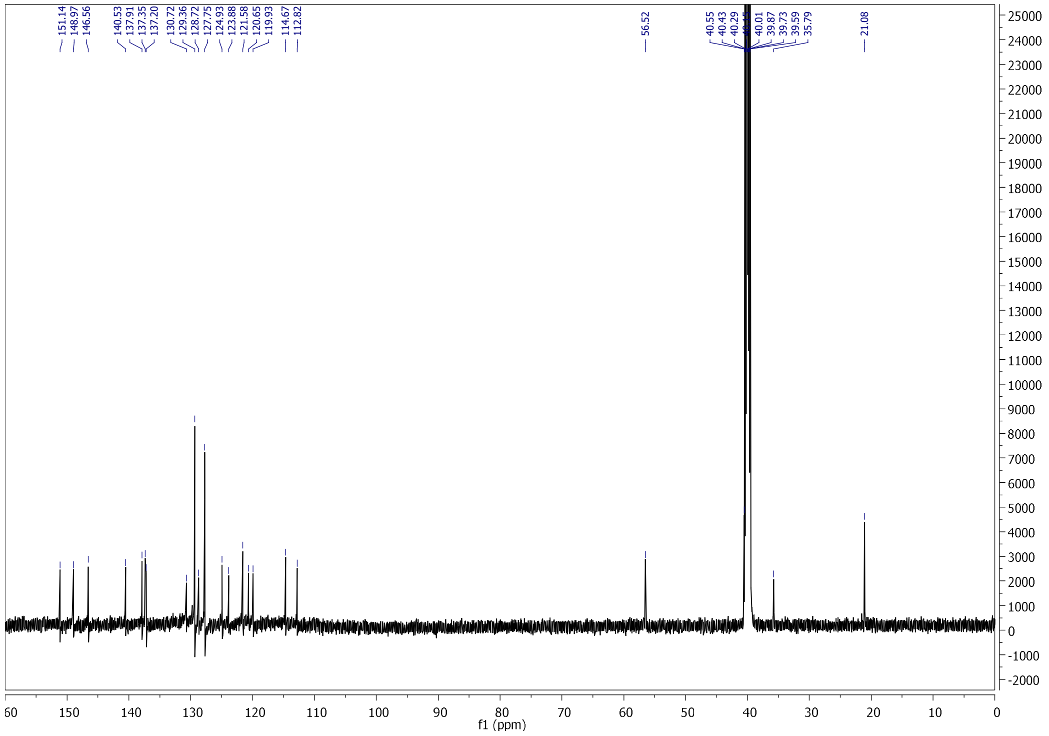

Figure S38. ^{13}C NMR of **22**

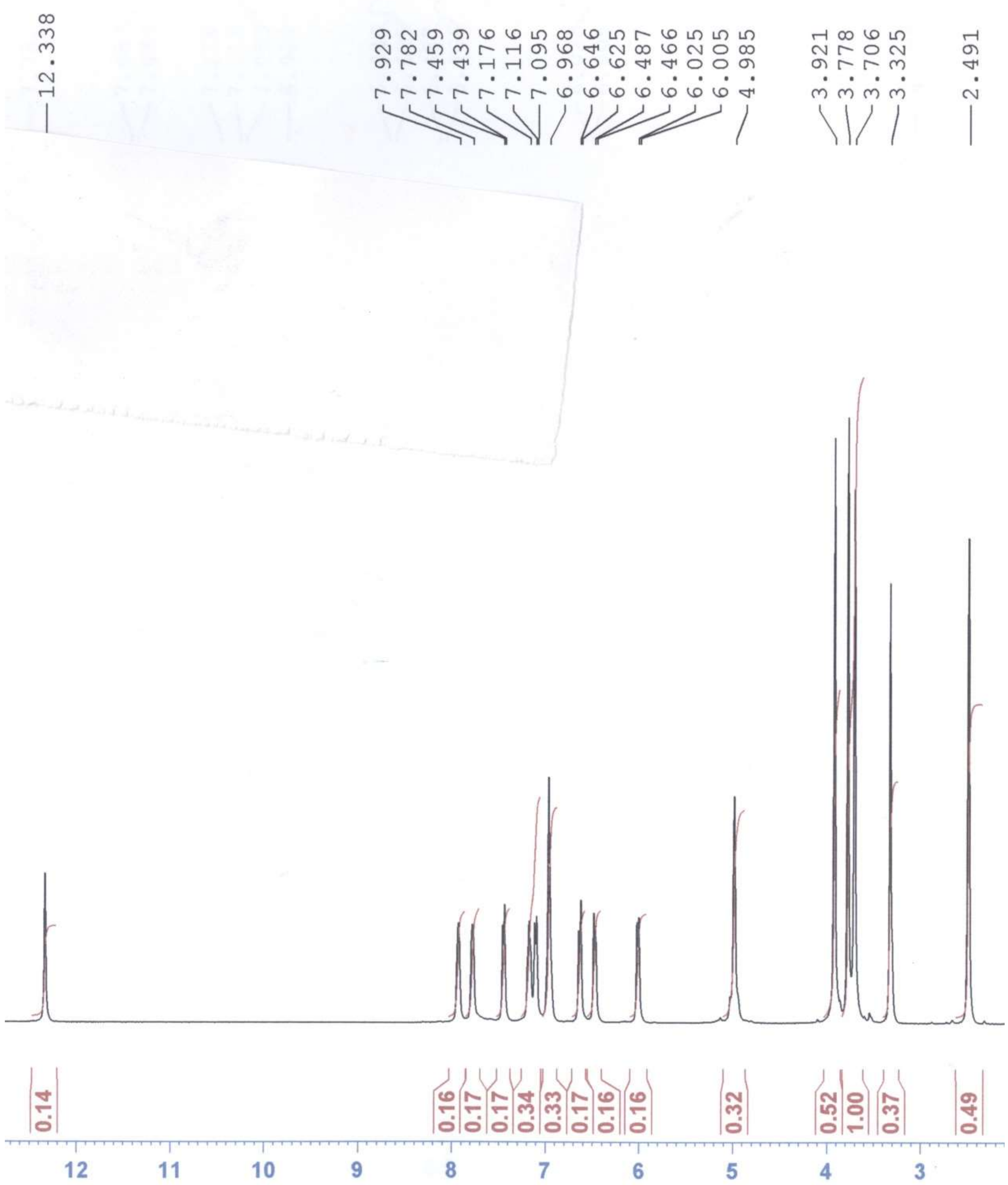

Figure S39. ^{1}H NMR of **23**

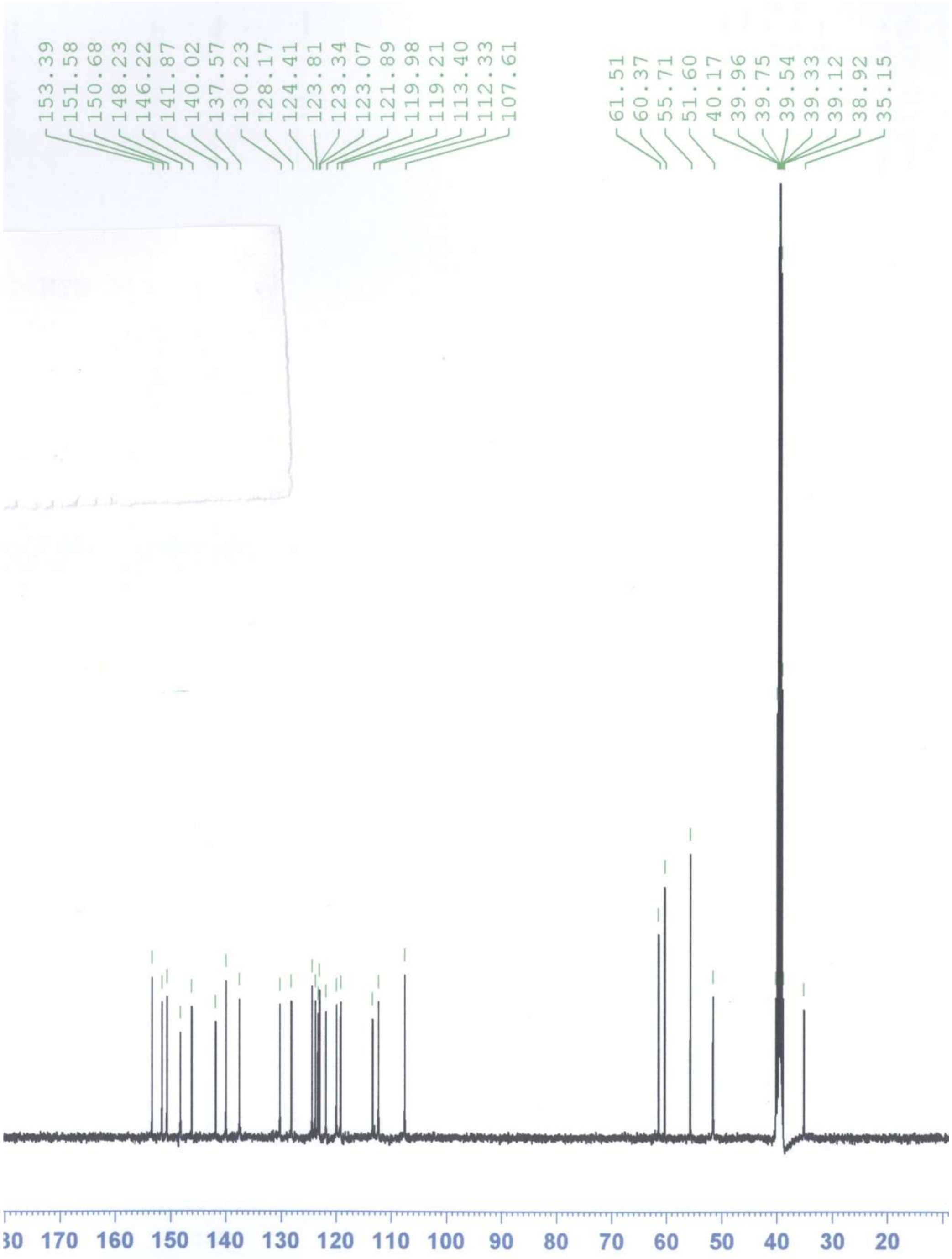

Figure S40. ^{13}C NMR of **23**

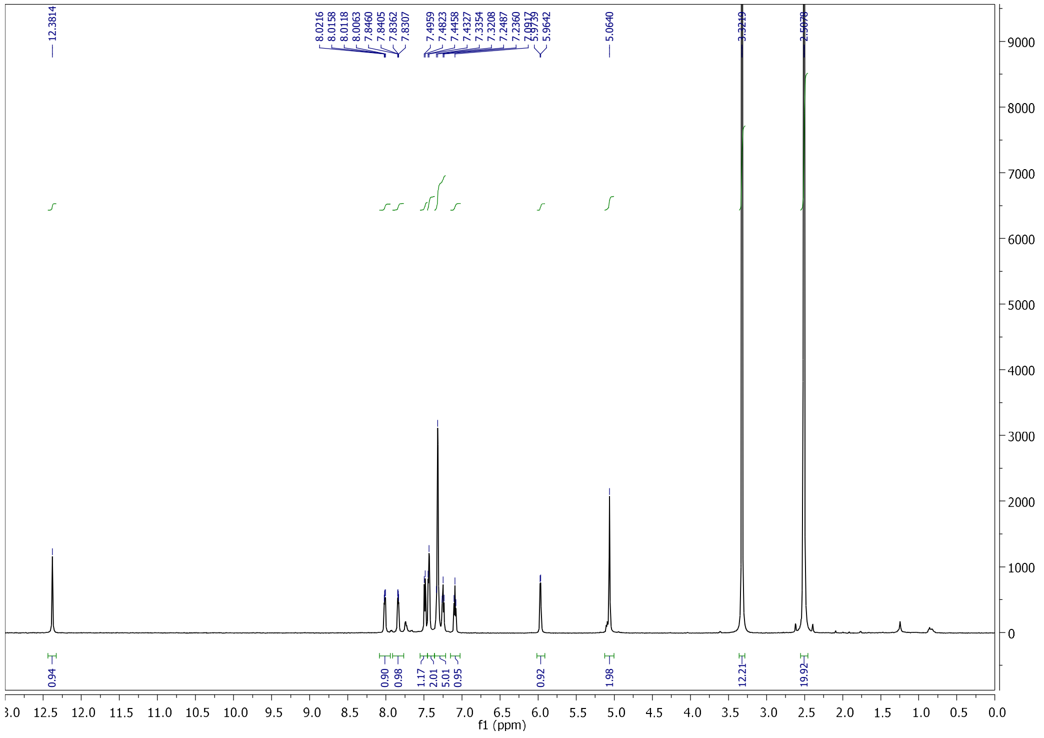

Figure S41. ^{1}H NMR of **24**

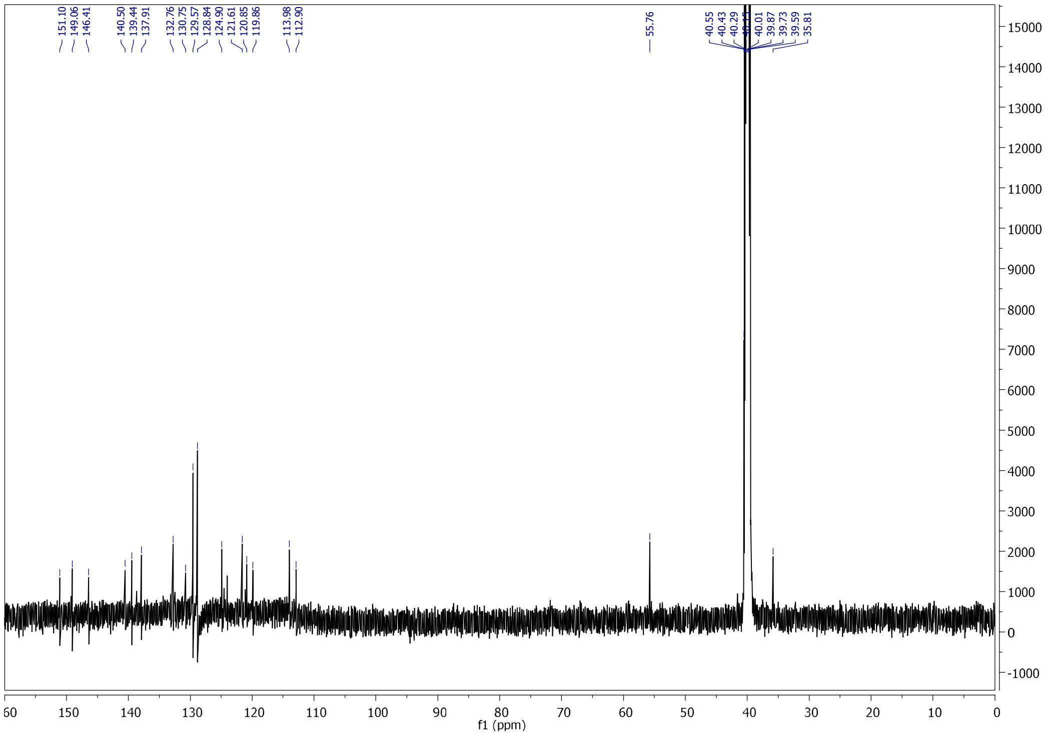

Figure S42. ^{13}C NMR of **24**

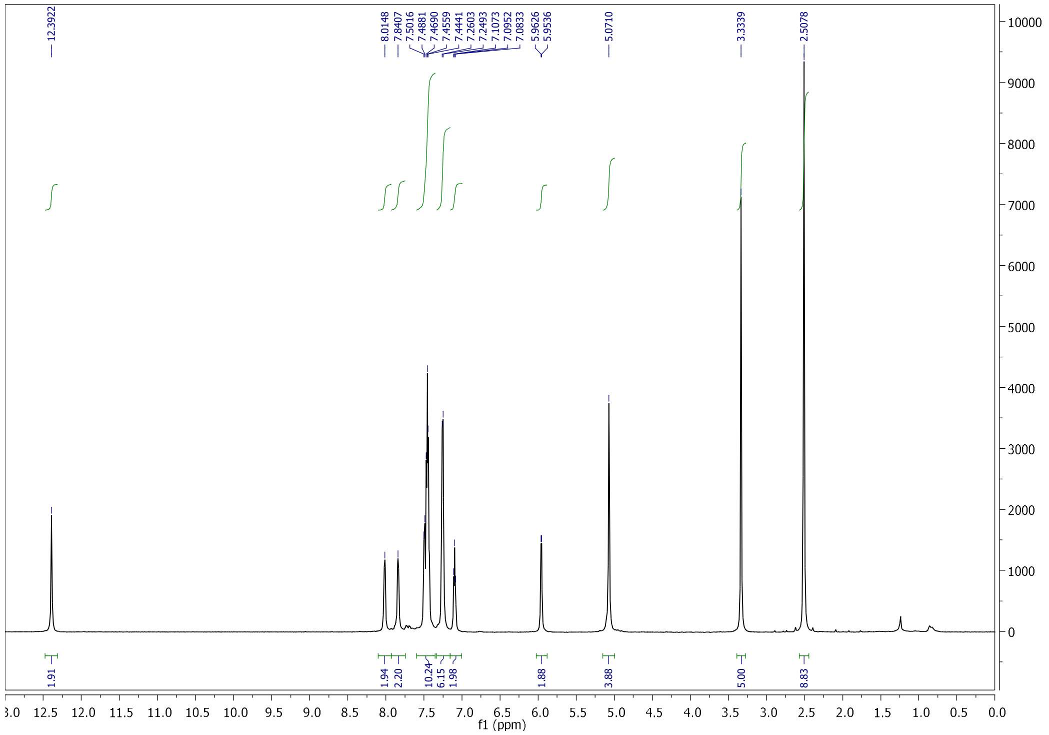

Figure S43. ^{1}H NMR of **25**

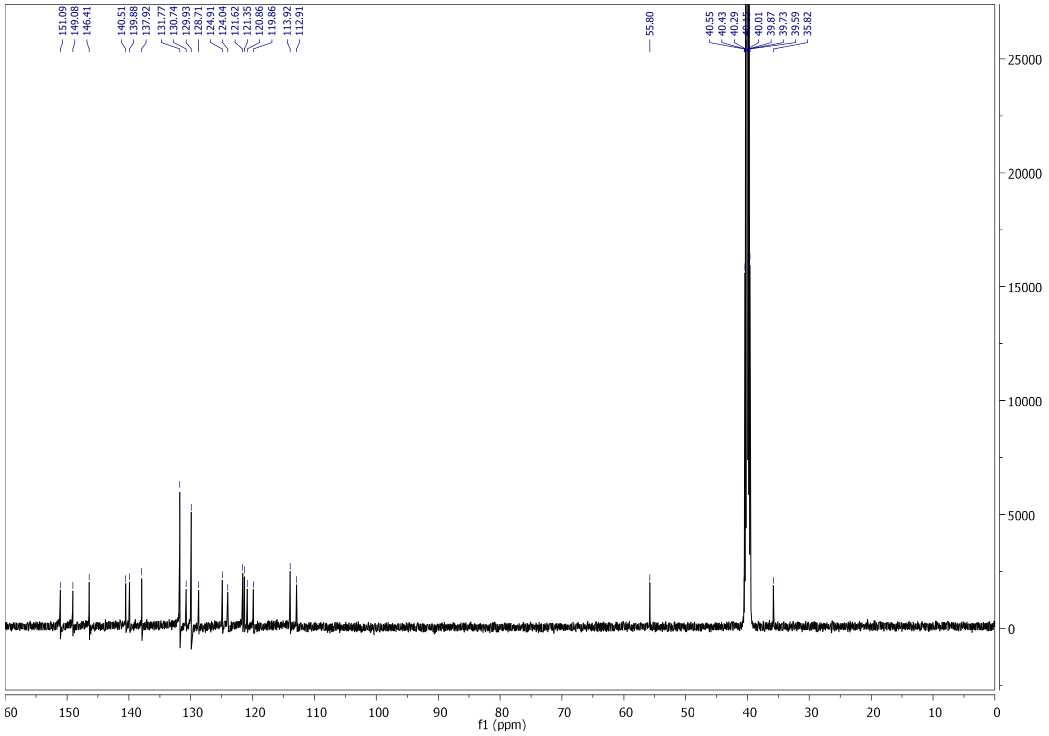

Figure S44. ^{13}C NMR of **25**

IR Spectra

IR spectra were detected using Bruker Alpha ATR-FTIR.

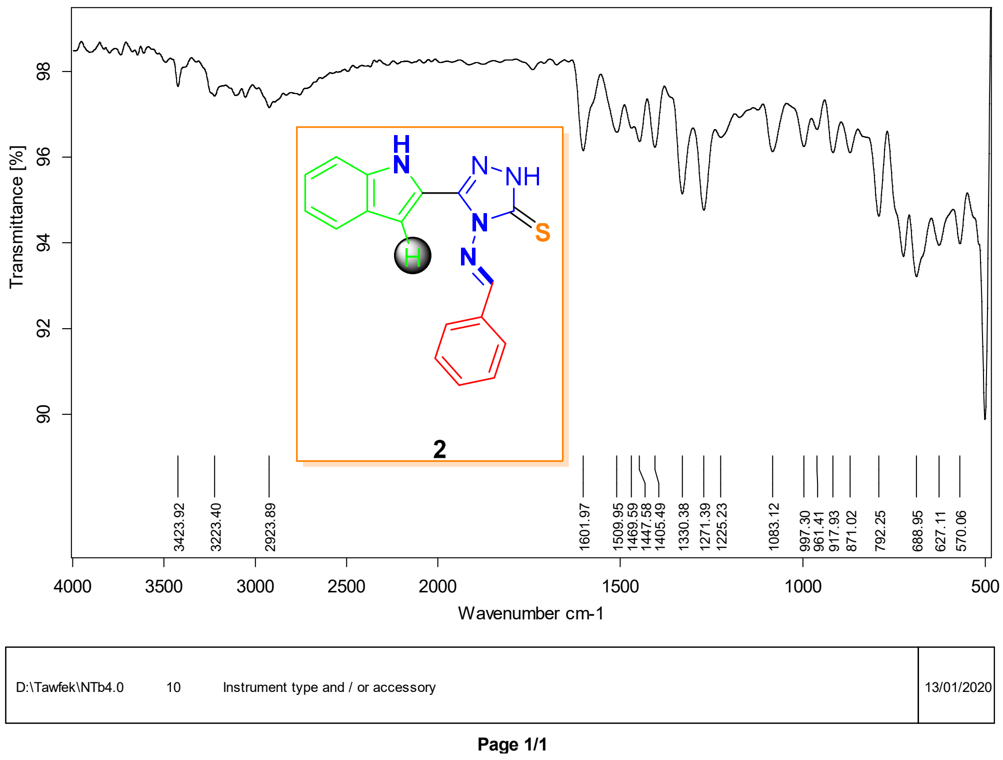

Figure S45. IR of **2**

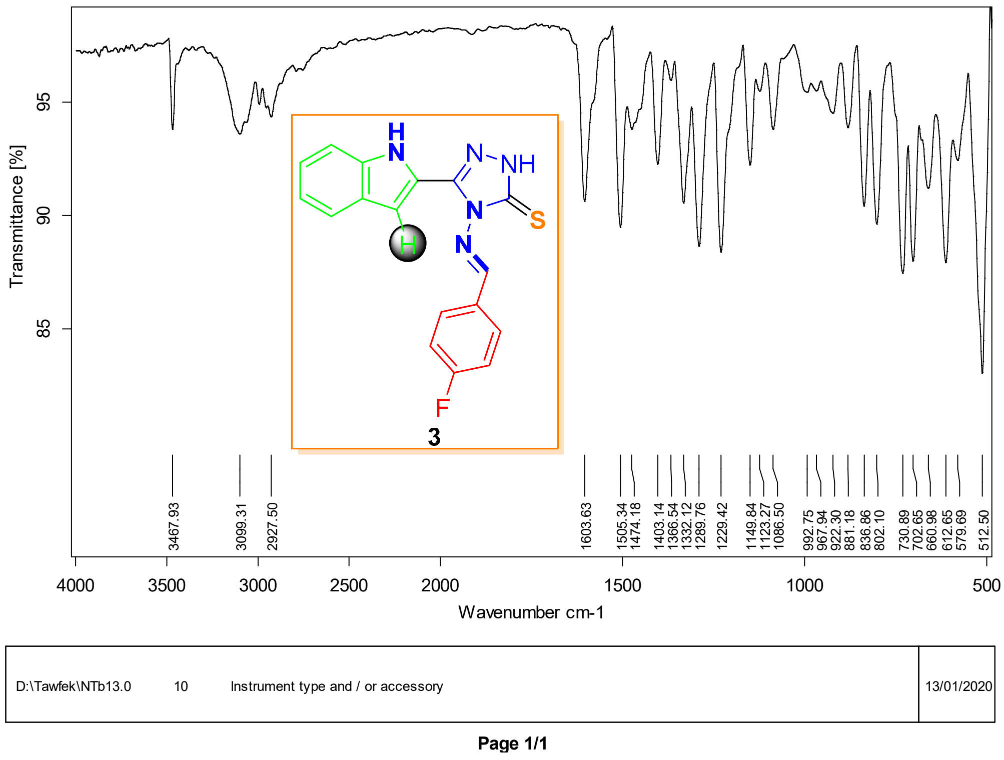

Figure S46. IR of **3**

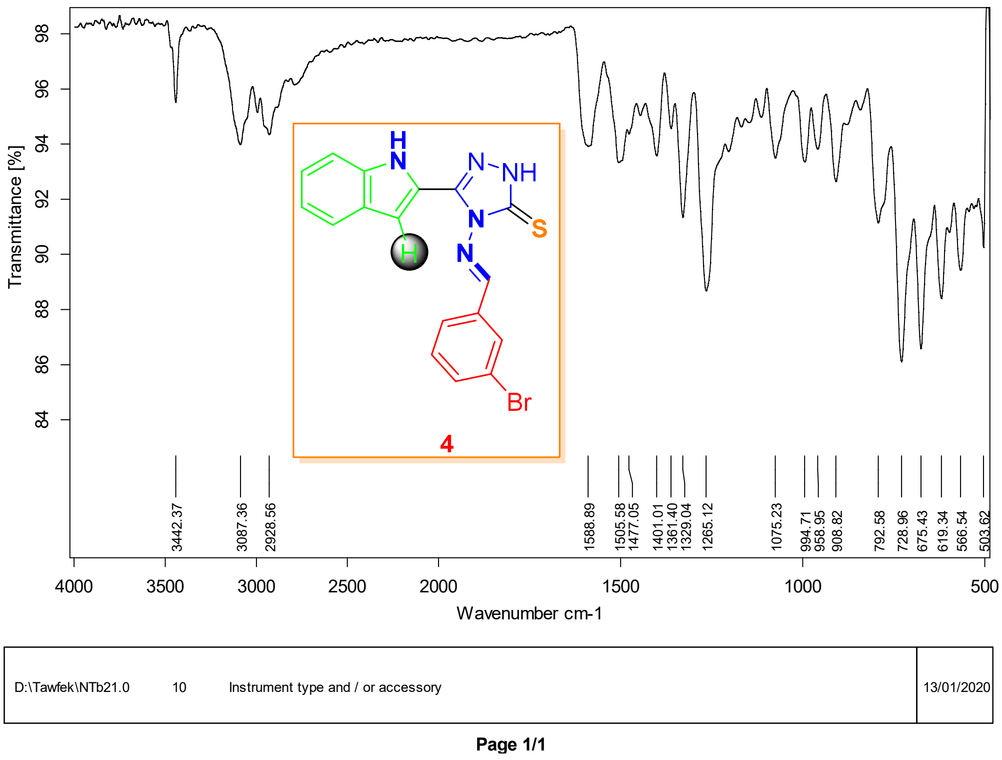

Figure S47. IR of **4**

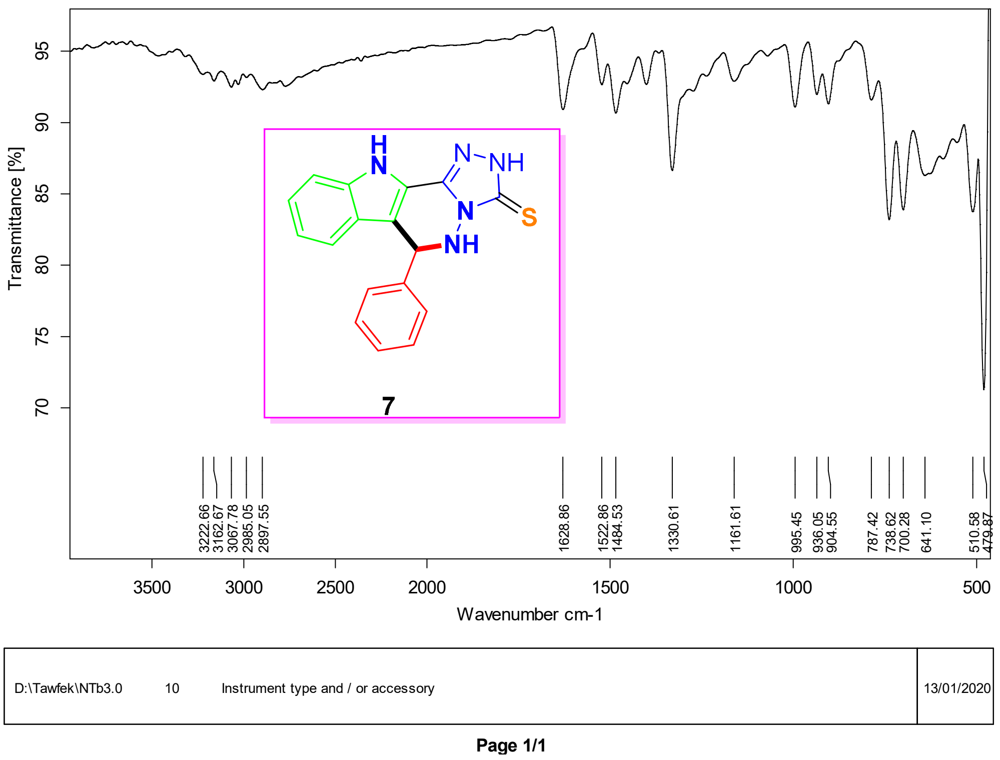

Figure S48. IR of **7**

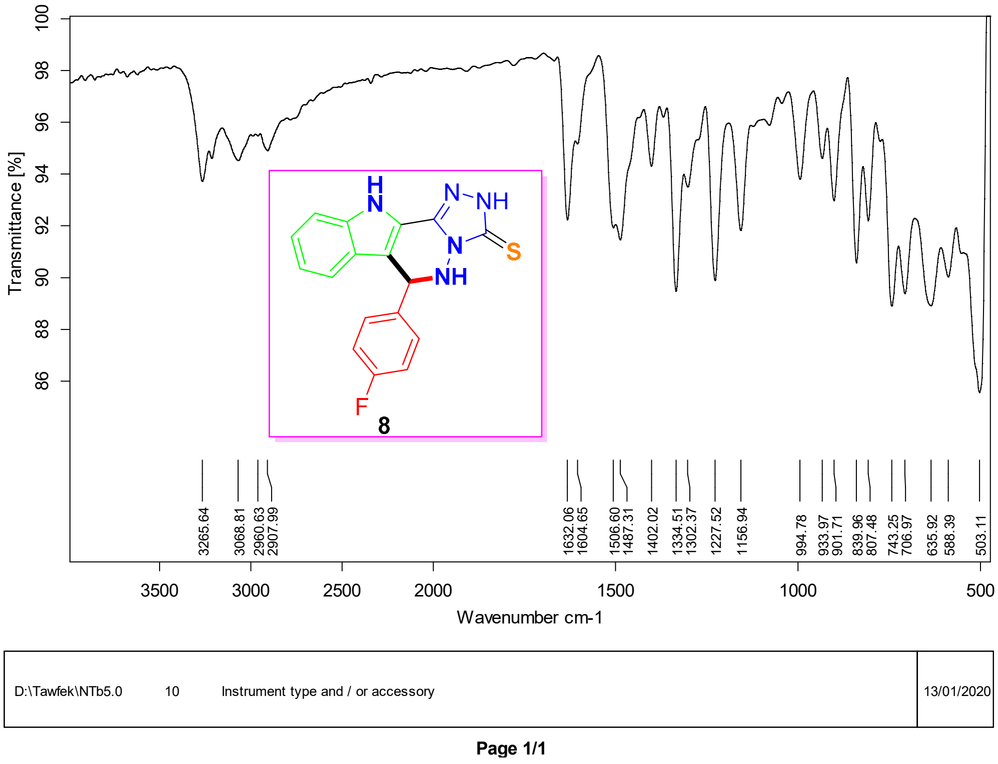

Figure S49. IR of **8**

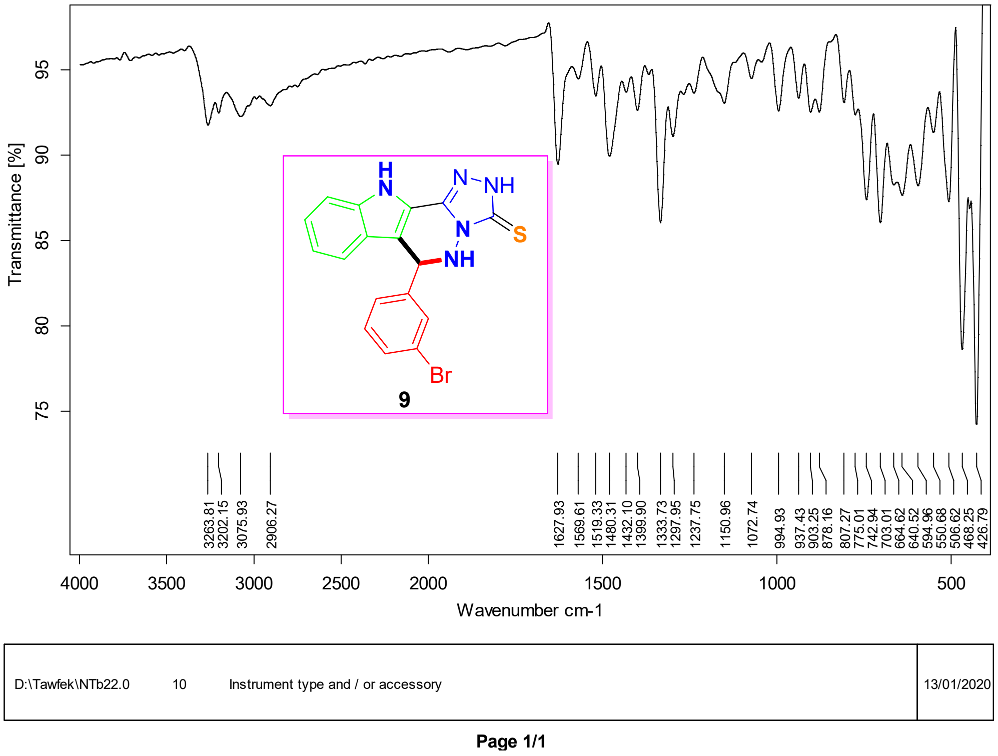

Figure S50. IR of **9**

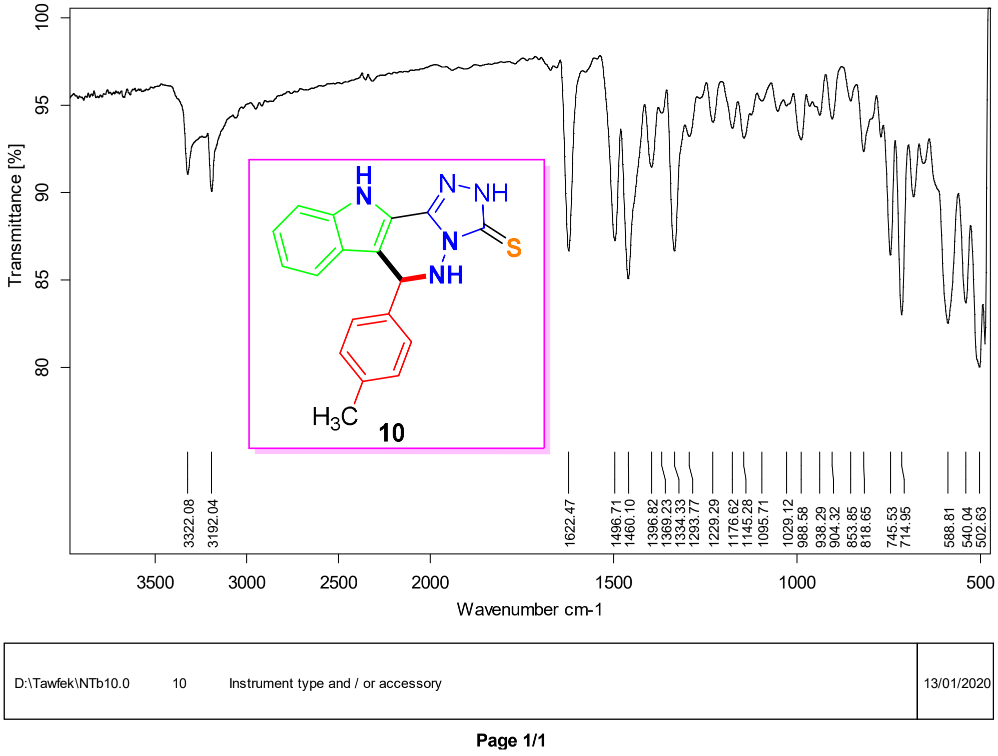

Figure S51. IR of **10**

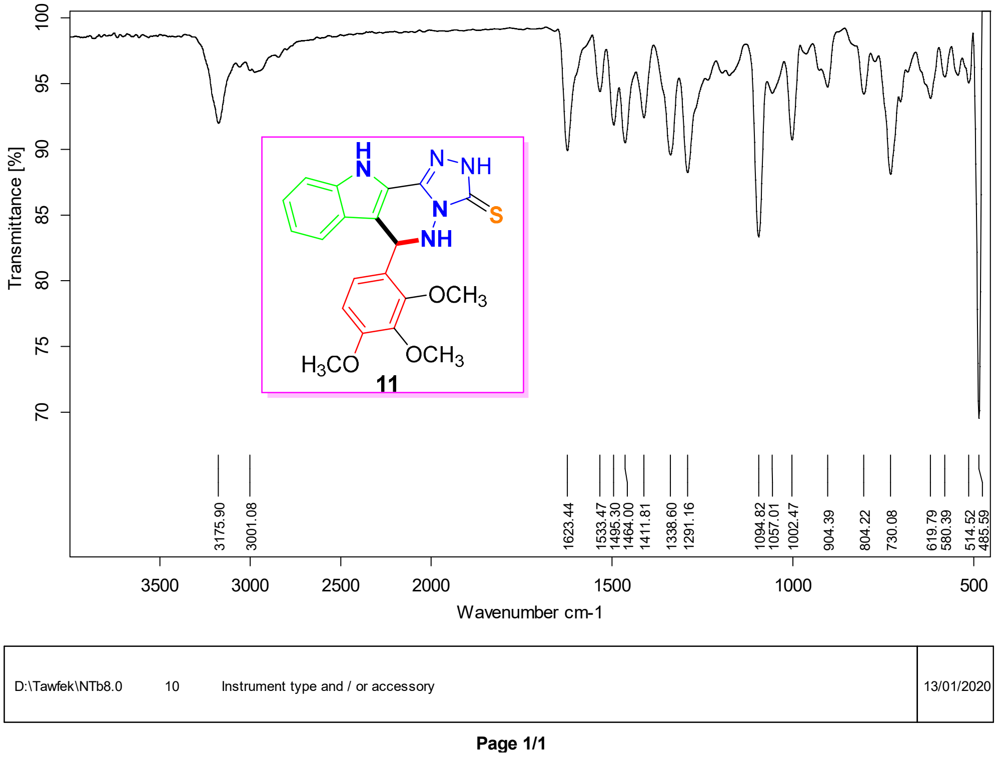

Figure S52. IR of **11**

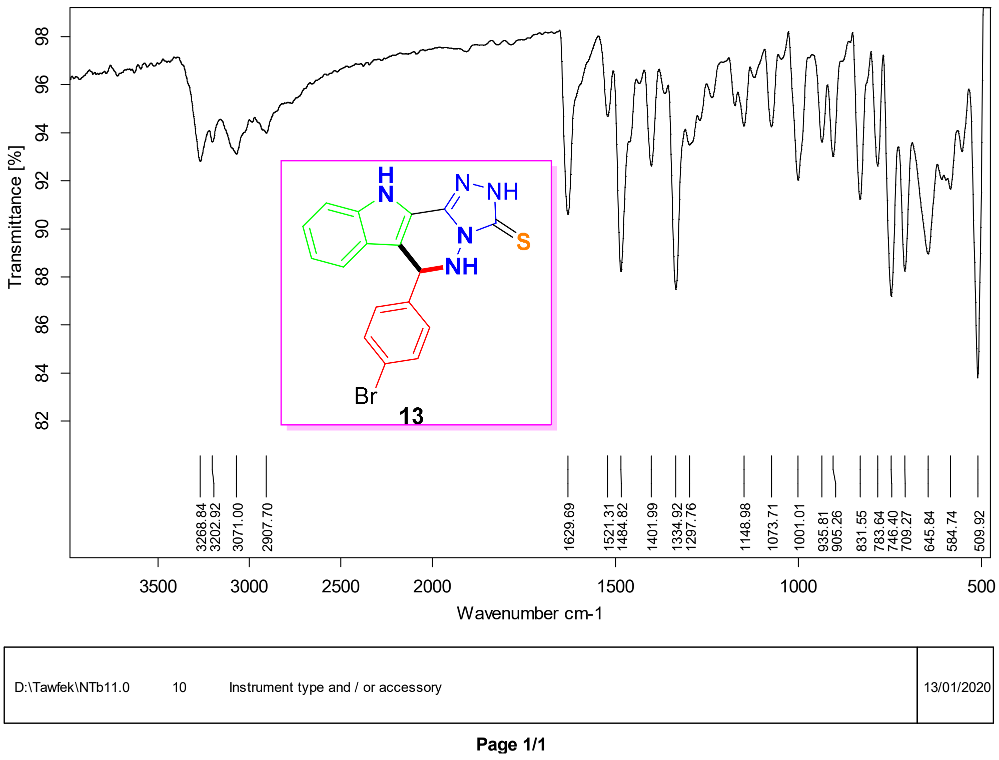

Figure S53. IR of **13**

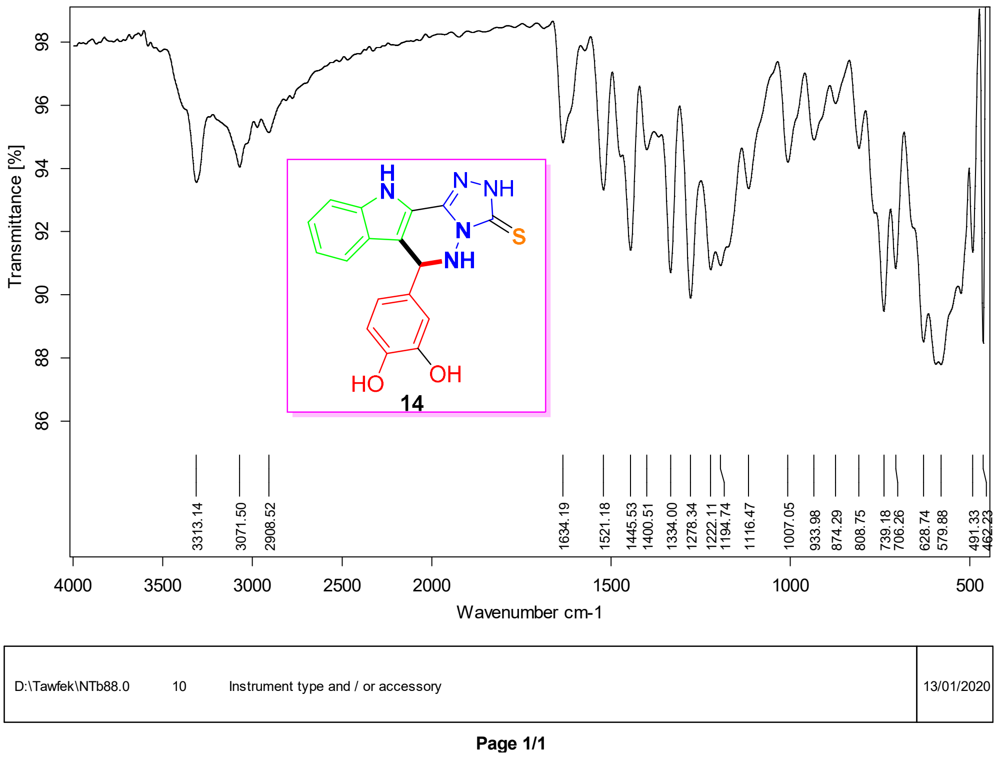

Figure S54. IR of **14**

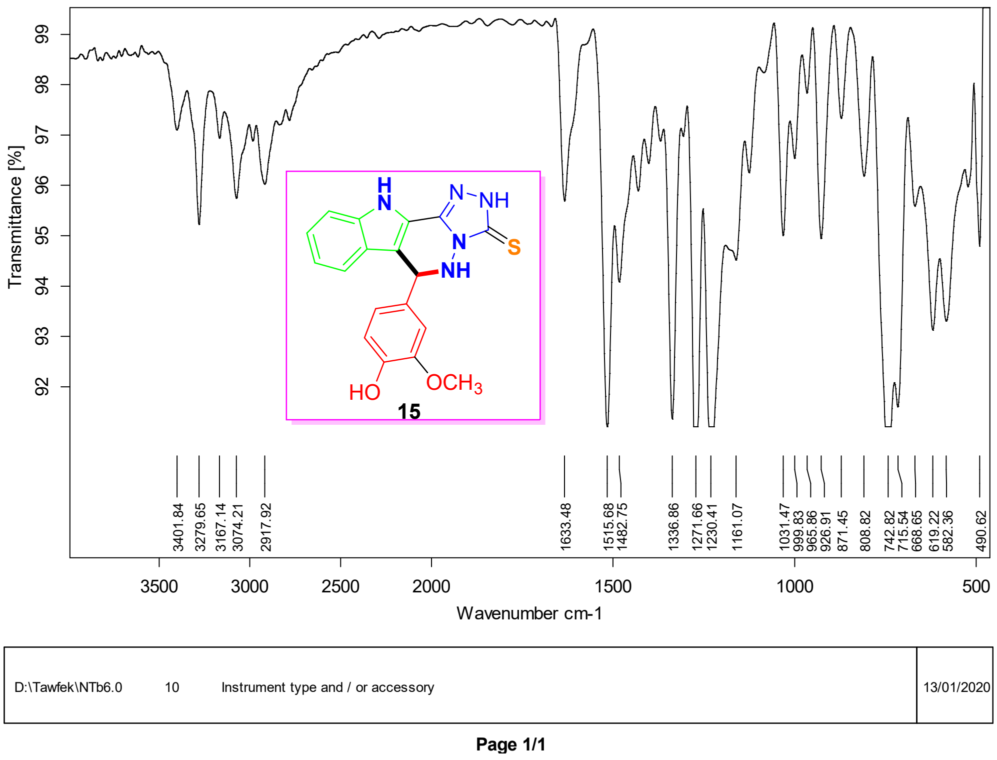

Figure S55. IR of **15**

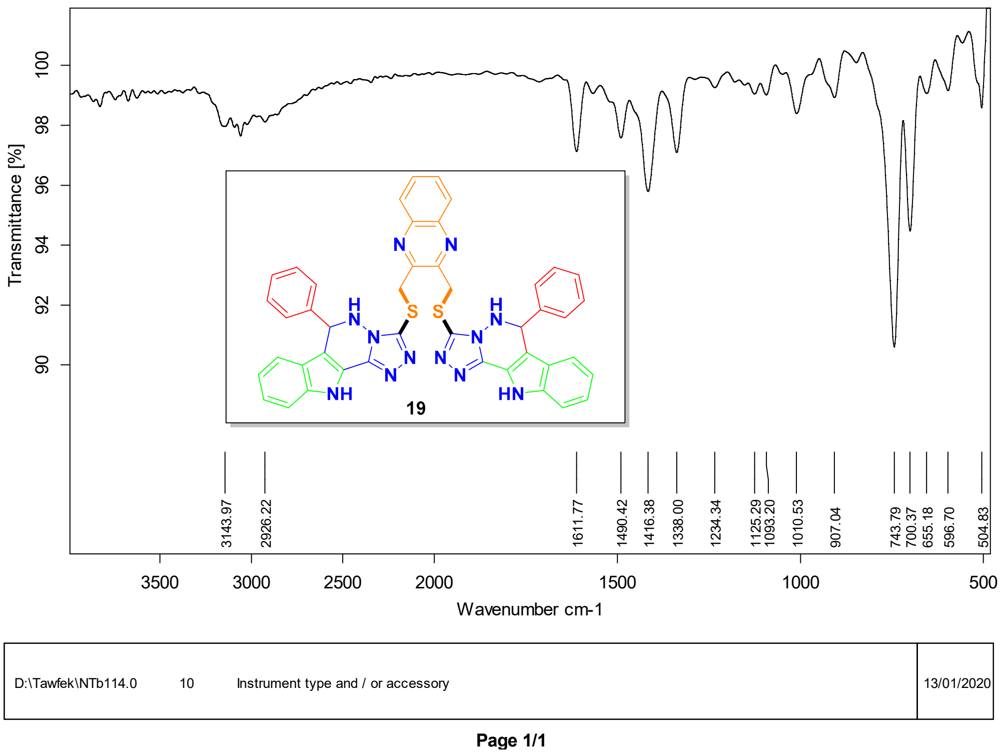

Figure S56. IR of **19**

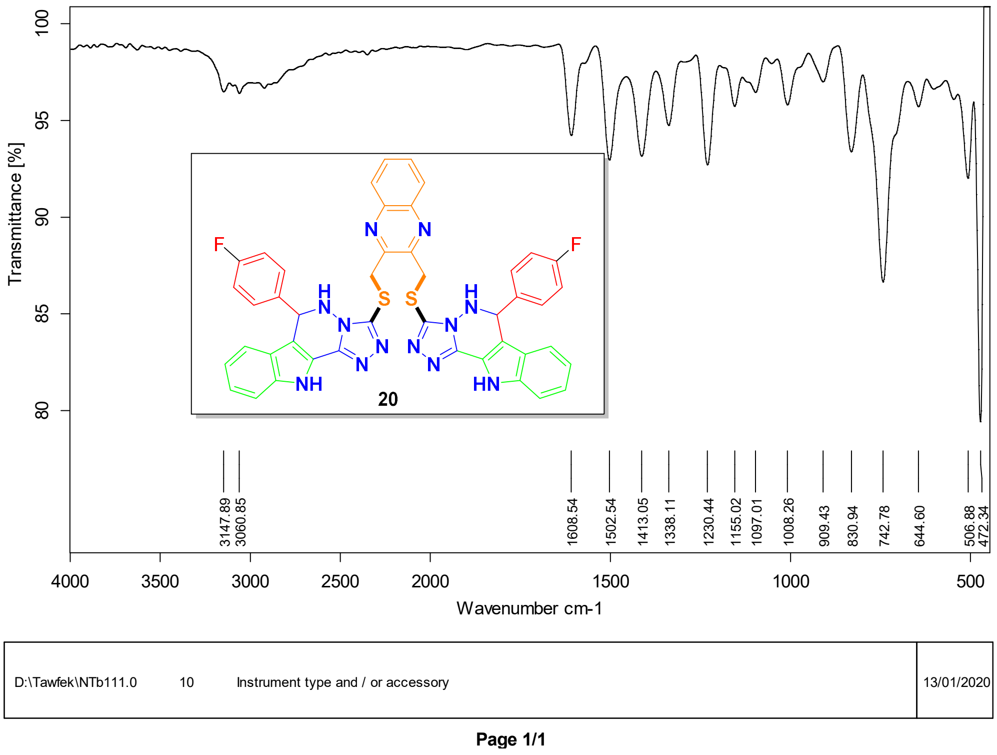

Figure S57. IR of **20**

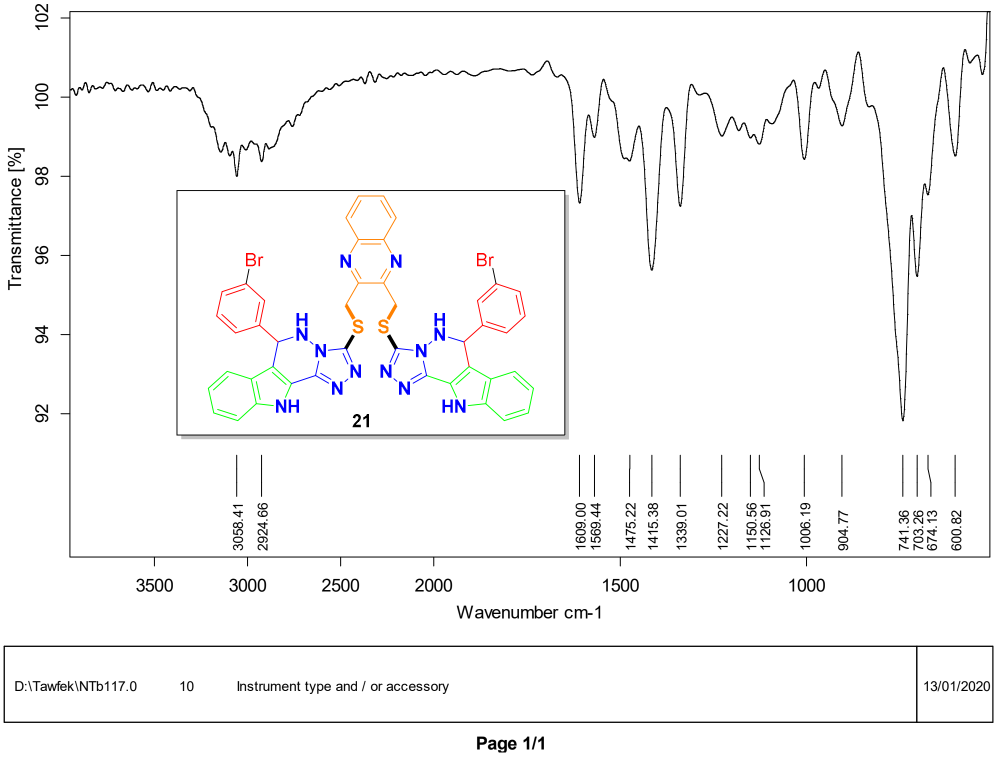

Figure S58. IR of **21**

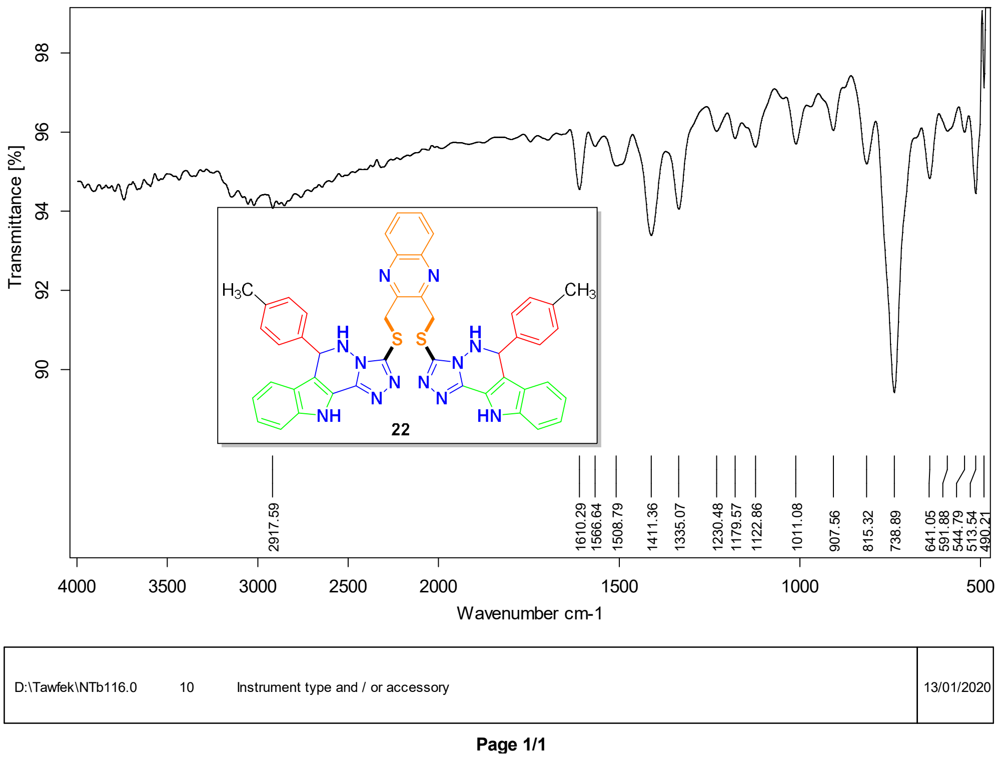

Figure S59. IR of **22**

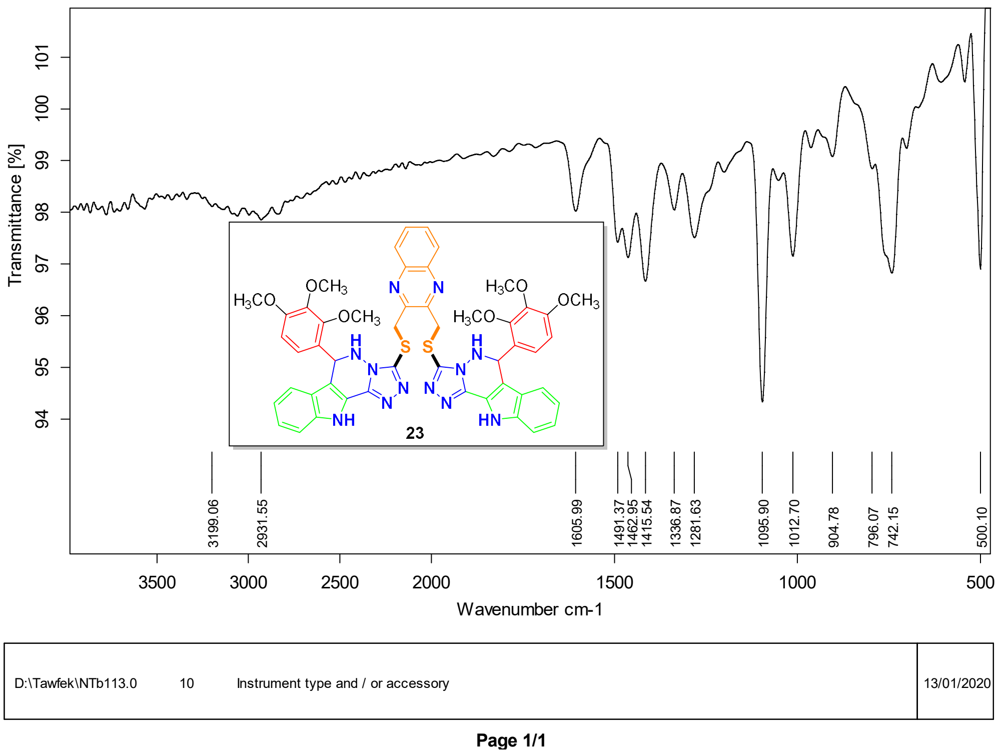

Figure S60. IR of **23**
